# Supplementary material for: Measuring under-5 mortality and fertility through mobile phone surveys: an assessment of selection bias in 34 low-income and middle-income countries
Source: BMJ Open. 2023 Nov 17;13(11):e071791. doi: 10.1136/bmjopen-2023-071791 (PMC10693685; doi:10.1136/bmjopen-2023-071791)
Supplement: Supplementary data [file bmjopen-2023-071791supp002.pdf]

Appendix Table 1: DHS including information on mobile phone ownership and access, and total fertility rate (TFR) for the 3-year period before the survey and under-five mortality rate (U5MR) for the 10-year period before the survey by mobile phone status.

		Mobile phone ownership					Access to a mobile phone				
		Owns a mobile phone			Does not own a mobile phone		Can access a mobile phone			Cannot access a mobile phone	
Survey	Sample size	% of women	TFR	U5MR	TFR	U5MR	% of women	TFR	U5MR	TFR	U5MR
Albania 2017-18	10,970	90.1	1.8	5.1	2.0	12.7	99.3	1.8	5.7	3.0	18.8
Angola 2015-16	14,379	51.2	4.7	67.9	8.0	89.1	75.3	5.5	74.0	8.4	92.3
Armenia 2015-16	6,116	96.7	1.7	10.3	3.0	15.5	99.8	1.7	10.5	*NA*	*NA*
Bangladesh 2017-18	20,127	60.2	2.2	43.9	2.3	56.6	97.2	2.2	48.7	2.7	46.7
Benin 2017-18	15,928	51.1	5.0	91.5	6.7	113.2	90.4	5.5	100.0	7.1	117.0
Burundi 2016-17	17,269	23.6	4.4	58.8	5.9	88.3	55.0	5.3	65.3	5.9	99.0
Cameroon 2018-19	13,901	63.2	4.0	72.1	6.4	115.4	91.5	4.6	85.0	6.0	126.6
Ethiopia 2016	15,683	27.3	2.8	65.8	5.2	84.1	62.1	4.0	74.9	5.5	88.7
Gambia 2019-20	11,865	76.4	4.1	56.0	6.1	75.2	99.6	4.4	60.3	8.1	69.3
Guinea 2018	10,874	68.9	4.6	98.7	5.5	129.6	94.1	4.8	105.9	6.0	141.9
Haiti 2016-17	14,371	57.1	2.3	75.9	4.4	89.8	86.3	2.7	78.1	5.3	98.9
Indonesia 2017	49,569	78.5	2.3	31.9	3.2	40.5	96.3	2.4	33.1	3.7	49.8
Jordan 2017-18	14,689	91.8	2.7	16.5	2.9	19.2	99.2	2.7	16.8	3.0	7.9
Liberia 2019-20	8,065	46.7	2.9	84.0	5.4	107.9	78.6	3.7	96.8	5.9	101.6
Madagascar 2021	18,869	34.6	3.0	60.1	5.0	72.5	61.3	3.2	63.7	5.0	71.7

Malawi 2015-16	24,562	32.8	3.2	67.7	5.1	77.9	62.4	3.8	69.9	5.4	81.4
Maldives 2016-17	7,699	95.5	2.1	21.5	2.4	27.7	100.0	2.1	21.6	*NA*	*NA*
Mali 2018	10,519	58.3	5.5	97.1	7.4	130.1	94.3	6.2	111.8	7.4	126.3
Mauritania 2019-21	15,714	76.7	4.9	40.6	6.8	49.5	96.1	5.1	41.7	7.5	57.7
Nepal 2016	12,862	72.6	2.2	40.3	3.1	62.8	97.3	2.3	45.3	4.0	64.9
Nigeria 2018	41,821	55.3	4.4	93.0	6.5	163.6	92.6	5.2	123.3	6.6	181.5
Pakistan 2017-18	12,363	39.2	3.2	64.4	3.8	85.1	95.4	3.5	77.0	4.1	88.8
Papua New Guinea 2016-18	15,141	34.3	3.2	40.6	4.7	56.2	66.6	3.7	48.5	5.1	56.4
Philippines 2017	25,074	85.8	2.5	24.6	4.3	41.4	95.5	2.5	27.0	5.2	36.8
Rwanda 2019-20	14,634	47.9	3.4	38.6	5.1	59.3	79.1	3.9	44.4	5.1	64.4
Senegal 2019	8,649	69.7	4.2	44.3	6.3	57.1	98.9	4.7	47.5	5.9	98.7
Sierra Leone 2019	15,574	42.6	3.1	110.3	5.2	132.6	81.4	3.9	118.2	5.9	142.0
South Africa 2016	8,514	91.2	2.6	51.2	4.1	43.7	98.8	2.6	51.2	4.1	10.5
Tajikistan 2017	10,718	53.7	3.5	31.5	4.1	35.6	98.1	3.7	32.6	5.1	64.4
Tanzania 2015-16	13,266	52.2	4.1	75.4	6.7	80.8	86.2	4.9	75.6	6.8	88.8
Timor-Leste 2016	12,607	65.6	3.9	35.5	5.0	49.4	94.3	4.2	40.8	5.1	38.1
Uganda 2016	18,506	45.5	4.3	65.2	6.7	79.7	81.8	5.1	68.6	6.8	89.7
Zambia 2018-19	13,683	53.0	3.7	65.4	6.1	63.4	81.2	4.2	62.7	6.6	69.1
Zimbabwe 2015	9,955	69.5	3.7	75.6	5.1	99.3	92.2	3.9	79.8	5.8	102.2

Note: Fertility and mortality rates for women who cannot access a mobile phone cannot be computed for Armenia and Maldives due to the small sample size.
