# Supplementary material for: Measuring under-5 mortality and fertility through mobile phone surveys: an assessment of selection bias in 34 low-income and middle-income countries
Source: BMJ Open. 2023 Nov 17;13(11):e071791. doi: 10.1136/bmjopen-2023-071791 (PMC10693685; doi:10.1136/bmjopen-2023-071791)

Appendix Figure 1.1: Effect of the 15-19 age group on the odds of owning and having access to a mobile phone from binomial logistic regressions (20-24 age group is the reference). X-axis in log scale.

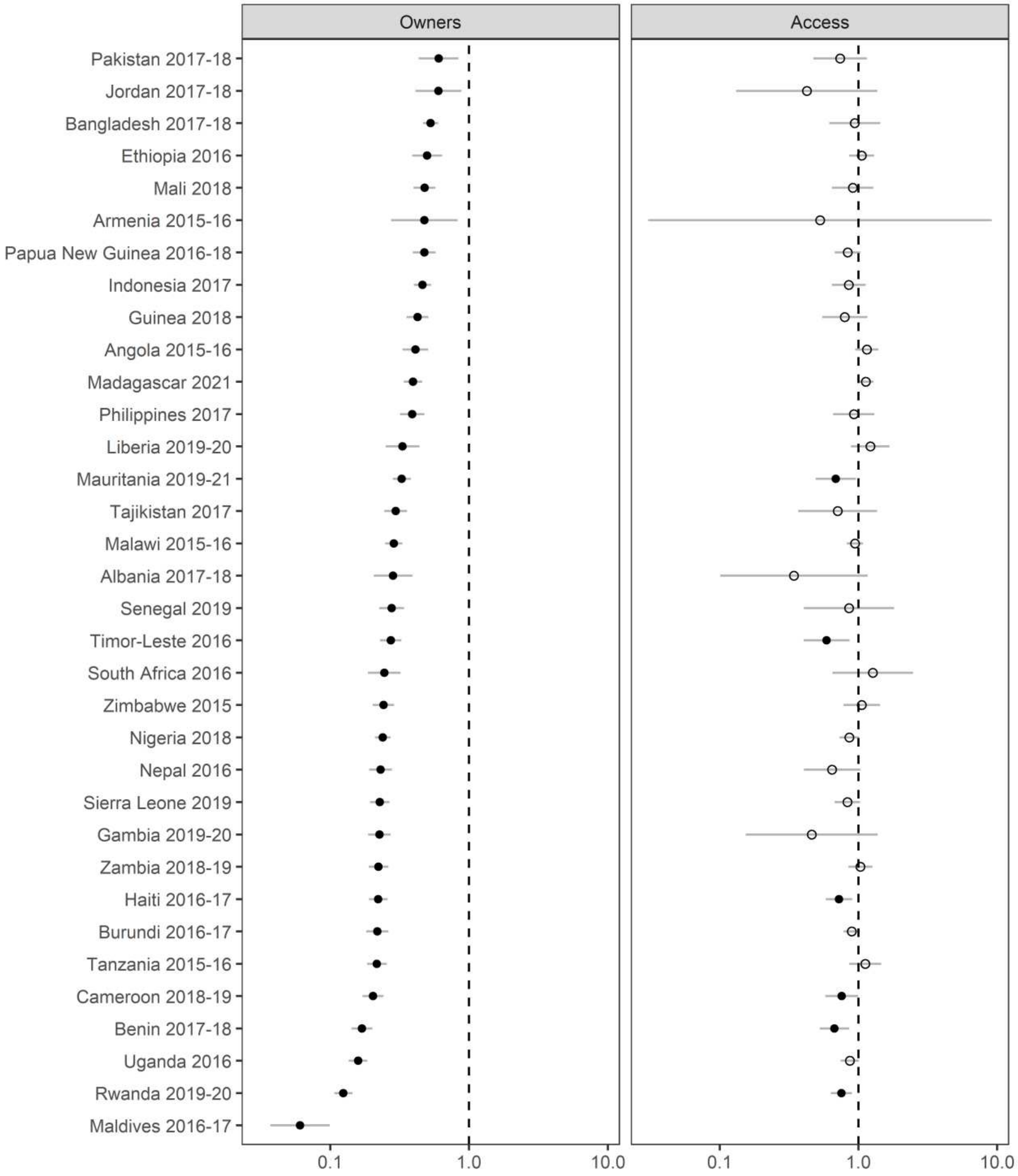

Note: Estimates for access to a mobile phone cannot be computed for Maldives due to small sample size. Fill dots mean that estimates are statistically significant at the 95% confidence level, while empty dots mean that estimates are not statistically significant.

Appendix Figure 1.2: Effect of the 25-29 age group on the odds of owning and having access to a mobile phone from binomial logistic regressions (20-24 age group is the reference). X-axis in log scale.

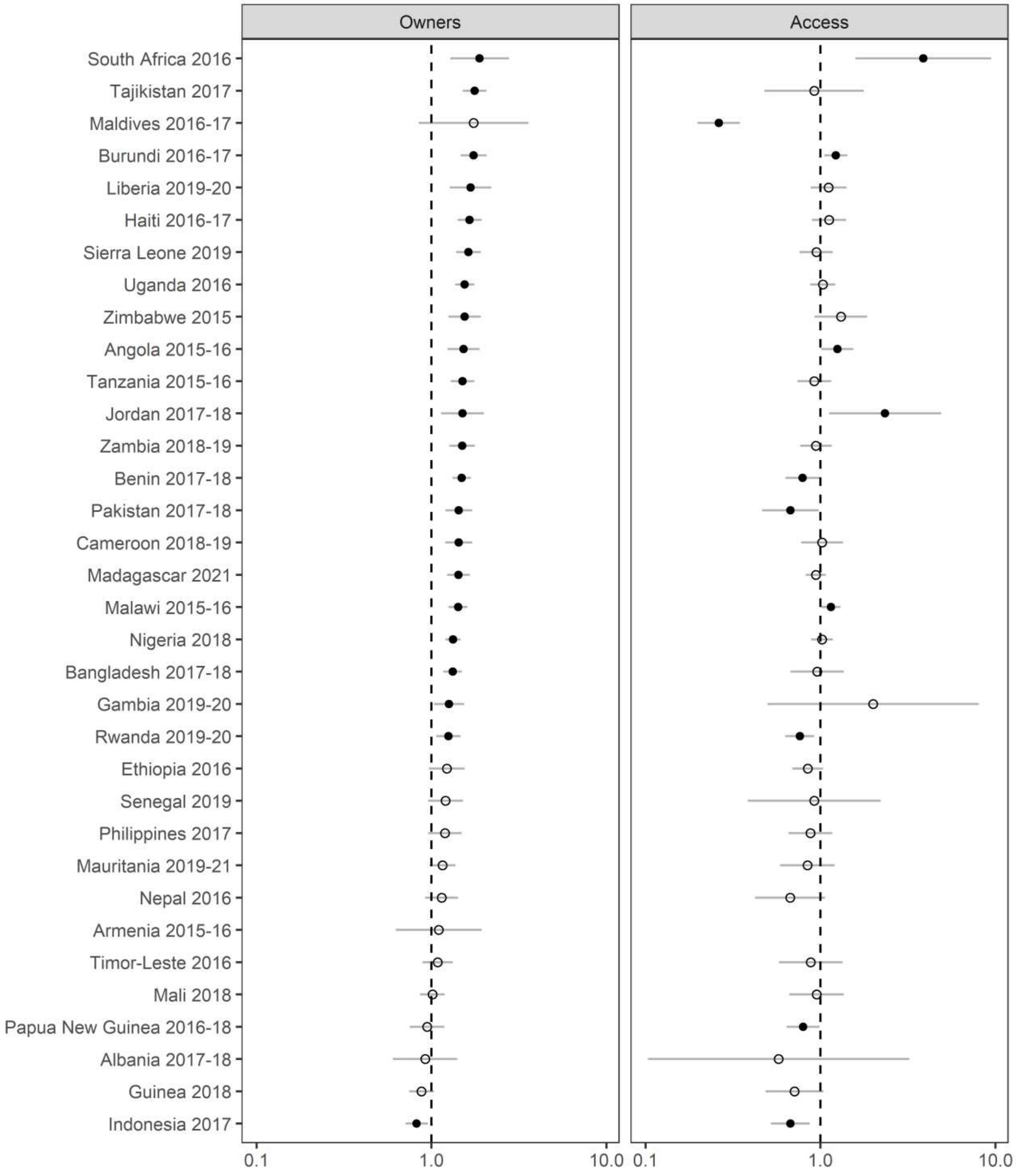

Note: Estimates for access to a mobile phone cannot be computed for Armenia due to small sample size. Fill dots mean that estimates are statistically significant at the 95% confidence level, while empty dots mean that estimates are not statistically significant.

Appendix Figure 1.3: Effect of the 30-34 age group on the odds of owning and having access to a mobile phone from binomial logistic regressions (20-24 age group is the reference). X-axis in log scale.

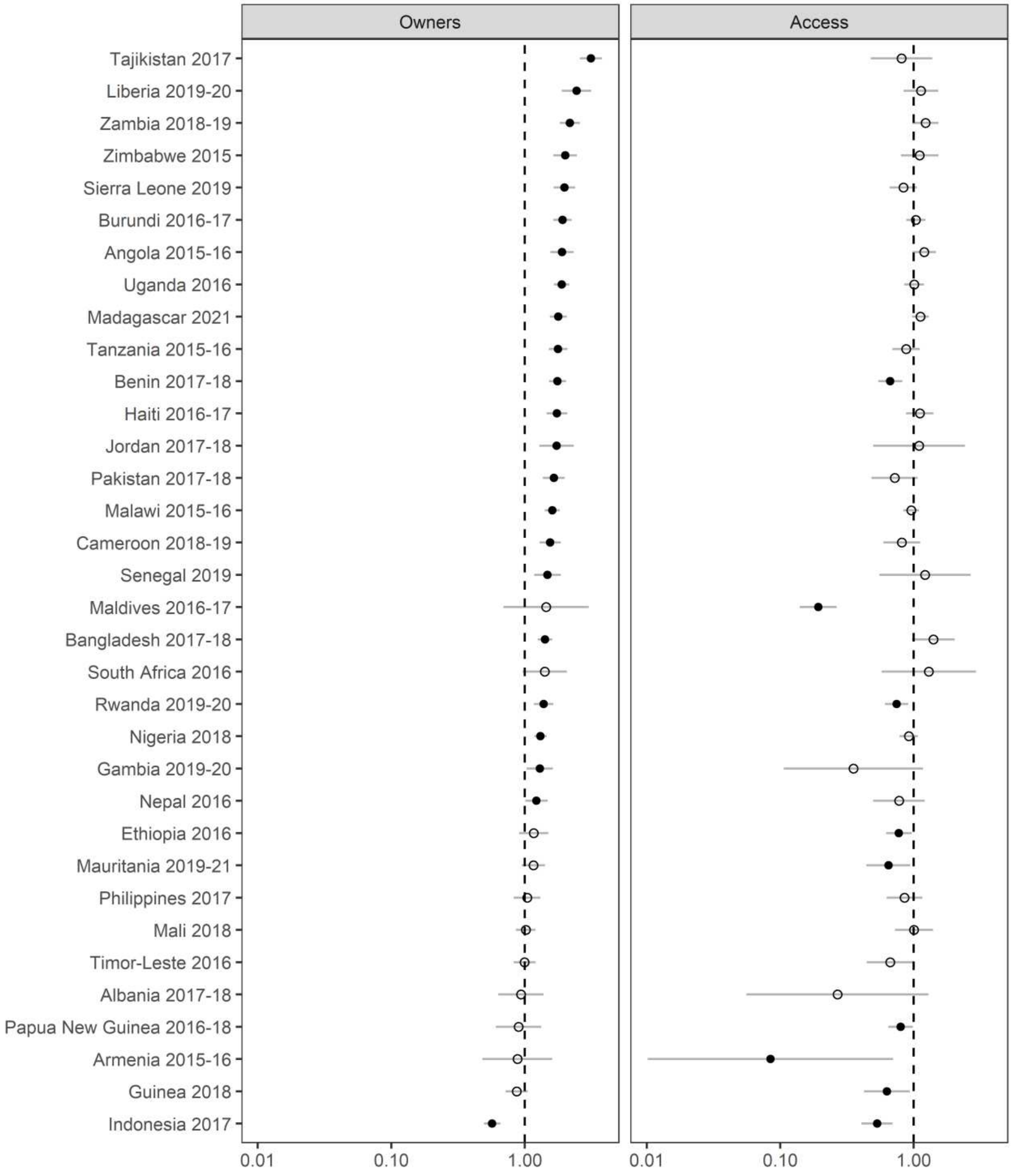

Note: Fill dots mean that estimates are statistically significant at the 95% confidence level, while empty dots mean that estimates are not statistically significant.

Appendix Figure 1.4: Effect of the 35-39 age group on the odds of owning and having access to a mobile phone from binomial logistic regressions (20-24 age group is the reference). X-axis in log scale.

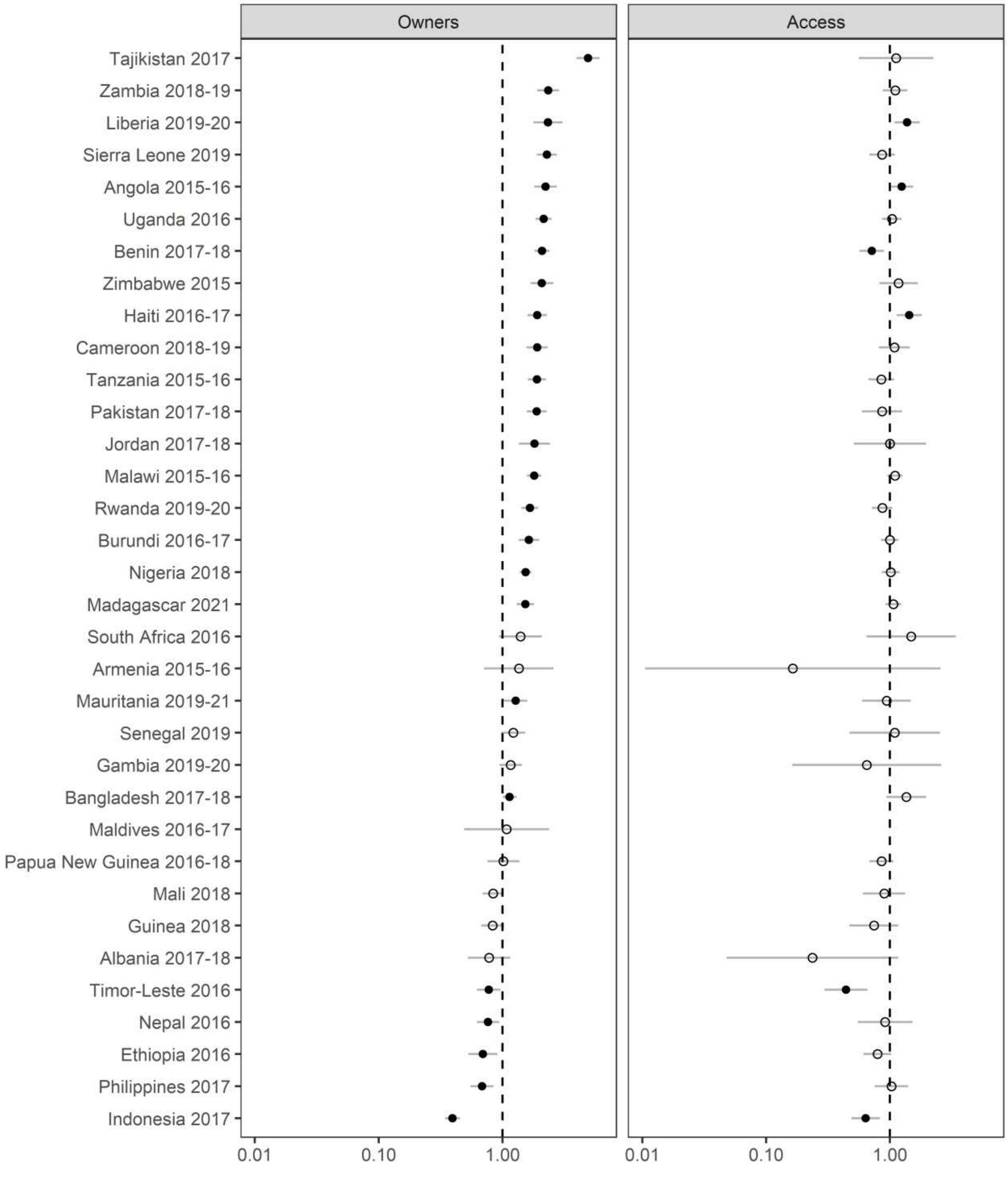

Note: Estimates for access to a mobile phone cannot be computed for Maldives due to small sample size. Fill dots mean that estimates are statistically significant at the 95% confidence level, while empty dots mean that estimates are not statistically significant.

Appendix Figure 1.5: Effect of the 40-44 age group on the odds of owning and having access to a mobile phone from binomial logistic regressions (20-24 age group is the reference). X-axis in log scale.

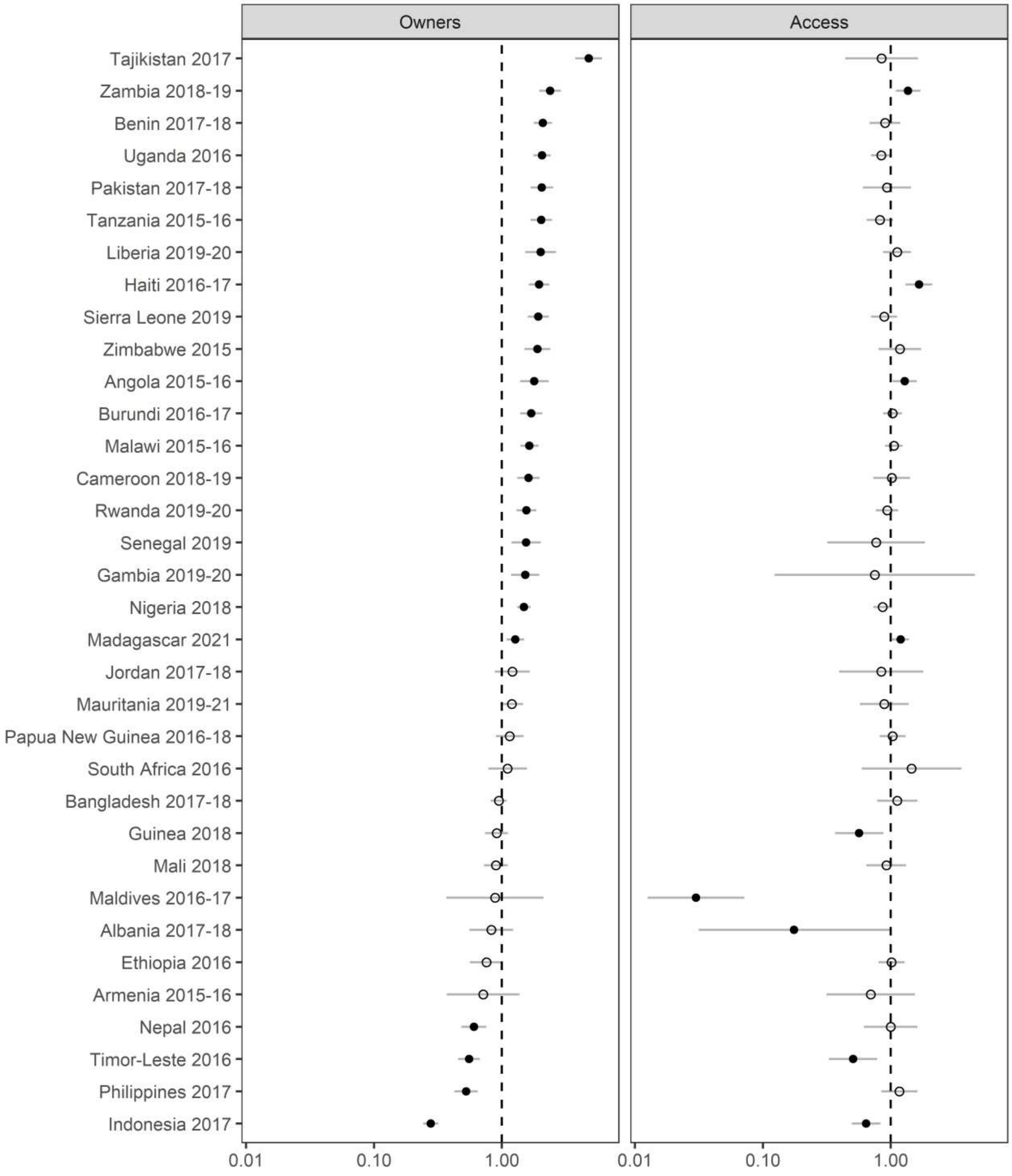

Note: Fill dots mean that estimates are statistically significant at the 95% confidence level, while empty dots mean that estimates are not statistically significant.

Appendix Figure 1.6: Effect of the 45-49 age group on the odds of owning and having access to a mobile phone from binomial logistic regressions (20-24 age group is the reference). X-axis in log scale.

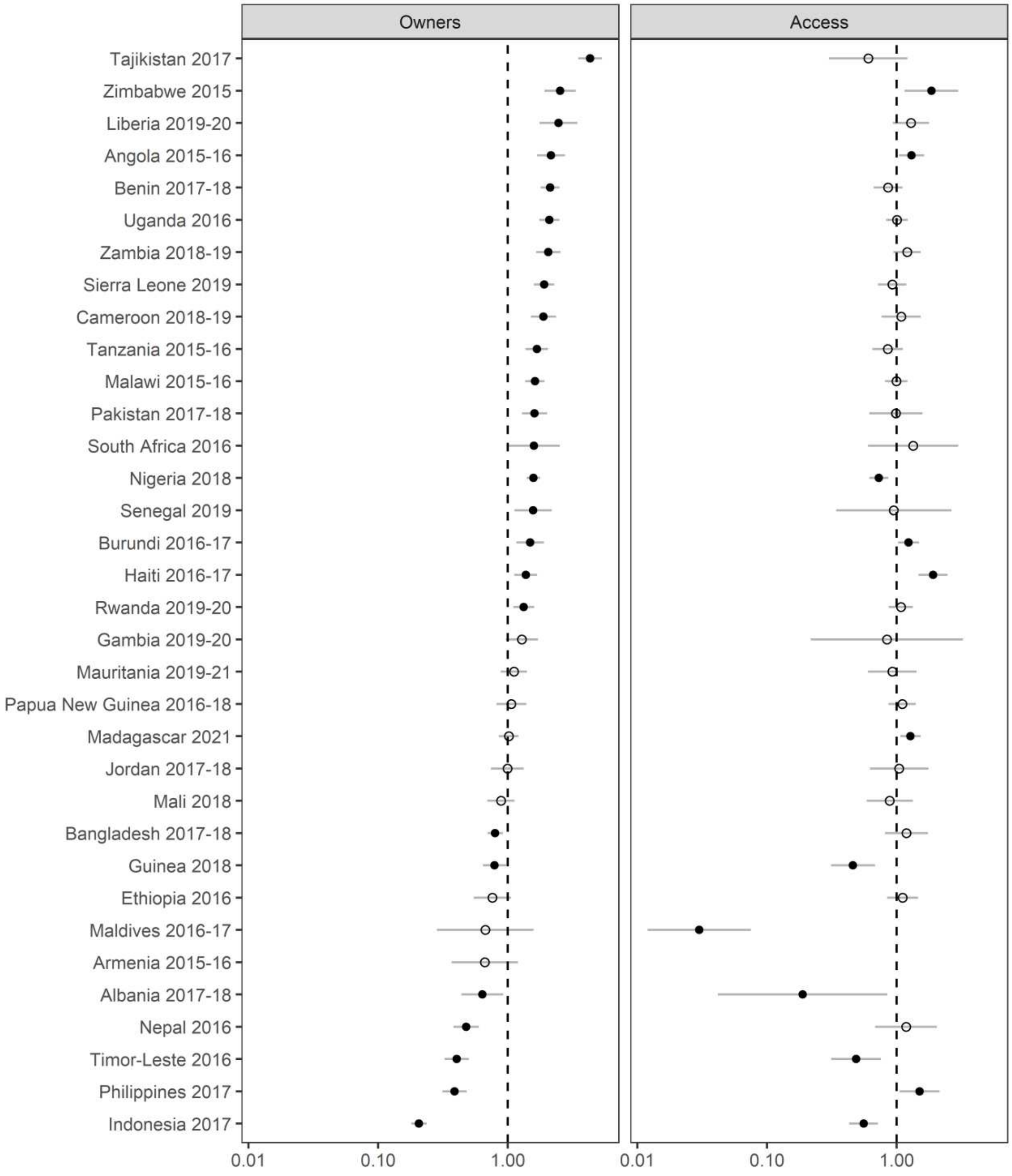

Note: Estimates for access to a mobile phone cannot be computed for Armenia due to small sample size. Fill dots mean that estimates are statistically significant at the 95% confidence level, while empty dots mean that estimates are not statistically significant.

Appendix Figure 2.1: Effect of primary education on the odds of owning and having access to a mobile phone from binomial logistic regressions (no education is the reference). X-axis in log scale.

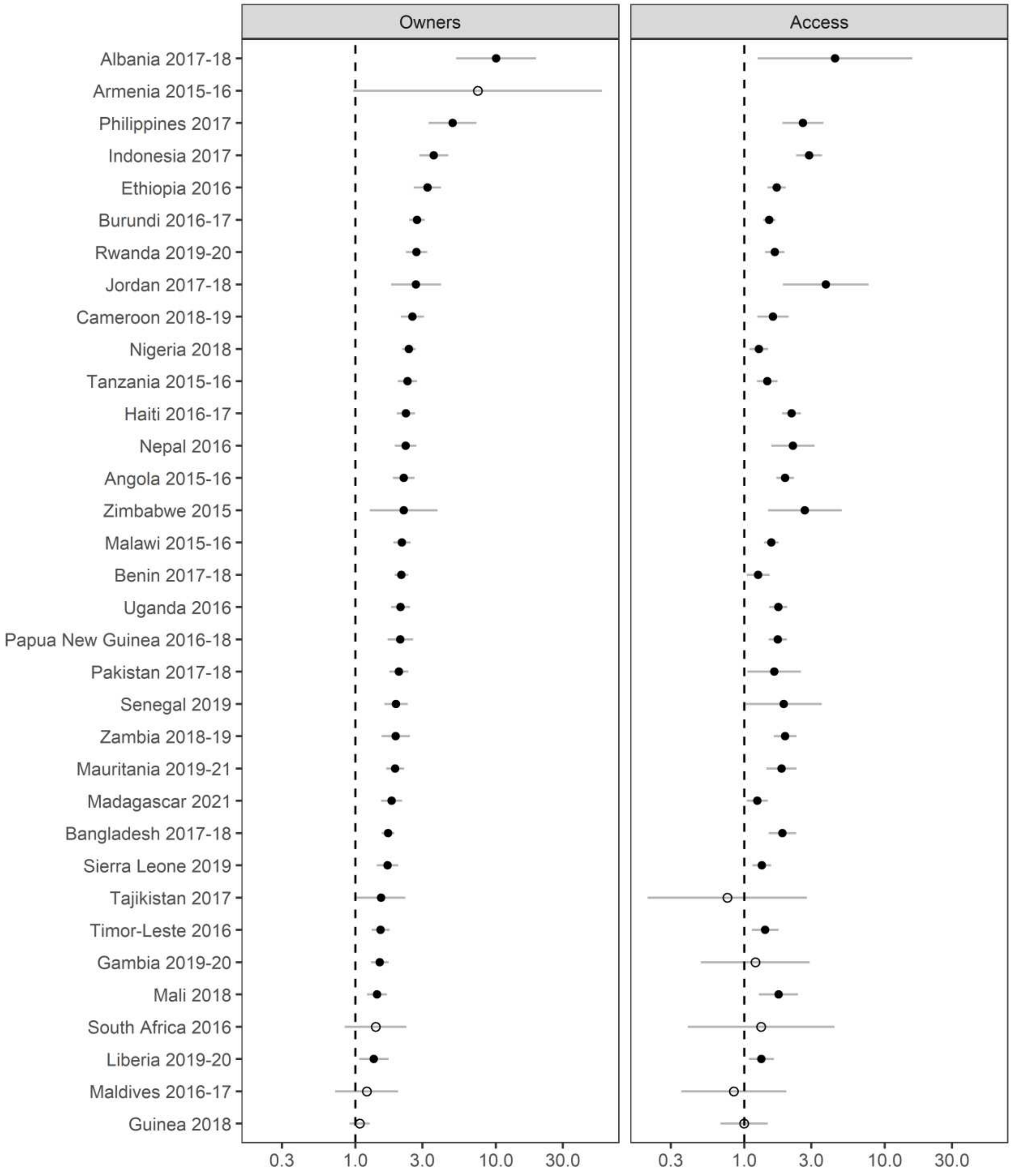

Note: Estimates for access to a mobile phone cannot be computed for Armenia due to small sample size. Fill dots mean that estimates are statistically significant at the 95% confidence level, while empty dots mean that estimates are not statistically significant.

Appendix Figure 2.2: Effect of secondary and higher education on the of odds owning and having access to a mobile phone from binomial logistic regressions (no education is the reference). X-axis in log scale.

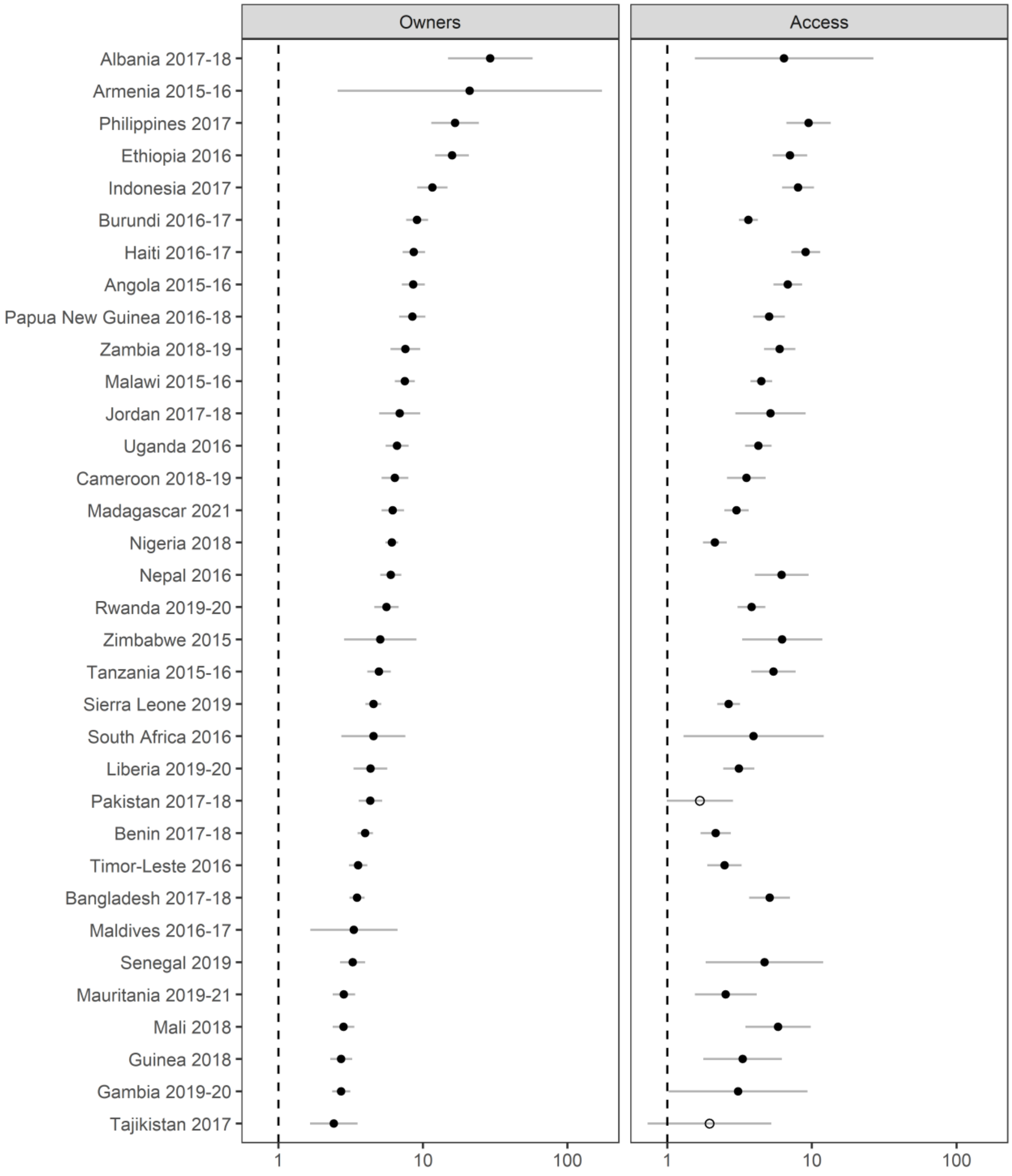

Note: Estimates for access to a mobile phone cannot be computed for Armenia and Maldives due to small sample size. Fill dots mean that estimates are statistically significant at the 95% confidence level, while empty dots mean that estimates are not statistically significant.

Appendix Figure 3: Effect of living in urban areas on the odds of owning and having access to a mobile phone from binomial logistic regressions (rural area is the reference). X-axis in log scale.

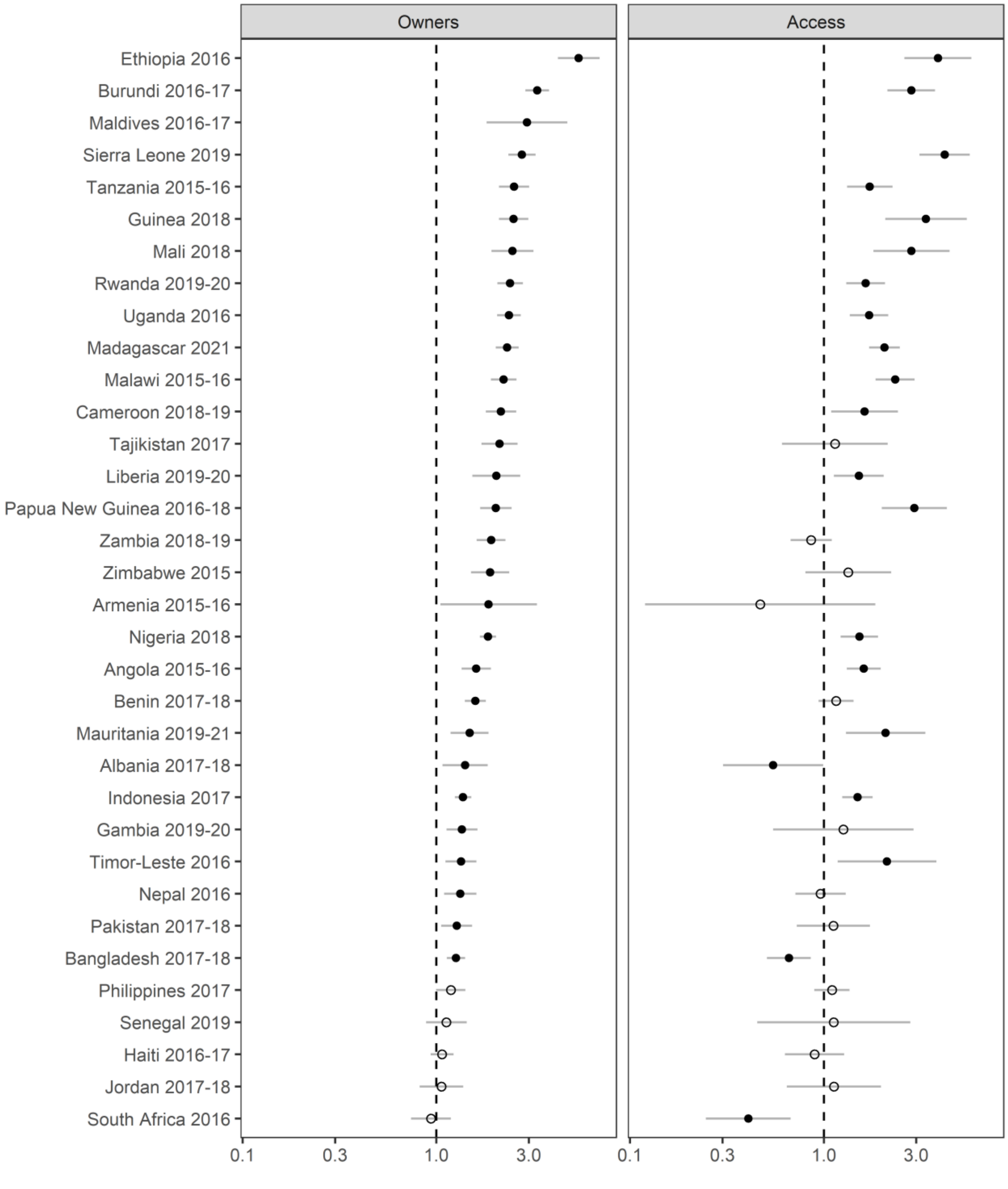

Note: Estimates for access to a mobile phone cannot be computed for Maldives due to small sample size. Fill dots mean that estimates are statistically significant at the 95% confidence level, while empty dots mean that estimates are not statistically significant.

Appendix Figure 4: Effect of being non-poor on the odds of owning and having access to a mobile phone from binomial logistic regressions (poor is the reference). X-axis in log scale.

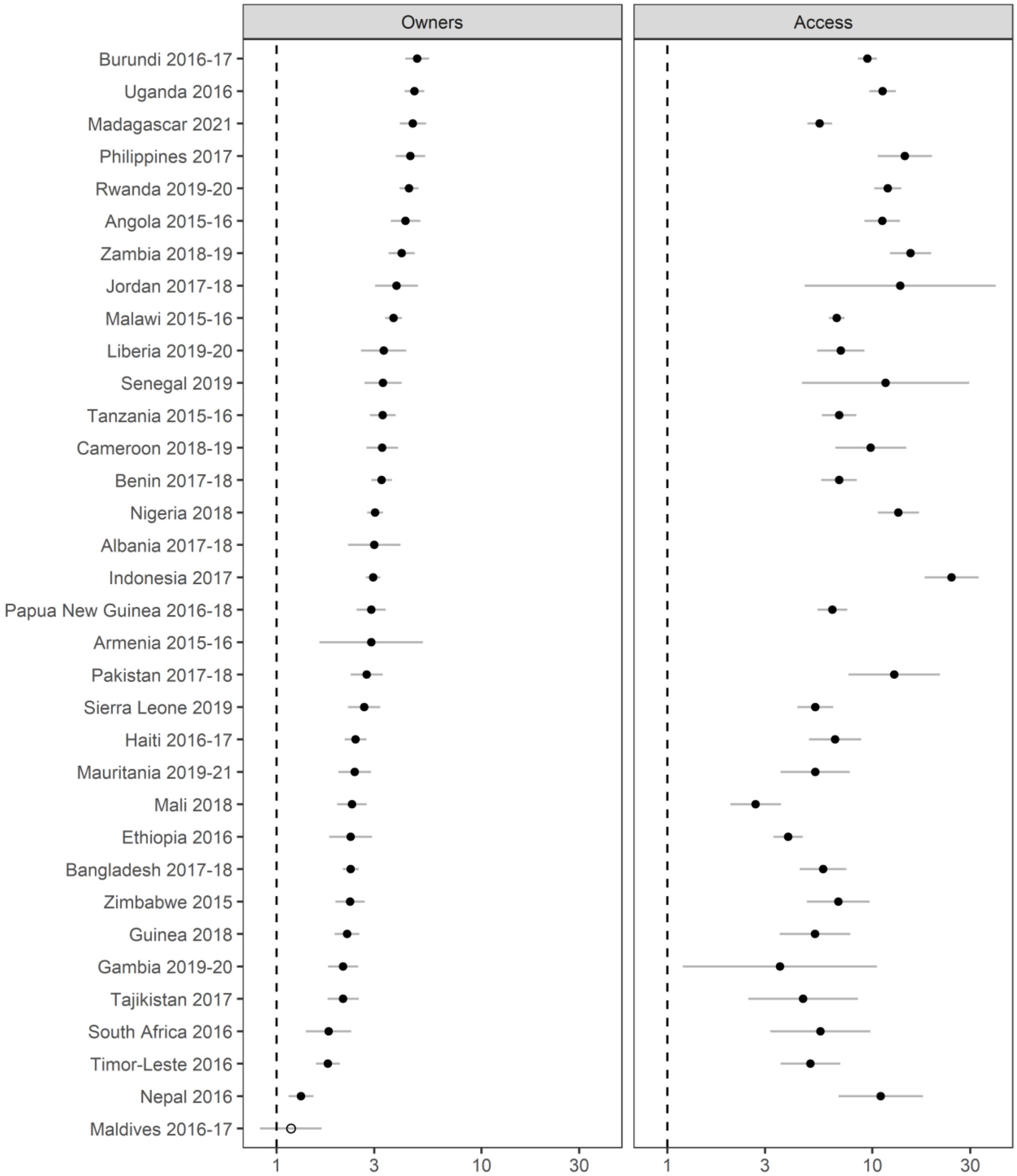

Note: Estimates for access to a mobile phone cannot be computed for Albania, Armenia, and Maldives due to small sample size. Fill dots mean that estimates are statistically significant at the 95% confidence level, while empty dots mean that estimates are not statistically significant.

Appendix Figure 5: Effect of not being in union on the odds of owning and having access to a mobile phone from binomial logistic regressions (married is the reference). X-axis in log scale.

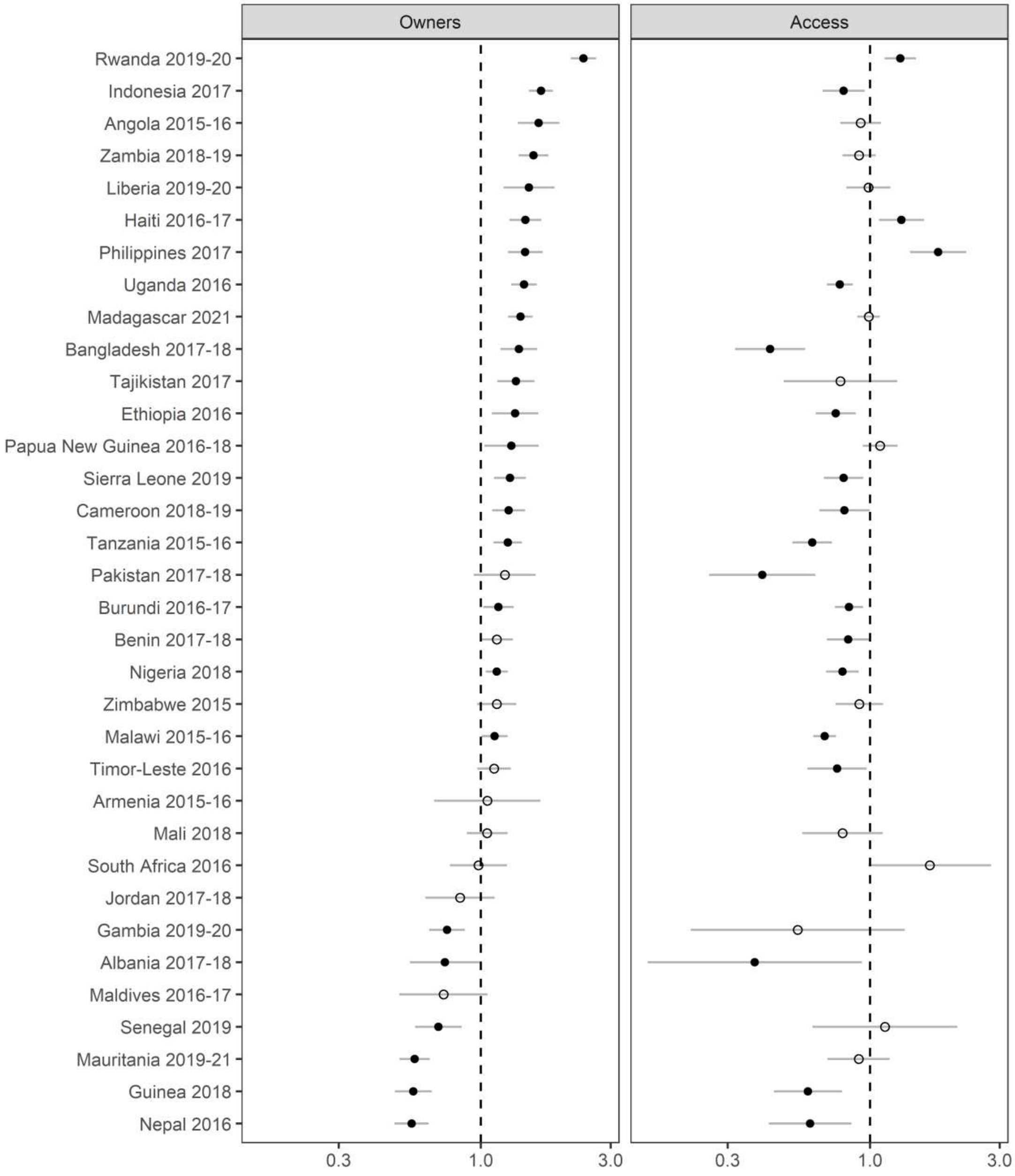

Note: Estimates for access to a mobile phone cannot be computed for Armenia, Jordan, and Maldives due to small sample size. Fill dots mean that estimates are statistically significant at the 95% confidence level, while empty dots mean that estimates are not statistically significant.

Appendix Figure 6: Under-five mortality rate and total fertility rate estimates testing for post-stratification weights from women who have access to a mobile phone.

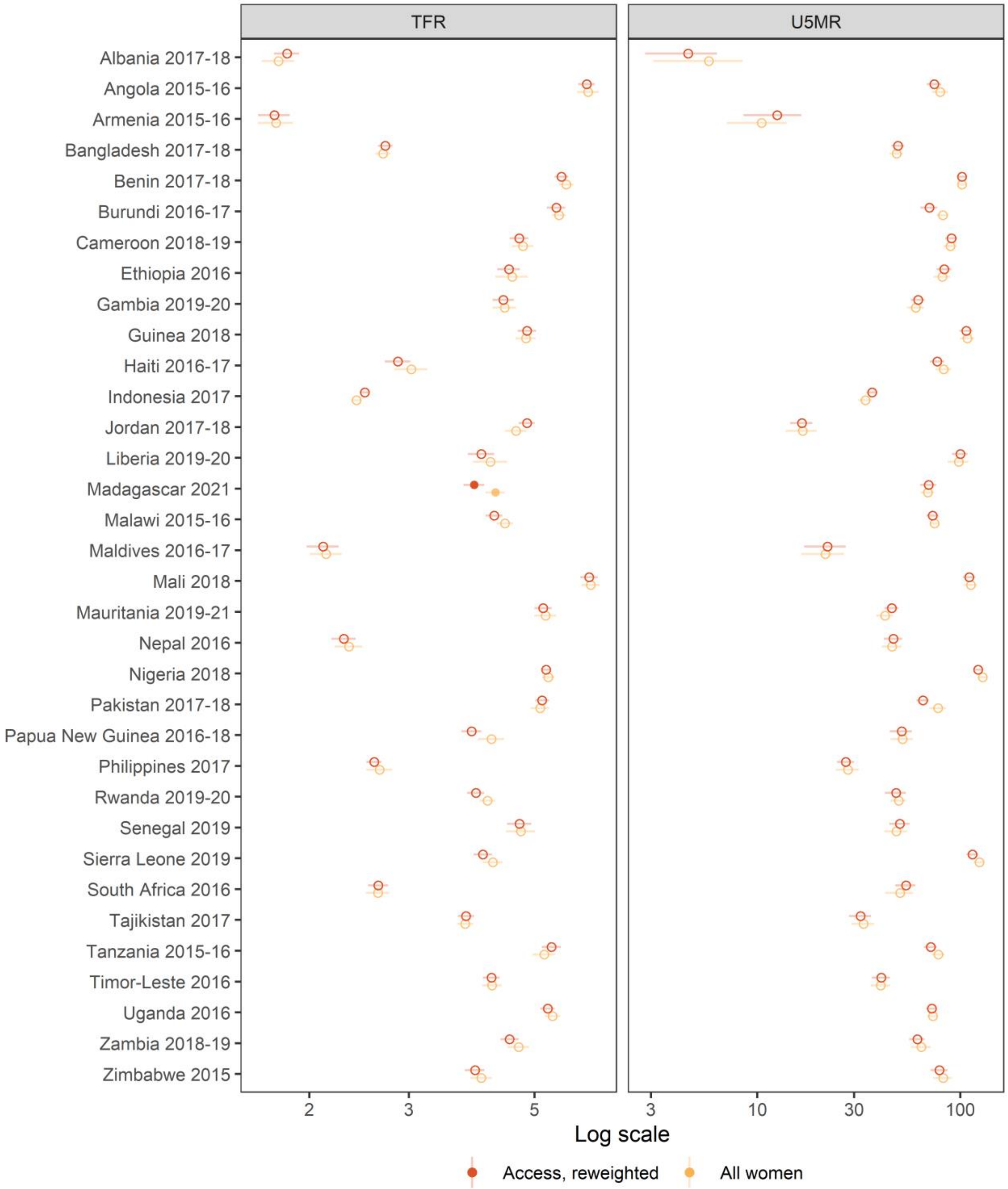

Note: Fill dots mean that the difference between the actual rate and the re-weighted rate is statistically significant at the 95% confidence level, while empty dots mean that the difference is not statistically significant.

Appendix Figure 7: Estimates of the difference in current use of modern contraceptives (MCU), sexual activity (SexAct), duration of postpartum amenorrhea (Amen), and duration of postpartum abstinence (Abst), between having and not having access to a mobile phone.

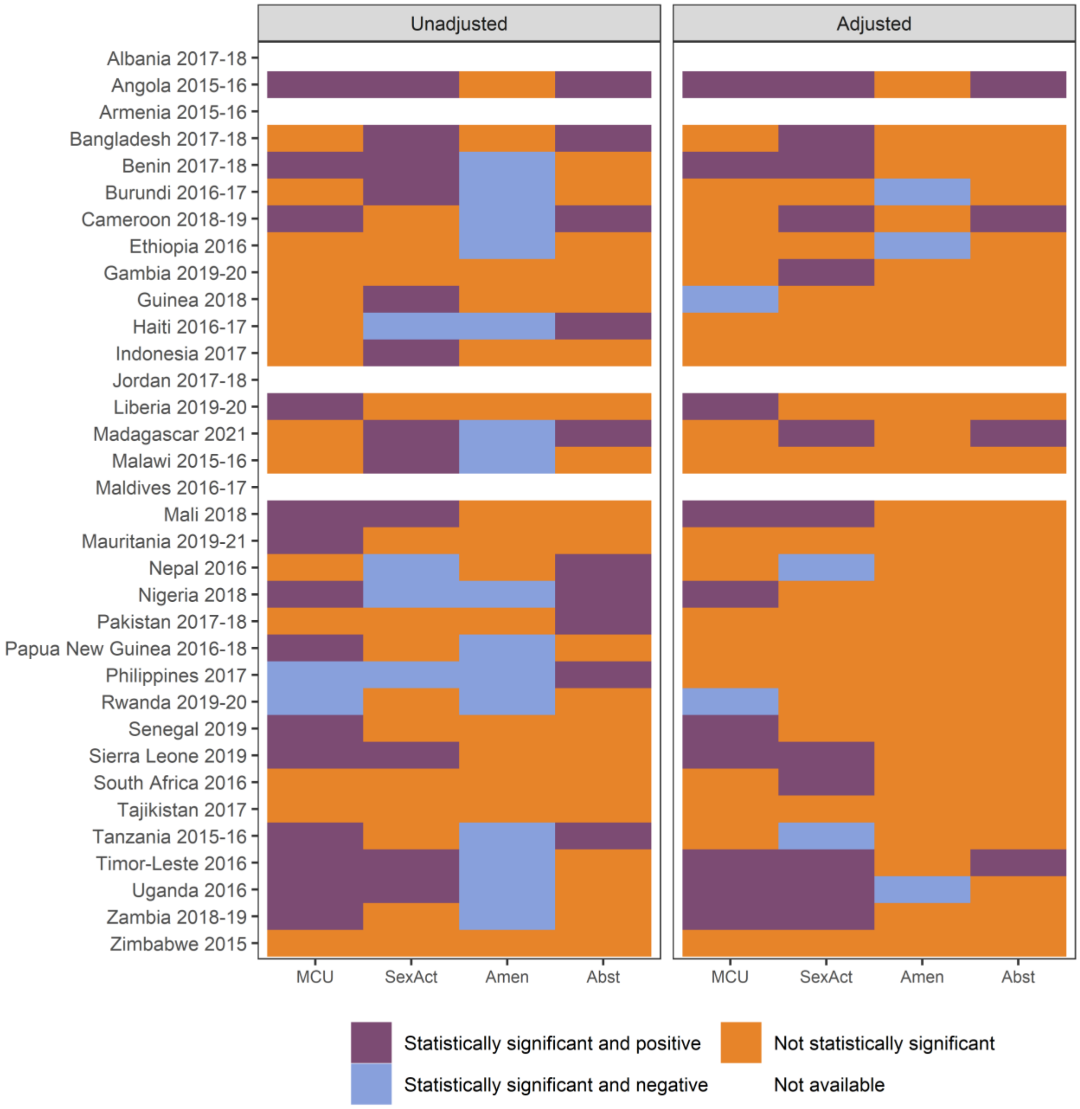

Appendix Figure 8: Estimates of the difference in exclusive breastfeeding (BrFeed), births who had at least 4 antenatal care visits (ANC), newborns who received at least 2 signal functions (PNC), children who received basic immunization (Vax), births delivered by skilled professionals (Skill), children who are underweight (UnderW), between having and not having access to a mobile phone.

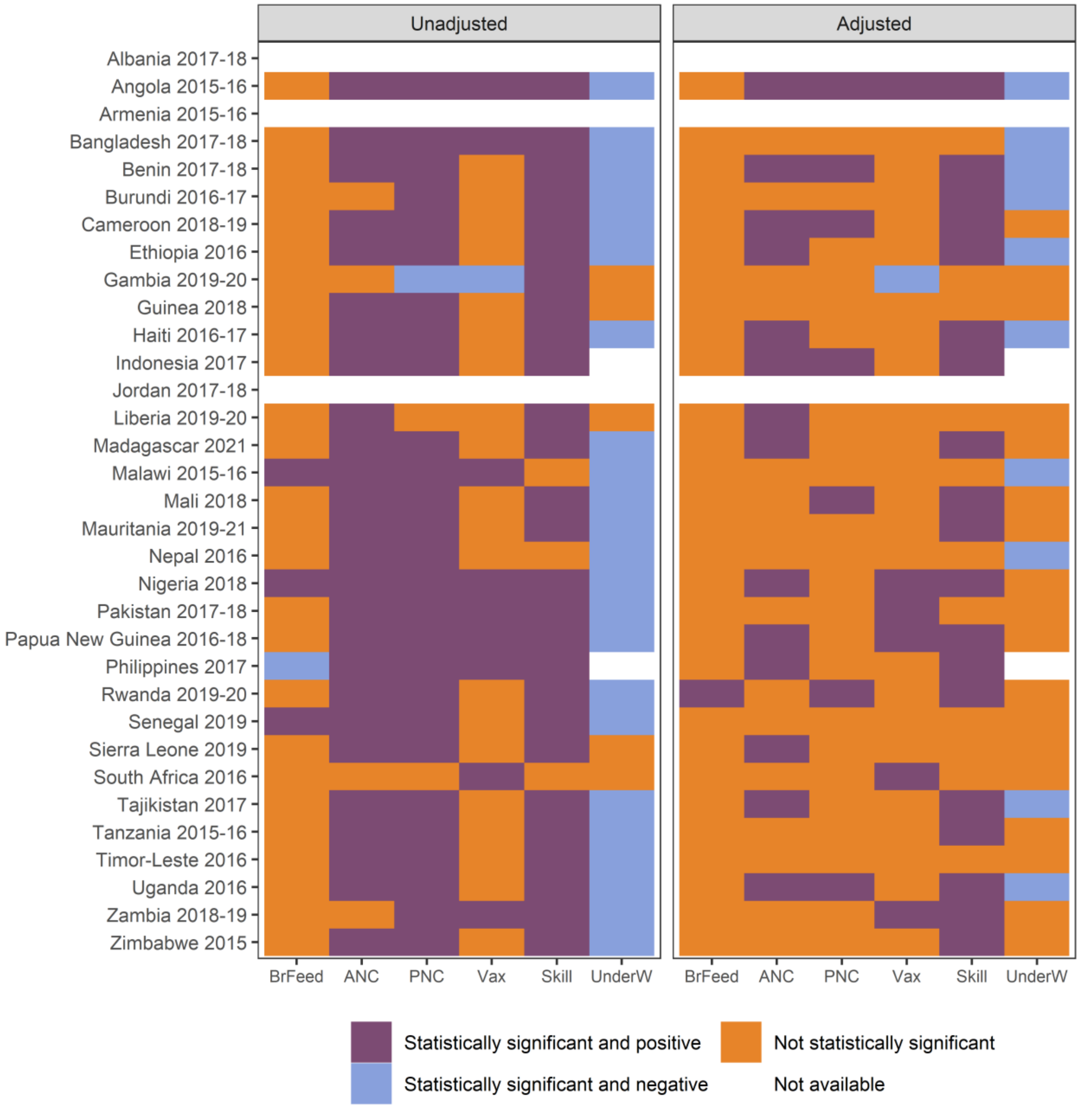

Supplement: Supplementary data [file bmjopen-2023-071791supp001.pdf]
