# Supplementary material for: Measuring under-5 mortality and fertility through mobile phone surveys: an assessment of selection bias in 34 low-income and middle-income countries
Source: BMJ Open. 2023 Nov 17;13(11):e071791. doi: 10.1136/bmjopen-2023-071791 (PMC10693685; doi:10.1136/bmjopen-2023-071791)
Supplement: Author's manuscript [file bmjopen-2023-071791.draft_revisions.pdf]

BMJ Open is committed to open peer review. As part of this commitment we make the peer review history of every article we publish publicly available.

When an article is published we post the peer reviewers' comments and the authors' responses online. We also post the versions of the paper that were used during peer review. These are the versions that the peer review comments apply to.

The versions of the paper that follow are the versions that were submitted during the peer review process. They are not the versions of record or the final published versions. They should not be cited or distributed as the published version of this manuscript.

BMJ Open is an open access journal and the full, final, typeset and author-corrected version of record of the manuscript is available on our site with no access controls, subscription charges or pay-per-view fees (http://bmjopen.bmj.com).

If you have any questions on BMJ Open's open peer review process please email info.bmjopen@bmj.com

Measuring under-five mortality and fertility through mobile phone surveys: an assessment of selection bias in 34 low- and middle-income countries

Journal:	*BMJ Open*
Manuscript ID	bmjopen-2023-071791
Article Type:	Original research
Date Submitted by the Author:	12-Jan-2023
Complete List of Authors:	Sánchez-Páez, David; Université catholique de Louvain, Centre for Demographic Research<br>Masquelier, Bruno; Université catholique de Louvain, Centre for Demographic Research<br>Menashe-Oren, Ashira; Université catholique de Louvain, Centre de Recherché en Démographie<br>Baruwa , Ololade Julius ; Université catholique de Louvain, Centre for Demographic Research<br>Reniers, George; London School of Hygiene and Tropical Medicine, Population Studies Group
Keywords:	Demography < TROPICAL MEDICINE, STATISTICS & RESEARCH METHODS, Epidemiology < TROPICAL MEDICINE

SCHOLARONE™
Manuscripts

I, the Submitting Author has the right to grant and does grant on behalf of all authors of the Work (as defined in the below author licence), an exclusive licence and/or a non-exclusive licence for contributions from authors who are: i) UK Crown employees; ii) where BMJ has agreed a CC-BY licence shall apply, and/or iii) in accordance with the terms applicable for US Federal Government officers or employees acting as part of their official duties; on a worldwide, perpetual, irrevocable, royalty-free basis to BMJ Publishing Group Ltd ("BMJ") its licensees and where the relevant Journal is co-owned by BMJ to the co-owners of the Journal, to publish the Work in this journal and any other BMJ products and to exploit all rights, as set out in our licence.

The Submitting Author accepts and understands that any supply made under these terms is made by BMJ to the Submitting Author unless you are acting as an employee on behalf of your employer or a postgraduate student of an affiliated institution which is paying any applicable article publishing charge ("APC") for Open Access articles. Where the Submitting Author wishes to make the Work available on an Open Access basis (and intends to pay the relevant APC), the terms of reuse of such Open Access shall be governed by a Creative Commons licence – details of these licences and which Creative Commons licence will apply to this Work are set out in our licence referred to above.

Other than as permitted in any relevant BMJ Author's Self Archiving Policies, I confirm this Work has not been accepted for publication elsewhere, is not being considered for publication elsewhere and does not duplicate material already published. I confirm all authors consent to publication of this Work and authorise the granting of this licence.

For peer review only

Measuring under-five mortality and fertility through mobile phone surveys: an assessment of selection bias in 34 low- and middle-income countries

AUTHORS:

1. David A. Sánchez-Páez (Corresponding author).
   Affiliation: Centre for Demographic Research, Université catholique de Louvain.
   Address: Place Montesquieu 1/L2.08.03
   1348 Louvain-la-Neuve
   Belgium.
   E-mail: david.sanchezpaez@uclouvain.be
   ORCID: 0000-0002-7828-8193

2. Bruno Masquelier.
   Affiliation: Centre for Demographic Research, Université catholique de Louvain.
   Address: Louvain-la-Neuve, Belgium.
   ORCID: 0000-0002-9585-891X

3. Ashira Menashe-Oren.
   Affiliation: Centre for Demographic Research, Université catholique de Louvain.
   Address: Louvain-la-Neuve, Belgium.
   ORCID: 0000-0002-4781-5384

4. Ololade Julius Baruwa.
   Affiliation: Centre for Demographic Research, Université catholique de Louvain.
   Address: Louvain-la-Neuve, Belgium.
   ORCID: 0000-0002-0776-528X

5. Georges Reniers.
   Affiliation: Population Studies Group, London School of Hygiene & Tropical Medicine.
   Address: London, United Kingdom.
   ORCID: 0000-0001-6582-1692

Keywords: Under-five mortality; fertility; mobile phone surveys; low- and middle-income countries; selection bias; Demographic and Health Surveys; mobile phone ownership; mobile phone access.

Word count: 3,890 exclusive of title page, abstract, summary box, references, figures, tables, and declaration statements.

For peer review only

ABSTRACT

Introduction: In countries lacking comprehensive systems of civil registration and vital statistics, the collection of demographic data through mobile surveys is increasingly feasible, as mobile phone coverage has rapidly expanded. However, mobile phone users are often younger, male, with higher education, and wealthier, potentially biasing demographic estimates based on phone surveys.

Methods: We assess selection bias in estimates of under-five mortality and fertility using data from 34 Demographic and Health Surveys. We use logistic regressions to characterize individuals who own or have access to a mobile phone in terms of sociodemographic background characteristics, and Poisson regressions to estimate the association between mobile phone ownership (or access) and total fertility and under-five mortality rates. We further evaluate the potential reasons why estimates by mobile phone ownership may differ using a set of behavioral characteristics.

Results: Fertility and mortality rates would be biased downwards if estimates are only based on women who own or have access to mobile phones. Under-five mortality estimates can be adjusted through post-stratification using age, educational level, area of residence, wealth, and marital status as weights. However, fertility estimates remain biased even after adjusting for these covariates. This difference is associated with behavioral factors that are not captured by the post-stratification variables, but for which there are also differences between mobile phone owners and non-owners. In many countries, modern contraceptive use is positively associated with mobile phone ownership, which may complicate fertility estimates based on mobile phone surveys.

Conclusions: Mobile phone surveys need to include background questions on socioeconomic status to be able to weight and adjust mortality estimates ex post-facto. Fertility estimates from mobile phone surveys will be biased unless further research uncovers the mechanisms driving the bias.

For peer review only

What is already known on this topic:

- Mobile phone ownership and access is associated with socioeconomic status, which could bias demographic estimates derived from data collected through mobile phone surveys.
- In empirical studies analyzing selection bias in estimates derived from mobile phone surveys we find isolated evidence from individual countries suggesting selection bias in fertility and mortality estimates.

What this study adds:

- We established that fertility and under-five mortality estimates derived from birth histories administered for women who own or have access to a mobile phone will be biased downwards.
- Post-stratification on sociodemographic background characteristics corrects the bias in mortality estimates, but adjusted fertility estimates remain lower than in a nationally representative sample, including women who do not own or have access to a mobile phone. This may be due to the fact that fertility decision-making is volitional and not fully captured by women's background and living conditions.

How this study might affect research, practice or policy:

- Mobile phone surveys in low- and middle-income countries are likely to become increasingly common, and they can be useful for generating nationally representative estimates of demographic and health indicators. As long as mobile phone ownership is unequally distributed, however, it will remain important to also collect data that can be used in post-stratification procedures.

For peer review only

INTRODUCTION

Use of mobile phones has spread widely across the world, including in low- and middle-income countries (LMIC) where the technological infrastructure for landlines is often lacking[1,2]. This spread has allowed populations to communicate faster and effortlessly, to access internet, and it has accelerated social and economic development[3,4]. Widespread and increasing coverage of mobile phones also provides an opportunity to conduct mobile phone surveys (MPS). Some benefits of MPS include cost efficiency, shorter fieldwork duration due to reduced time devoted to travel, improved ability to supervise data collection through the recording of interviews and automated storage of paradata, and increased access to populations that are harder to reach[5,6]. MPS have already covered some health-related topics, including family planning and sexual and reproductive health[1,7], but few MPS have included questions to monitor trends in fertility and mortality.

In countries lacking comprehensive systems of civil registration and vital statistics, data on mortality and fertility are generally collected through face-to-face interviews in survey programs such as the Demographic and Health Surveys (DHS) or Multiple Indicator Cluster Surveys (MICS). However, face-to-face interviews might not be the most cost-effective approach and they are not suitable in settings affected by conflicts, disasters or epidemic outbreaks. During the Ebola epidemic in Liberia, MPS were used to estimate mortality both from Ebola and other causes based on reports of recent household deaths[8]. During the COVID-19 pandemic, MPS were proposed as a viable way to assess excess mortality without posing additional risks to survey teams and respondents[9,10]. MPS thus represent a promising alternative to collect demographic data.

For peer review only

MPS have several disadvantages that require careful consideration, such as higher non-response rates and shortened interview duration – requiring changes to the questionnaires used in face-to-face interviews[11]. Moreover, MPS may not be representative, especially when based on random digit dialing, as compared to a survey using phone numbers previously collected in face-to-face surveys[1]. This is because inequalities in use of digital technology, known as a “digital divide”, persist. Mobile phone use is related to gender, socioeconomic status, technological proficiency and literacy. Women have been noted to be at a disadvantage in mobile phone use, although the more common mobile phones are, the smaller the gender gap is[12–14]. This gender divide in mobile phone use reflects multiple barriers women face, including lack of financial resources, lack of time due to care responsibilities or household chores, socio-cultural restrictions, and lower educational attainment[15]. Mobile phone users are typically also better educated[16] and younger[17]. Moreover, rural dwellers use mobile phones relatively less than urbanites[18], although overall access remains high in rural areas as well[19]. Other factors, not related directly to an individual’s characteristics, also determine selectivity in mobile phone users. For example, in remote locations and near borders, cellular network coverage may be weak, leaving populations living in such areas under-represented in MPS.

At the aggregate level, use of mobile phone has been linked to sustainable development[2]. The higher the diffusion of mobile phones, the lower under-five (U5M) and maternal mortality, the greater the use of contraception, and the smaller ideal family size[2,20]. This is in part because mobile phone use is a good proxy for socioeconomic development, but also because mobile phone use can improve access to health services, including reproductive health and antenatal care[21–25]. Such benefits could hinder obtaining a representative sample, even after post-sampling weighting. For example, a study conducted in Burkina Faso found higher modern contraception use among

For peer review only

respondents reached by an MPS (40%) compared to respondents in a face-to-face survey (26%)[7]. This difference in modern contraceptive use persisted after post-stratification. Similarly, Lefevre and colleagues[26] explored variations in several reproductive maternal, newborn and child health indicators, such as antenatal and postnatal care, skilled attendance at birth, measles and tetanus vaccinations, using DHS surveys in 15 countries. Even after controlling for a wide range of sociodemographic variables, child health and natal care was better when mothers had a mobile phone. A notable exception was the adoption of initial, exclusive, and continuous breastfeeding, which was less common among mobile phone users.

These previous studies suggest that fertility and mortality estimates derived from MPS are likely to be biased, but this issue has not been systematically evaluated. In this study, we leverage existing DHS data containing information on mobile phone use by women to assess the selection bias in U5M and fertility estimates. We expect that these estimates are biased downwards, since mortality and fertility tend to be higher in lower socioeconomic groups[27,28]. We assess mobile phone use by distinguishing between women owning a mobile phone (henceforth referred to as 'ownership') and those who can potentially be reached through another household member who owns a phone (henceforth referred to as 'access'). We first identify correlates of mobile phone ownership and access through logistic regressions and establish through Poisson regressions whether ownership and access are associated with variations in U5M and fertility.

DATA AND METHODS

Data collection

We used all available DHS[29] that include information on mobile phone ownership and access. The question on mobile phone *ownership* (v169a) is included in the individual questionnaire, while the

For peer review only

question on *access* to a mobile phone (hv243a) is in the household questionnaire. Access to mobile phone means that the woman either owns a mobile phone or lives in a household where the household head owns a mobile phone. Some countries have more than one survey with such data, so in these cases, we used the most recent one. In total, we used 34 DHS, mostly from Sub-Saharan Africa (see Appendix Table 1 in the supplementary material). These surveys were collected between 2015 and 2021. All women have complete information for all variables of interest included in this study.

The survey India 2019-21 is excluded from the analysis as the information is missing for about 85% of respondents. In Papua New Guinea 2016-18 information is missing for 0.4% of respondents, in Indonesia 2017 for 0.1%, and in Pakistan 2017-18 for 0.01%. We removed the observations with missing data from these three samples.

Data analysis

Using data from the birth histories administered for women aged 15–49, we computed total fertility rates (TFR) for the 36-month period preceding the survey and under-five mortality rates (U5MR) for the 10-year period preceding the survey. We used Poisson regressions to estimate the association between mobile phone ownership and access and fertility and U5M rates. For fertility, the outcome variable was the number of births, while for U5M, it was the number of deaths before age 5. We conducted two sets of regressions. In the first set — unadjusted estimates —, we regressed the outcome variable on the ownership of a mobile phone (model 1) or access to a mobile phone in the household (model 2), including covariates for the mother's age group when estimating fertility, and the child's age when estimating mortality. In the second set — adjusted estimates — , we added educational level, place of residence, wealth, and marital status as independent variables

For peer review only

to models 1 and 2. As for the categories of these independent variables, place of residence has two values: urban and rural; educational level takes three values: no education, primary education, and secondary and higher education; wealth has two values: poor (wealth quintiles 1 and 2) and not poor (wealth quintiles 3, 4 and 5); and, marital status takes two values: not in union (never married, not living together, divorced, or widowed) and in union (married or living together). We also included exposure (person-years calculated for women for fertility and for children for mortality) as an offset in all models. In addition, we computed raking weights to match the marginal distributions of the same sociodemographic variables and examine whether potential estimation biases could be addressed using post-stratification.

Differences in U5MR and TFR could be due to differences in women's behavior, which could be captured by mobile phone use, but not by sociodemographic variables. We proposed a series of logistic regressions to analyze the association between mobile phone ownership and access, and some variables connected to U5M and fertility. For mortality, we used six outcome variables. First, we modeled the likelihood of a mother having at least 4 antenatal care visits among those who gave birth in the 5 years before each survey. Second, the likelihood of a newborn receiving at least two signal functions as part of postnatal care (among births that occurred in the last 24 months). Third, the likelihood of a child being exclusively breastfed for the first 6 months (among the most recent birth in the last 6 months). Fourth, the likelihood of a child receiving eight basic immunizations among children aged 12 to 35 months at the time of the survey. The eight basic immunizations are one dose of BCG, three doses of DPT, three doses of Polio (excluding at birth), and one dose of Measles. Fifth, we analyzed the likelihood of skilled attendance at birth, which includes the presence of a doctor, nurse or nurse-midwife during delivery. All births in the 5-year period before the survey are considered. The last outcome variable connected with U5M that was analyzed was

For peer review only

the prevalence of underweight among children aged 0–5 at the time of the survey. A child is considered as being underweight if the weight-for-age z-score is below two standard deviations below the mean on the WHO Child Growth Standards. On the other hand, for fertility we used four outcome variables. First, the use of modern contraceptives. Second, sexual activity. We considered a woman to be sexually active when she reported having had sexual intercourse in the last month or being pregnant. Lastly, we analyzed duration of postpartum amenorrhea and duration of postpartum abstinence. For the latter two variables, we used a linear model since duration is measured as the number of months. Similar to the estimates of under-five fertility and mortality rates, unadjusted and adjusted models were estimated for all outcome variables.

We conducted this analysis using the programming language for statistical computing and graphics `R`[30]. We used the following packages: `rdhs`[31] for downloading the DHS datasets, `tidyverse`[32] for data manipulation and graphics, `demogsurv`[33] for preparing person-period tables stratified by the independent variables, `survey`[34] and `broom`[35] for econometric estimations and visualization, `anesrake` for computing raking weights to match the marginal distributions of sociodemographic variables for testing for post-stratification, `MetBrewer`[36] for color palettes in our plots, and `kableExtra`[37] and `knitr`[38] for writing and editing the manuscript.

Patient and public involvement

Patients and/or the public were not involved in the design, or conduct, or reporting, or dissemination plans of this research.

For peer review only

RESULTS

Across the 34 surveys used, the percentage of women owning a mobile phone ranges from 23.6% in Burundi to 96.7% in Armenia. The percentage of women who have access to a mobile phone ranges from 55% in Burundi to 100% in the Maldives (see Appendix Table 1). The median ratio of the TFR calculated among non-owners to the TFR calculated among mobile phone owners is 1.48. Fertility is always higher among non-owners, with the lowest ratio at 1.04 in Bangladesh and the highest at 1.93 in Haiti. In absolute terms, these differences correspond to more than one child per woman in 25 of the 34 surveys studied here. Similarly, U5M is higher when calculated among non-owners, with the exception of South Africa 2016 and Zambia 2018-19. The median ratio of U5M calculated from reports of non-owners to that calculated from mothers with mobile phones is 1.29, and it ranges from 0.85 in South Africa to 2.5 in Albania. Differences in TFR and U5MR remain when access to a mobile phone is considered, but they are slightly reduced.

A series of logistic regressions (see Appendix Figures 1 to 5 in the supplementary material) conducted for each survey separately indicate that women with mobile phones differ from non-owners by age group, educational level, area of residence, wealth quintile, and marital status. Except for age, differences do not disappear when access to a mobile phone instead of ownership is considered. Compared to young adults aged 20–24, adolescents aged 15–19 are less likely to own a phone in all countries, although this is not true in 27 countries when we consider access rather than ownership. Age patterns of phone ownership vary by country for older women, but in most cases, differences are not statistically significant when access is considered.

Compared to women without education, women with primary education are significantly more likely to own a mobile phone in 30 surveys and women with secondary or higher education are

For peer review only

significantly more likely to own a mobile phone in all surveys. The number of surveys with a significant difference decreases, respectively, to 28 surveys and 30 surveys when access is considered. Women living in urban areas are significantly more likely to own a mobile phone in 29 surveys and to have access in 19 surveys. Odds ratios for women who own a mobile phone range from 0.94 (95% CI: 0.74 – 1.19) in South Africa to 5.42 (95% CI: 4.23 – 6.95) in Ethiopia, while for access, from 0.41 (95% CI: 0.25 – 0.67) in South Africa to 4.2 (95% CI: 3.12 – 5.67) in Sierra Leone.

In terms of household assets, the gaps are very large between women in the poorest households (quintiles 1 to 2) and those living in the most affluent households (quintiles 3 to 5). Unsurprisingly, women living in households with more amenities are between 1.17 (Maldives, 95% CI: 0.83 – 1.66) times and 4.85 (Burundi, 95% CI: 4.24 – 5.55) times more likely to own a mobile phone. As for access, the odds ratios range between 2.7 (Mali, 95% CI: 2.03 – 3.58) and 24.37 (Indonesia, 95% CI: 18.01 – 32.97). Women not in union are more likely to own a mobile phone than women in union in 19 countries, with odds ratios ranging from 0.56 (95% CI: 0.48 – 0.64) in Nepal to 1.78 (95% CI: 1.4 – 2.26) in the Philippines.

Figures 1 and 2 present the exponentiated coefficients of mobile phone ownership and access from Poisson regressions on U5M and fertility, respectively. Left panels of both figures show unadjusted estimates, while right panels contain adjusted estimates. Unadjusted coefficients suggest that in most countries, ownership is associated with lower odds of experiencing a birth or the death of a child under five years of age. When considering U5M estimates and mobile phone ownership, coefficients are statistically significant in all countries but nine (left panel of Figure 1). However, in most countries differences by ownership are no longer significant once we adjust for marital status, education, wealth, and place of residence (right panel of Figure 1). Women who own a

For peer review only

mobile phone are less likely to lose a child in only four countries (Cameroon, Gambia, Nepal, and Nigeria) after adjusting for sociodemographic characteristics. Overall, U5M is not statistically associated with owning or having access to a mobile phone, after controlling for basic sociodemographic characteristics. U5MR estimates obtained using post-stratification weights from mobile phone owners are in line with these findings (see right panel of Figure 3). Only in Nigeria is it not possible to reproduce actual U5MR using ex-post weightings. Results for access are presented in Appendix Figure 6.

Unadjusted coefficients from the Poisson regressions on fertility for mobile phone owners are also statistically significant in 30 countries (left panel of Figure 2). However, in contrast to what we observed for U5M, the negative effect of owning a phone persists for fertility in most countries even after controlling for covariates (right panel of Figure 2). Ethiopia is the only survey where this association loses significance after controlling for sociodemographic characteristics. When access is considered, the effect is not significant in five additional countries (Burundi, Cameroon, Mali, Pakistan, and Senegal). In most countries, ownership or access is associated with lower fertility, even after controlling for basic sociodemographic characteristics. The use of post-stratification weights based on the marginal distribution of sociodemographic variables of mobile phone owners shows that it is possible to reproduce the observed TFR in a total of 22 countries (see left panel of Figure 3). However, this also means that post-stratification cannot provide unbiased fertility estimates in one-third of the countries included in our study. When access is considered (see Appendix Figure 6), there are no statistical differences with the TFRs observed in the general population. As mobile phone access is very high, marginal distributions of the sociodemographic variables of women with access are very similar to that of the whole population so post-stratification plays a small role in this case.

For peer review only

In Figure 4, we present the estimates of the association between ownership and variables connected to fertility. Results for access to mobile phones are presented in Appendix Figure 7. We observe statistically significant differences in use of modern contraceptives in 23 surveys. In most of them, owners are more likely to use contraceptives. After controlling for covariates, differences remain in 20 surveys. When access is considered, the number of surveys is reduced to 16. In contrast, mobile phone owners are less likely to be sexually active in 14 surveys, even after controlling for sociodemographic characteristics. However, differences disappear in most surveys when access is considered. Postpartum amenorrhea periods are significantly shorter among owners in 19 surveys, although only in 8 surveys after including controls. In contrast, owners have significantly longer postpartum abstinence periods in 15 surveys, but only in 8 surveys after adjusting for controls.

Analyses of the association between ownership and variables connected to U5M are presented in Figure 5. Results for access are in Appendix Figure 8. Owners are more likely to have had at least four antenatal care visits in all but 3 surveys. Differences are not statistically significant in 6 surveys after controlling for covariates. Likewise, births born to mothers who own a mobile phone are more likely to have received at least two signal functions as part of postnatal check-ups in 28 surveys, although the number of surveys is reduced to 18 after controlling for covariates. In contrast, owners are more likely to exclusively breastfeed for the first 6 months to newborns in 4 surveys, and less likely in 4 other surveys, although these differences disappear in virtually all surveys after controlling for covariates. Children born to owners are more likely to have received basic immunization in only 5 surveys after adjusting for controls. In contrast, children born to owners are more likely to have been attended by health professionals at delivery in 27 surveys. Owners are less likely to have underweight children in more than half of the surveys, even after controlling for

For peer review only

covariates. Differences disappear for most variables when access is considered. Exceptions are antenatal care, the presence of a health professional at delivery, and, to a lesser extent, underweight.

CONCLUSIONS

We analyzed the social gradient in mobile phone ownership and access in 34 LMIC, and evaluated the implications for fertility and U5M estimates derived from MPS. Consistent with previous research[16–19], we found that well educated, better-off, and urban women are more likely to own and have access to a mobile phone. These differences in the use of mobile phones could bias demographic estimates from MPS.

Fertility and U5M are higher in less advantaged populations[27,28], groups which are underrepresented in MPS. Therefore, MPS are likely to underestimate true levels of fertility and U5M in the wider population. This is an important first conclusion of this study: MPS will underestimate U5M and total fertility. However, the negative association between U5M and mobile phone ownership disappears once we control for a number of sociodemographic characteristics that can be easily collected in MPS. This is a second, and methodologically, important finding of our study as it suggests that any downward bias in U5M estimates from MPS due to selectivity of respondents can be corrected using a post-stratification procedure. More substantively, our results contradict the hypothesis that at the aggregate level the expansion of mobile phones is associated with lower child mortality as this association seems spurious[2].

Surprisingly, the negative association between mobile phone ownership and fertility persists in many of the countries included in this study even after controlling for sociodemographic characteristics. From a methodological standpoint, this suggest that fertility rates could be biased downwards if they are estimated from MPS, even after post-stratification. This third important

For peer review only

finding is consistent with previous research arguing that mobile phone use is associated with higher rates of contraceptive use[2,7,20]. Our results suggest that the use of modern contraceptives is more likely among owners in two out of five surveys analyzed in this study, even after controlling for wealth, education, place of residence, marital status and age. Further, there are some apps that track menstrual periods and send alerts on ovulation days, allowing for better birth control[2,20]. Even less sophisticated models of mobile phones — i.e., not smartphones — include calendars that can be used to schedule reminders for tracking the menstrual cycle or taking contraceptives. In addition, we found that mobile phone owners are less likely to be sexually active in most of the surveys. Higher odds of contraceptive use and lower odds of sexual activity lead to lower fertility rates. It is worth noting that one difference between fertility and mortality is that fertility outcomes can be more easily controlled based on preferences than exposure to illness and injuries. These fertility preferences may be correlated with, but are not fully captured by, the sociodemographic characteristics.

Our results also show that there are no significant differences between owners and non-owners in terms of exclusive breastfeeding. This does not contradict what Lefevre and colleagues[26] found, as they estimated their results using differences in means without controlling for covariates while we have used the association of exclusive breastfeeding conditional on mobile phone ownership and other sociodemographic variables. Our findings can help to explain why the differences in U5MR by ownership disappear when controlling for covariates, since the health benefits from exclusive breastfeeding and basic immunization could be offsetting the negative effects of no or few prenatal and postnatal checkups, or low birth weight.

As for logistical implications for fieldwork, in some countries, MPS should be conducted at times when the household head is present so that women in the household who do not own a mobile

For peer review only

phone can be surveyed. In other countries, biases related to the selection of phone users may remain even after using this approach. A different way of accounting for selection bias (apart from post-stratification), is to use sample quotas, that is pre-stratification. This could be based on a set of simple screening questions like age, rural/urban residence and educational attainment. However, pre-stratification might be more expensive and it will require greater effort to reach respondents with the desired attributes, while post-stratification will come at the cost of precision. It should be noted that our study evaluates selection bias due to sampling frame limitations (mobile phone access/ownership) and not due to non-response. Non-response is often high in MPS, and considerably higher than in face-to-face surveys. We did not consider either the recall errors which could be more systematic in MPS due to lower engagement of the respondents.

In conclusion, while MPS are a cheaper alternative or complement to classical face-to-face surveys for estimating demographic and health indicators, estimates derived from MPS cannot be taken at face value owing to the unequal ownership and access of mobile phones in many LMIC. In this study, we have shown that under-five mortality estimates can be corrected using a post-stratification on a number of sociodemographic background characteristics to match nationally representative estimates. Adjusting fertility estimates is less straightforward, most likely because fertility decision making is an expression of individual preferences in addition to background characteristics and living conditions.

Contributors

DASP and BM led the conception and design of the study. DASP and BM analyzed the data, produced the estimates and drafted the manuscript. AMO, JB and GR reviewed the estimates and helped with the interpretation of the results. All authors critically reviewed the manuscript and

For peer review only

approved the final version of the paper. DASP is the guarantor of the overall content of this manuscript and had final responsibility for the decision to submit for publication.

Competing interests

We declare no competing interests.

Funding

This study was supported by the Bill & Melinda Gates Foundation (INV-023211). The funders of the study had no role in the study design, data analysis, data interpretation, or writing of the report.

Data availability statement

All datasets are publicly and freely available on the DHS Program website. https://dhsprogram.com/data/

Patient and public involvement

Patients and/or the public were not involved in the design, or conduct, or reporting, or dissemination plans of this research.

Patient consent for publication

Not applicable.

Ethics Approval

Not applicable since the study was a secondary analysis of publicly available data.

For peer review only

References

1 Gibson DG, Pereira A, Farrenkopf BA, Labrique AB, Pariyo GW, Hyder AA. Mobile phone surveys for collecting population-level estimates in low-and middle-income countries: A literature review. *Journal of Medical Internet Research* 2017; **19**: e139.

2 Rotondi V, Kashyap R, Pesando LM, Spinelli S, Billari FC. Leveraging mobile phones to attain sustainable development. *Proceedings of the National Academy of Sciences* 2020; **117**: 13413–20.

3 Donner J. The use of mobile phones by microentrepreneurs in Kigali, Rwanda: Changes to social and business networks. *Information Technologies and International Development* 2007; **3**: 3–19.

4 Jagun A, Heeks R, Whalley J. The impact of mobile telephony on developing country micro-enterprise: A Nigerian case study. *Information Technologies and International Development* 2008; **4**: 47–65.

5 Firchow P, Ginty RM. Including hard-to-access populations using mobile phone surveys and participatory indicators. *Sociological Methods & Research* 2020; **49**: 133–60.

6 Kempf AM, Remington PL. New challenges for telephone survey research in the twenty-first century. *Annual Review of Public Health* 2007; **28**: 113–26.

7 Greenleaf AR, Gadiaga A, Guiella G, *et al.* Comparability of modern contraceptive use estimates between a face-to-face survey and a cellphone survey among women in Burkina Faso. *PLOS ONE* 2020; **15**: e0231819.

8 Kuehne A, Lynch E, Marshall E, *et al.* Mortality, morbidity and health-seeking behaviour during the Ebola epidemic 2014–2015 in Monrovia results from a mobile phone survey. *PLoS Neglected Tropical Diseases* 2016; **10**: 1–2.

9 Adjiwanou V, Alam N, Alkema L, *et al.* Measuring excess mortality during the COVID-19 pandemic in low- and lower-middle income countries: The need for mobile phone surveys. 2020. DOI:10.31235/osf.io/4bu3q.

10 Chasukwa M, Choko AT, Muthema F, *et al.* Collecting mortality data via mobile phone surveys: A non-inferiority randomized trial in Malawi. *PLOS Global Public Health* 2022; **2**: e0000852.

11 Dabalen A, Etang A, Hoogeveen J, Mushi E, Schipper Y, Engelhardt J von. Mobile phone panel surveys in developing countries: A practical guide for microdata collection. Washington, DC: World Bank, 2016.

12 Blumenstock JE, Hall MG, Eagle N. Divided we call: Disparities in access and use of mobile phones in Rwanda. *Information Technologies and International Development* 2012; **8**: 1–6.

For peer review only

13 Huyer S, Hafkin N, Ertl H, Dryburgh H. Women in the Information Society. In: The digital divide to digital opportunities: Measuring infostates for development. NRC Press, 2005: 135–96.

14 Murphy LL, Priebe AE. "My co-wife can borrow my mobile phone!": Gendered geographies of cell phone usage and significance for rural Kenyans. *Gender, Technology and Development* 2011; **15**: 1–23.

15 Huyer S, Carr M. Information and Communication Technologies: A priority for women. *Gender, Technology and Development* 2002; **6**: 85–100.

16 L'Engle K, Sefa E, Adimazoya EA, *et al.* Survey research with a random digit dial national mobile phone sample in Ghana: Methods and sample quality. *PLoS ONE* 2018; **13**: e0190902.

17 Aker JC, Mbiti IM. Mobile phones and economic development in Africa. *Journal of Economic Perspectives* 2010; **24**: 207–32.

18 Wesolowski A, Eagle N, Noor AM, Snow RW, Buckee CO. Heterogeneous mobile phone ownership and usage patterns in Kenya. *PLos ONE* 2012; **7**: e35319.

19 Wakadha H, Chandir S, Victor E, *et al.* The feasibility of using mobile-phone based SMS reminders and conditional cash transfers to improve timely immunization in rural Kenya. *Vaccine* 2013; **31**: 987–93.

20 Billari F, Rotondi V, Trinitapoli J. Mobile phones, digital inequality and fertility: Longitudinal evidence from Malawi. *Demographic Research* 2020; **42**: 1057–96.

21 Smith C, Gold J, D Ngo T, Sumpter C, Free C. Mobile phone-based interventions for improving contraception use. London: Cochrane Database of Systematic Reviews, 2015.

22 Coleman J, Bohlin KC, Thorson A, *et al.* Effectiveness of an SMS-based maternal mHealth intervention to improve clinical outcomes of HIV-positive pregnant women. *AIDS Care* 2017; **29**: 890–7.

23 Hall CS, Fottrell E, Wilkinson S, Byass P. Assessing the impact of mHealth interventions in low- and middle-income countries - what has been shown to work? *Global Health Action* 2014; **7**. DOI:10.3402/gha.v7.25606.

24 Lund S, Nielsen BB, Hemed M, *et al.* Mobile phones improve antenatal care attendance in Zanzibar: A cluster randomized controlled trial. *BMC Pregnancy and Childbirth* 2014; **14**: 1–0.

25 Noordam AC, Kuepper BM, Stekelenburg J, Milen A. Improvement of maternal health services through the use of mobile phones. *Tropical Medicine and International Health* 2011; **16**: 622–6.

26 LeFevre AE, Shah N, Harrisson Bashingwa JJ, George AS, Mohan D. Does women's mobile phone ownership matter for health? Evidence from15 countries. *BMJ Global Health* 2020; **5**: e002524.

For peer review only

27 Finlay JE, Mejía-Guevara I, Akachi Y. Inequality in total fertility rates and the proximate determinants of fertility in 21 sub-Saharan African countries. *PLoS ONE* 2018; **13**: e0203344.

28 Rogers RG, Wofford S. Life expectancy in less developed countries: Socioeconomic development or public health? *Journal of Biosocial Science* 2008; **21**: 245–52.

29 The DHS Program. Demographic and Health Surveys (DHS). 2022. http://www.dhsprogram.com/Data/.

30 R Core Team. R: A language and environment for statistical computing. R version 4.2.1 patched. Vienna, Austria: R Foundation for Statistical Computing, 2022 https://www.R-project.org/.

31 Watson OJ, FitzJohn R, Eaton JW. Rdhs: An R package to interact with the Demographic and Health Surveys (DHS) Program datasets. *Wellcome Open Research* 2019; **4**: 103–25.

32 Wickham H, Averick M, Bryan J, *et al.* Welcome to the Tidyverse. *Journal of Open Source Software* 2019; **4**: 1686–91.

33 Eaton J, Masquelier B. Demogsurv: Demographic analysis of DHS and other household surveys. R package version 0.2.5. 2021 https://github.com/mrc-ide/demogsurv.

34 Lumley T. Analysis of complex survey samples. *Journal of Statistical Software* 2004; **9**: 1–9.

35 Robinson D, Hayes A, Couch S. broom: Convert statistical analysis objects into tidy tibbles. R package version 0.7.12. 2022 https://CRAN.R-project.org/package=broom.

36 Pasek J. Anesrake: ANES Raking Implementation. R package version 0.80. 2018 https://CRAN.R-project.org/package=anesrake.

37 Mills BR. MetBrewer: Color palettes inspired by works at the Metropolitan Museum of Art. R package version 0.2.0. 2022 https://CRAN.R-project.org/package=MetBrewer.

38 Zhu H. kableExtra: Construct complex table with 'kable' and pipe syntax. R package version 1.3.4. 2021 https://CRAN.R-project.org/package=kableExtra.

39 Xie Y. Knitr: A general-purpose package for dynamic report generation in R. R package version 1.37. 2021 https://yihui.org/knitr/.

For peer review only

Figure 1: Unadjusted and adjusted estimates of the effect of owning and having access to a mobile phone on under-five mortality from Poisson regressions.

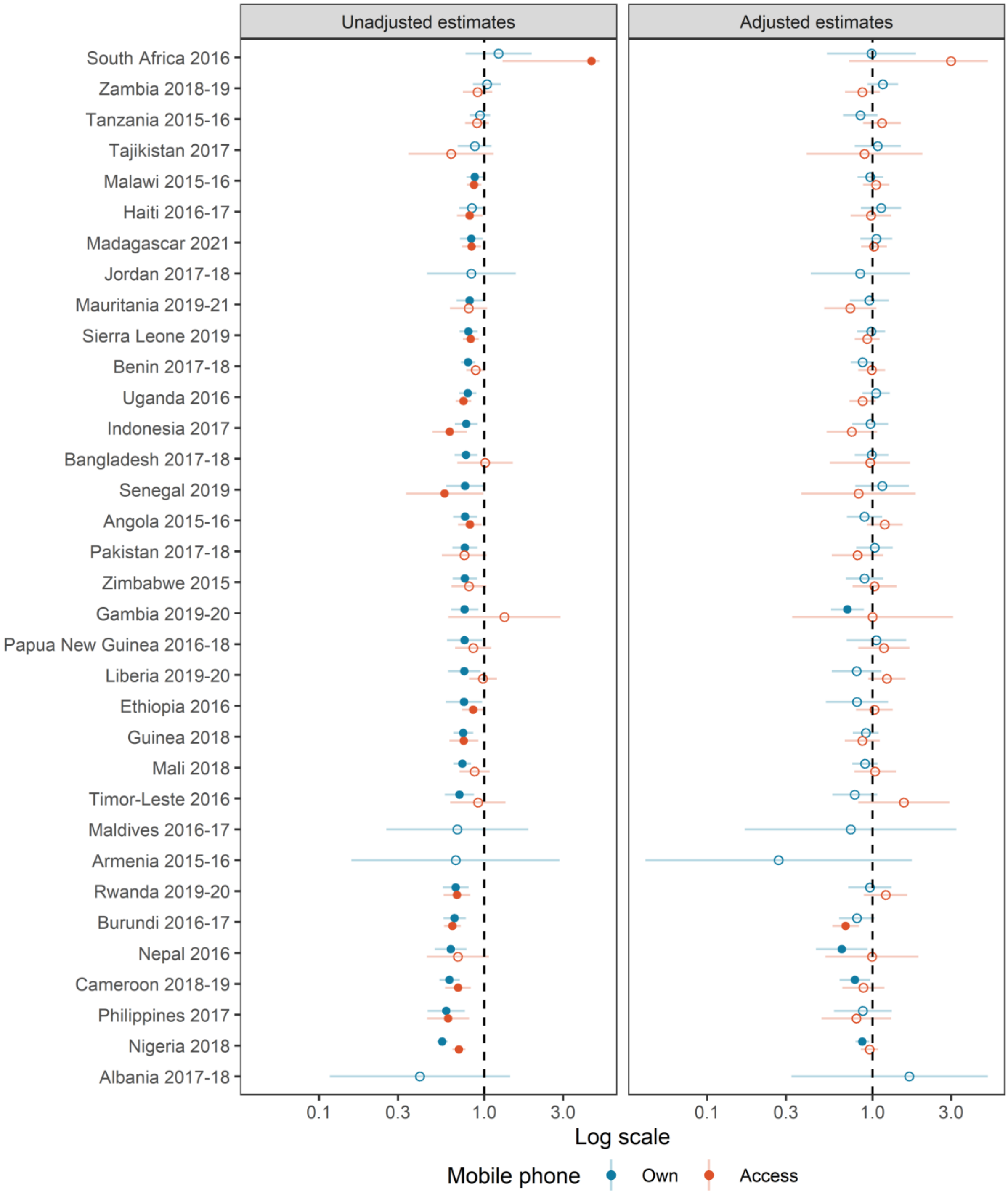

Note: Estimates for access to a mobile phone are not presented for Albania, Armenia, Jordan and Maldives, as access is almost 100%. Fill dots mean that estimates are statistically significant at the 95% confidence level, while empty dots mean that estimates are not statistically significant.

Figure 2: Unadjusted and adjusted estimates of the effect of owning and having access to a mobile phone on fertility from Poisson regressions.

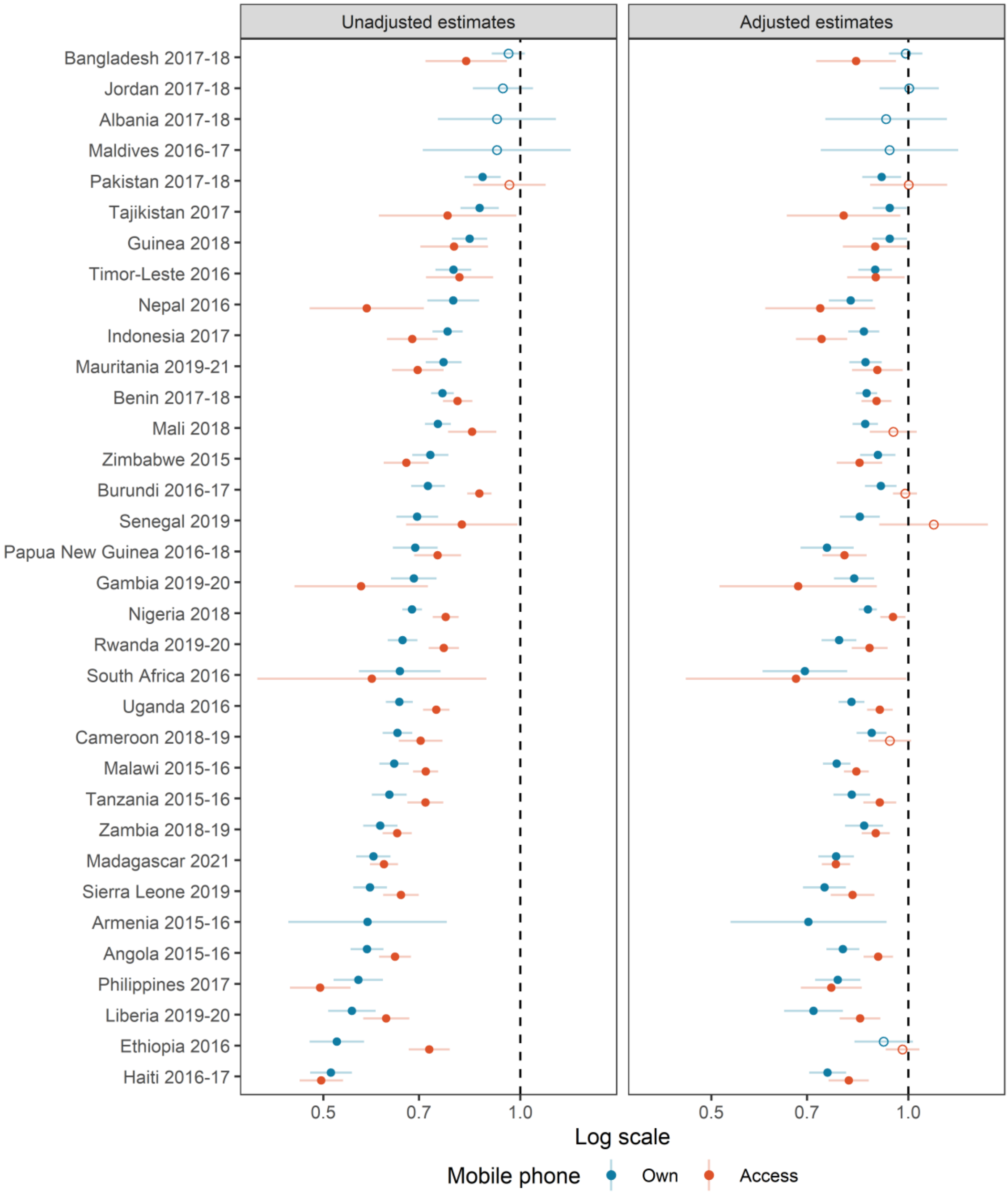

Note: Estimates for access to a mobile phone are not presented for Albania, Armenia, Jordan and Maldives, as access is almost 100%. Fill dots mean that estimates are statistically significant at the 95% confidence level, while empty dots mean that estimates are not statistically significant.

Figure 3: Under-five mortality rate and total fertility rate estimates testing for post-stratification weights from women who own a mobile phone.

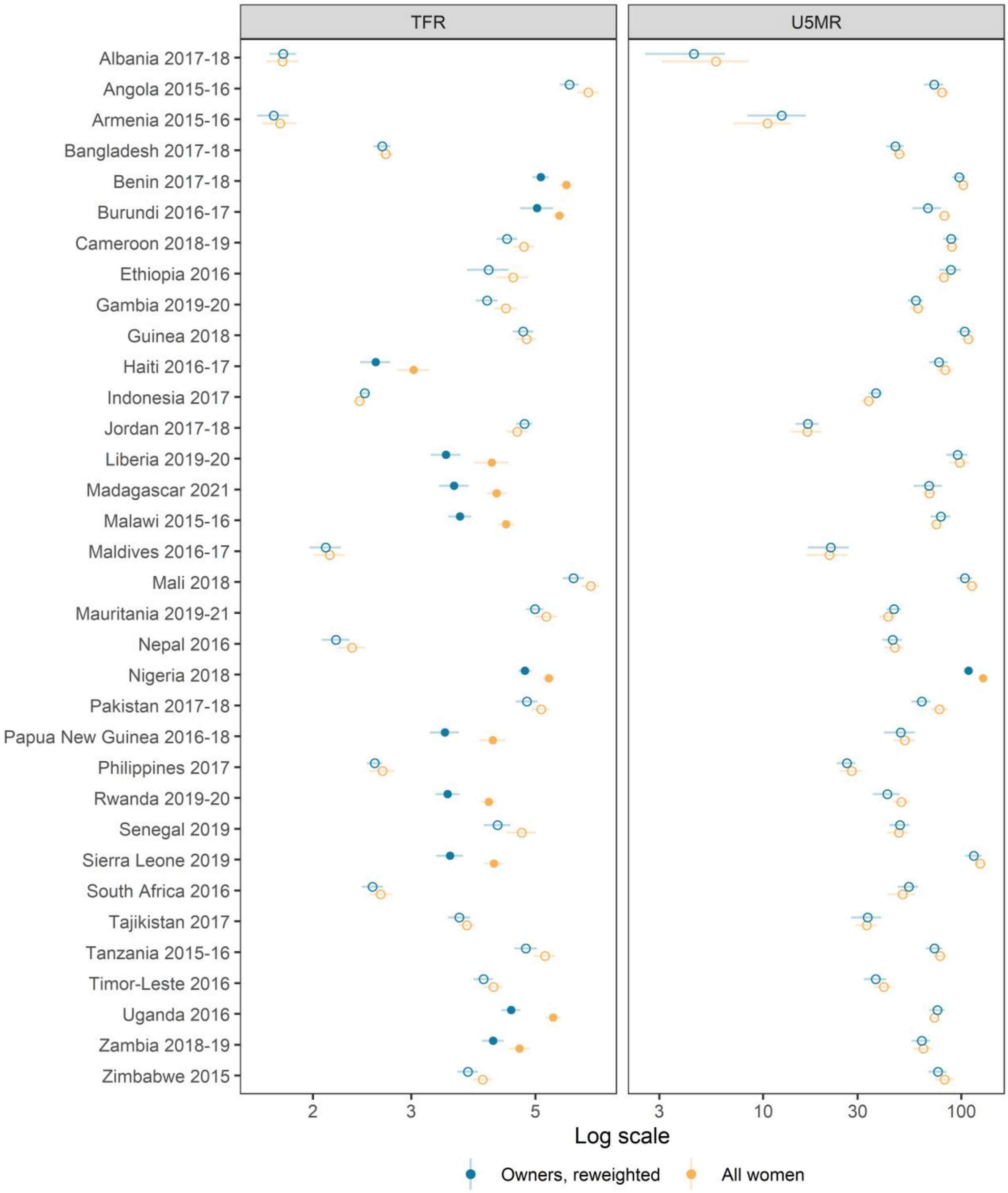

Note: Fill dots mean that the difference between the actual rate and the re-weighted rate is statistically significant at the 95% confidence level, while empty dots mean that the difference is not statistically significant.

Figure 4: Estimates of the difference in current use of modern contraceptives (MCU), sexual activity (SexAct), duration of postpartum amenorrhea (Amen), and duration of postpartum abstinence (Abst), between owners and non-owners of a mobile phone.

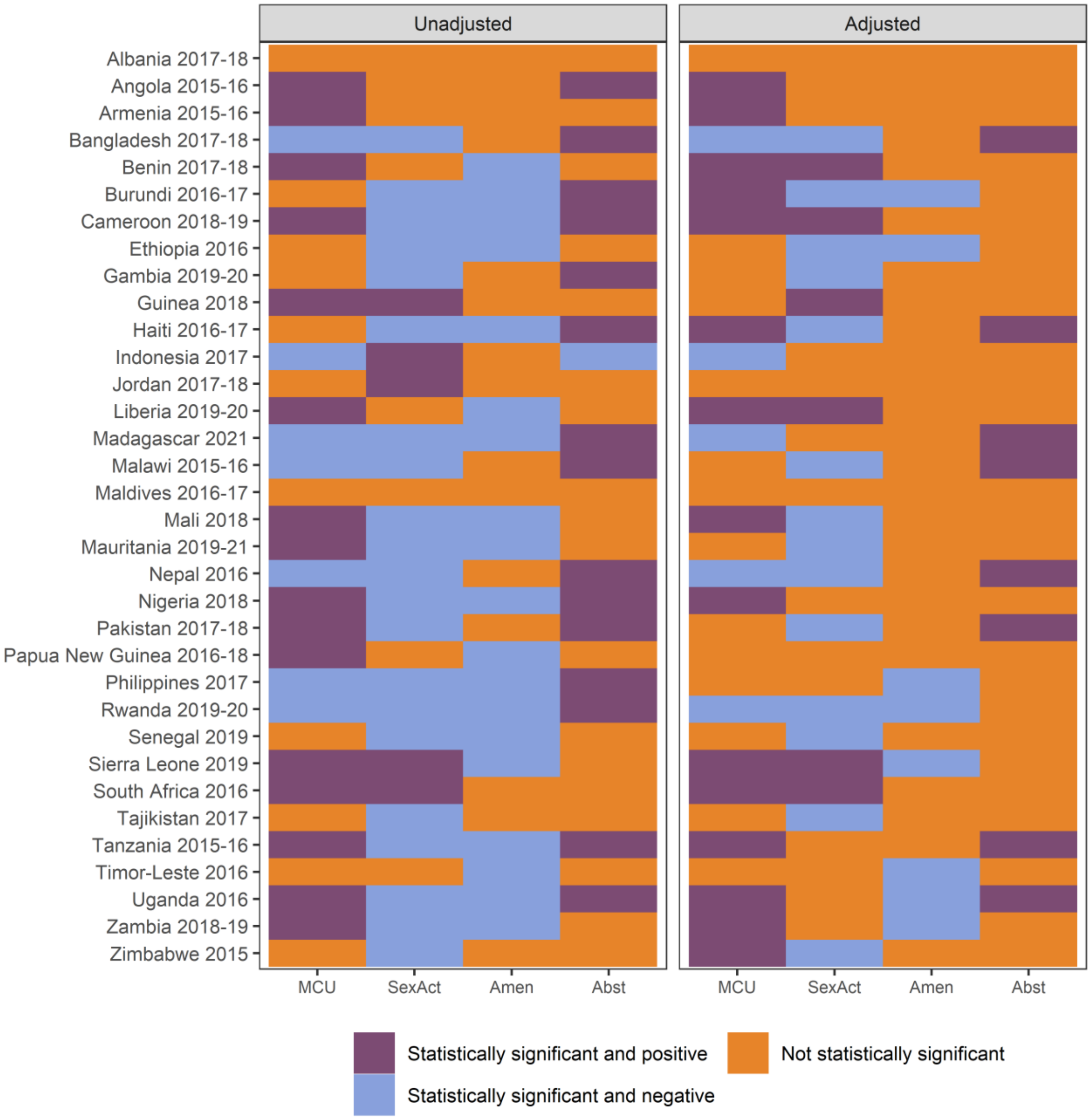

Figure 5: Estimates of the difference in exclusive breastfeeding (BrFeed), births who had at least 4 antenatal care visits (ANC), newborns who received at least 2 signal functions (PNC), children who received basic immunization (Vax), births delivered by skilled professionals (Skill), children who are underweight (UnderW), between owners and non-owners of a mobile phone.

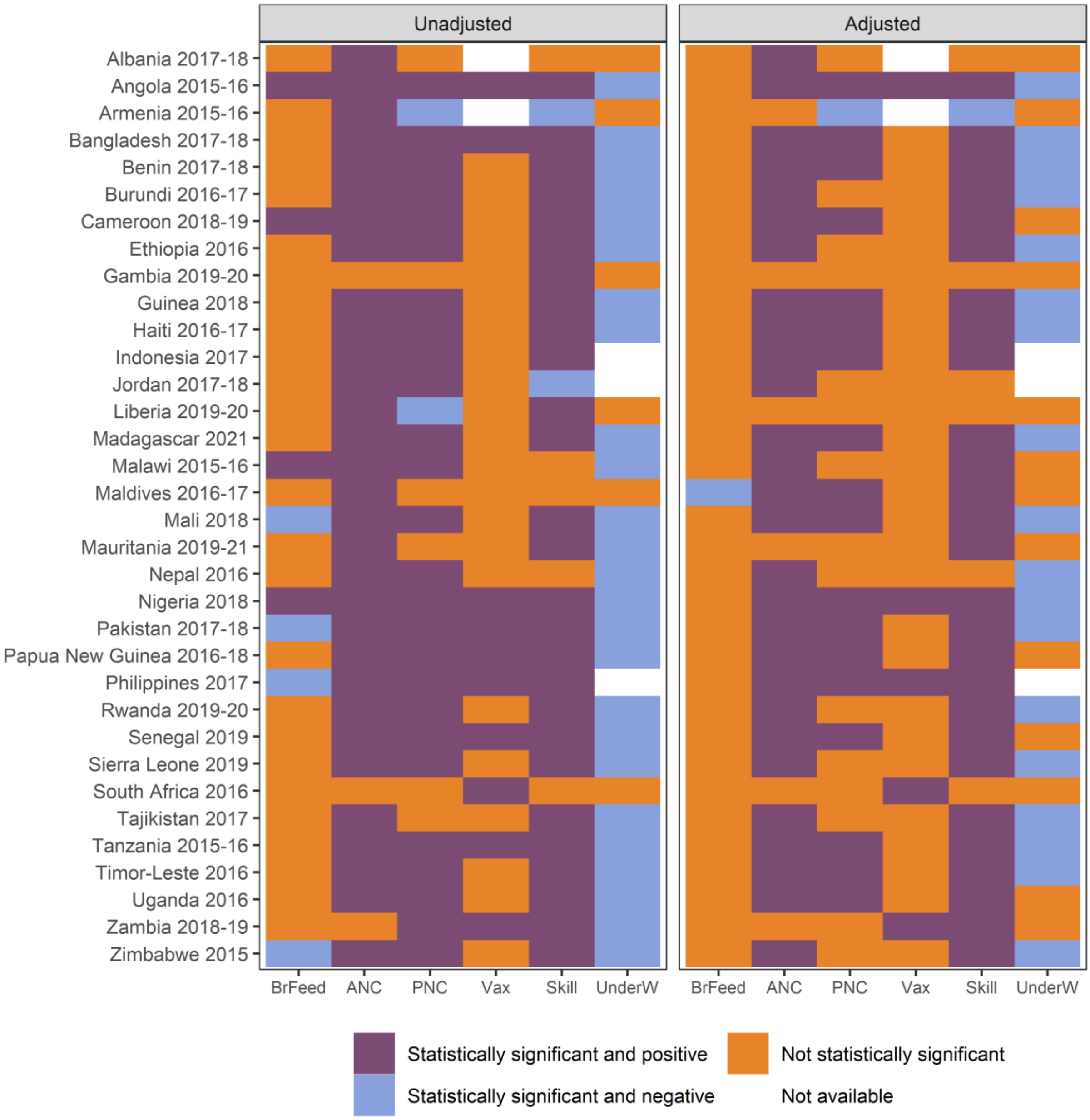

Appendix Table 1: DHS including information on mobile phone ownership and access, and total fertility rate (TFR) for the 3-year period before the survey and under-five mortality rate (U5MR) for the 10-year period before the survey by mobile phone status.

		Mobile phone ownership					Access to a mobile phone				
		Owns a mobile phone			Does not own a mobile phone		Can access a mobile phone			Cannot access a mobile phone	
Survey	Sample size	% of women	TFR	U5MR	TFR	U5MR	% of women	TFR	U5MR	TFR	U5MR
Albania 2017-18	10,970	90.1	1.8	5.1	2.0	12.7	99.3	1.8	5.7	3.0	18.8
Angola 2015-16	14,379	51.2	4.7	67.9	8.0	89.1	75.3	5.5	74.0	8.4	92.3
Armenia 2015-16	6,116	96.7	1.7	10.3	3.0	15.5	99.8	1.7	10.5	*NA*	*NA*
Bangladesh 2017-18	20,127	60.2	2.2	43.9	2.3	56.6	97.2	2.2	48.7	2.7	46.7
Benin 2017-18	15,928	51.1	5.0	91.5	6.7	113.2	90.4	5.5	100.0	7.1	117.0
Burundi 2016-17	17,269	23.6	4.4	58.8	5.9	88.3	55.0	5.3	65.3	5.9	99.0
Cameroon 2018-19	13,901	63.2	4.0	72.1	6.4	115.4	91.5	4.6	85.0	6.0	126.6
Ethiopia 2016	15,683	27.3	2.8	65.8	5.2	84.1	62.1	4.0	74.9	5.5	88.7
Gambia 2019-20	11,865	76.4	4.1	56.0	6.1	75.2	99.6	4.4	60.3	8.1	69.3
Guinea 2018	10,874	68.9	4.6	98.7	5.5	129.6	94.1	4.8	105.9	6.0	141.9
Haiti 2016-17	14,371	57.1	2.3	75.9	4.4	89.8	86.3	2.7	78.1	5.3	98.9
Indonesia 2017	49,569	78.5	2.3	31.9	3.2	40.5	96.3	2.4	33.1	3.7	49.8
Jordan 2017-18	14,689	91.8	2.7	16.5	2.9	19.2	99.2	2.7	16.8	3.0	7.9
Liberia 2019-20	8,065	46.7	2.9	84.0	5.4	107.9	78.6	3.7	96.8	5.9	101.6
Madagascar 2021	18,869	34.6	3.0	60.1	5.0	72.5	61.3	3.2	63.7	5.0	71.7

For peer review only

Malawi 2015-16	24,562	32.8	3.2	67.7	5.1	77.9	62.4	3.8	69.9	5.4	81.4
Maldives 2016-17	7,699	95.5	2.1	21.5	2.4	27.7	100.0	2.1	21.6	*NA*	*NA*
Mali 2018	10,519	58.3	5.5	97.1	7.4	130.1	94.3	6.2	111.8	7.4	126.3
Mauritania 2019-21	15,714	76.7	4.9	40.6	6.8	49.5	96.1	5.1	41.7	7.5	57.7
Nepal 2016	12,862	72.6	2.2	40.3	3.1	62.8	97.3	2.3	45.3	4.0	64.9
Nigeria 2018	41,821	55.3	4.4	93.0	6.5	163.6	92.6	5.2	123.3	6.6	181.5
Pakistan 2017-18	12,363	39.2	3.2	64.4	3.8	85.1	95.4	3.5	77.0	4.1	88.8
Papua New Guinea 2016-18	15,141	34.3	3.2	40.6	4.7	56.2	66.6	3.7	48.5	5.1	56.4
Philippines 2017	25,074	85.8	2.5	24.6	4.3	41.4	95.5	2.5	27.0	5.2	36.8
Rwanda 2019-20	14,634	47.9	3.4	38.6	5.1	59.3	79.1	3.9	44.4	5.1	64.4
Senegal 2019	8,649	69.7	4.2	44.3	6.3	57.1	98.9	4.7	47.5	5.9	98.7
Sierra Leone 2019	15,574	42.6	3.1	110.3	5.2	132.6	81.4	3.9	118.2	5.9	142.0
South Africa 2016	8,514	91.2	2.6	51.2	4.1	43.7	98.8	2.6	51.2	4.1	10.5
Tajikistan 2017	10,718	53.7	3.5	31.5	4.1	35.6	98.1	3.7	32.6	5.1	64.4
Tanzania 2015-16	13,266	52.2	4.1	75.4	6.7	80.8	86.2	4.9	75.6	6.8	88.8
Timor-Leste 2016	12,607	65.6	3.9	35.5	5.0	49.4	94.3	4.2	40.8	5.1	38.1
Uganda 2016	18,506	45.5	4.3	65.2	6.7	79.7	81.8	5.1	68.6	6.8	89.7
Zambia 2018-19	13,683	53.0	3.7	65.4	6.1	63.4	81.2	4.2	62.7	6.6	69.1
Zimbabwe 2015	9,955	69.5	3.7	75.6	5.1	99.3	92.2	3.9	79.8	5.8	102.2

Note: Fertility and mortality rates for women who cannot access a mobile phone cannot be computed for Armenia and Maldives due to the small sample size.

For peer review only

Appendix Figure 1.1: Effect of the 15-19 age group on the odds of owning and having access to a mobile phone from binomial logistic regressions (20-24 age group is the reference). X-axis in log scale.

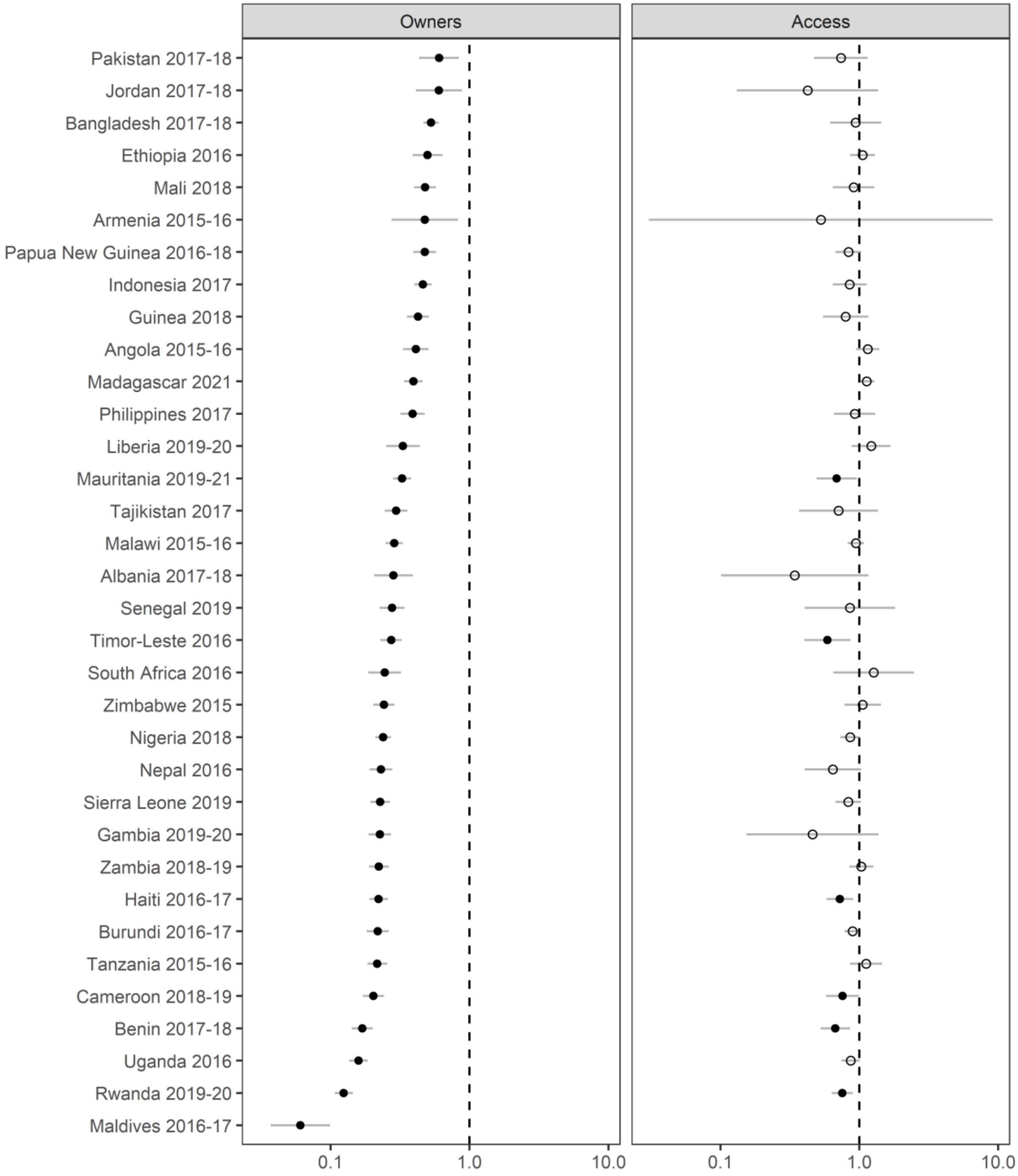

Note: Estimates for access to a mobile phone cannot be computed for Maldives due to small sample size. Fill dots mean that estimates are statistically significant at the 95% confidence level, while empty dots mean that estimates are not statistically significant.

Appendix Figure 1.2: Effect of the 25-29 age group on the odds of owning and having access to a mobile phone from binomial logistic regressions (20-24 age group is the reference). X-axis in log scale.

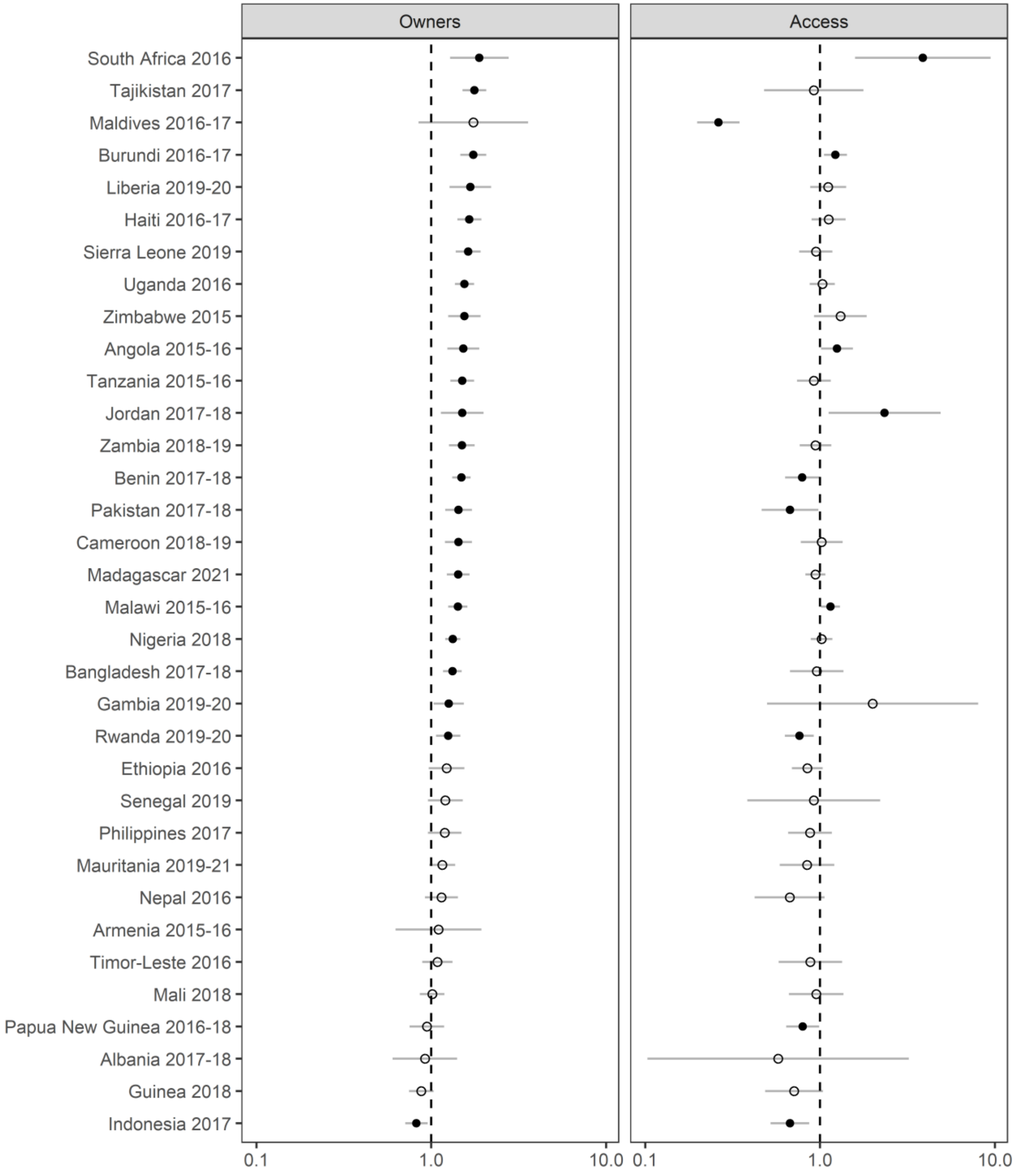

Note: Estimates for access to a mobile phone cannot be computed for Armenia due to small sample size. Fill dots mean that estimates are statistically significant at the 95% confidence level, while empty dots mean that estimates are not statistically significant.

Appendix Figure 1.3: Effect of the 30-34 age group on the odds of owning and having access to a mobile phone from binomial logistic regressions (20-24 age group is the reference). X-axis in log scale.

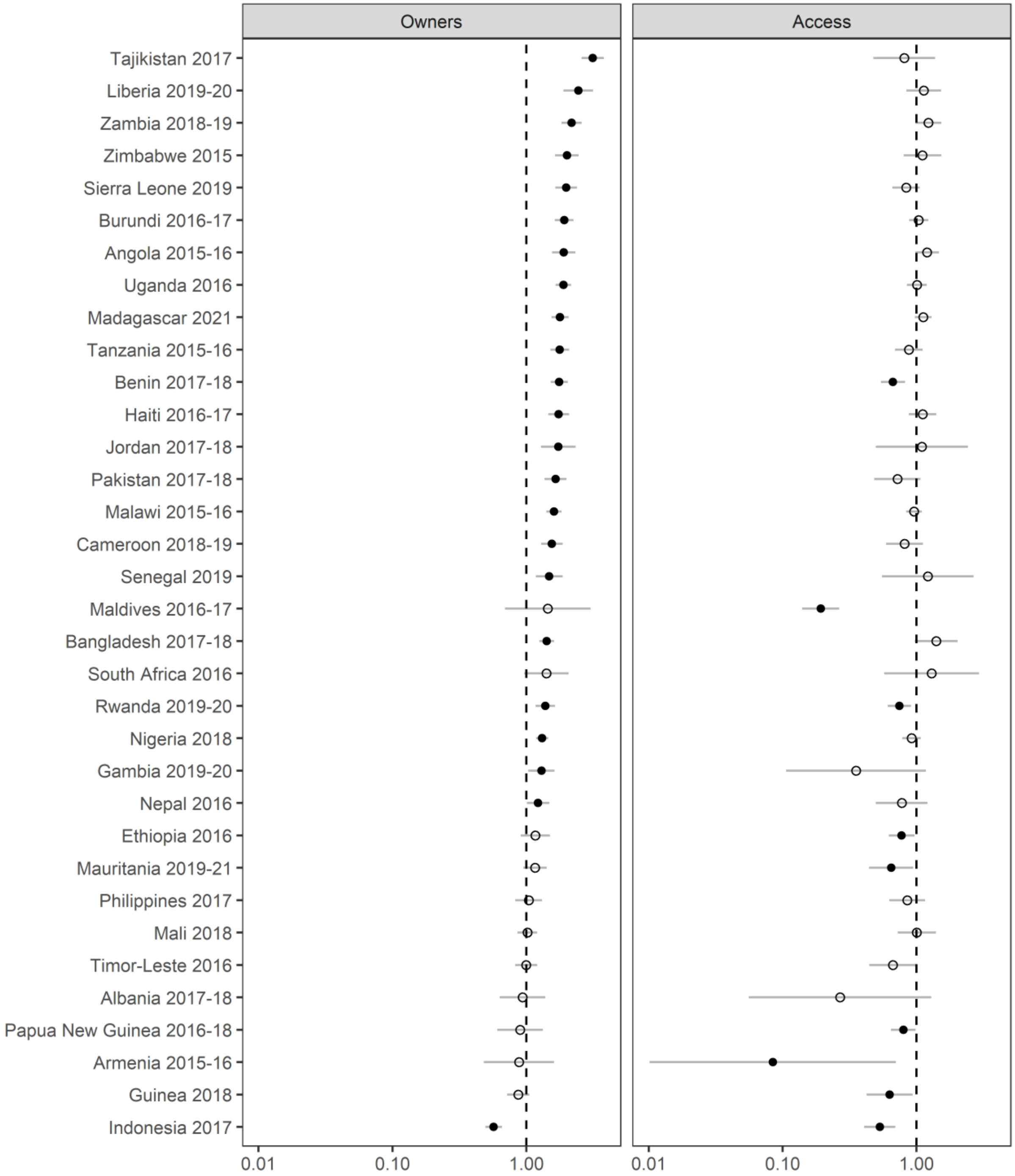

Note: Fill dots mean that estimates are statistically significant at the 95% confidence level, while empty dots mean that estimates are not statistically significant.

Appendix Figure 1.4: Effect of the 35-39 age group on the odds of owning and having access to a mobile phone from binomial logistic regressions (20-24 age group is the reference). X-axis in log scale.

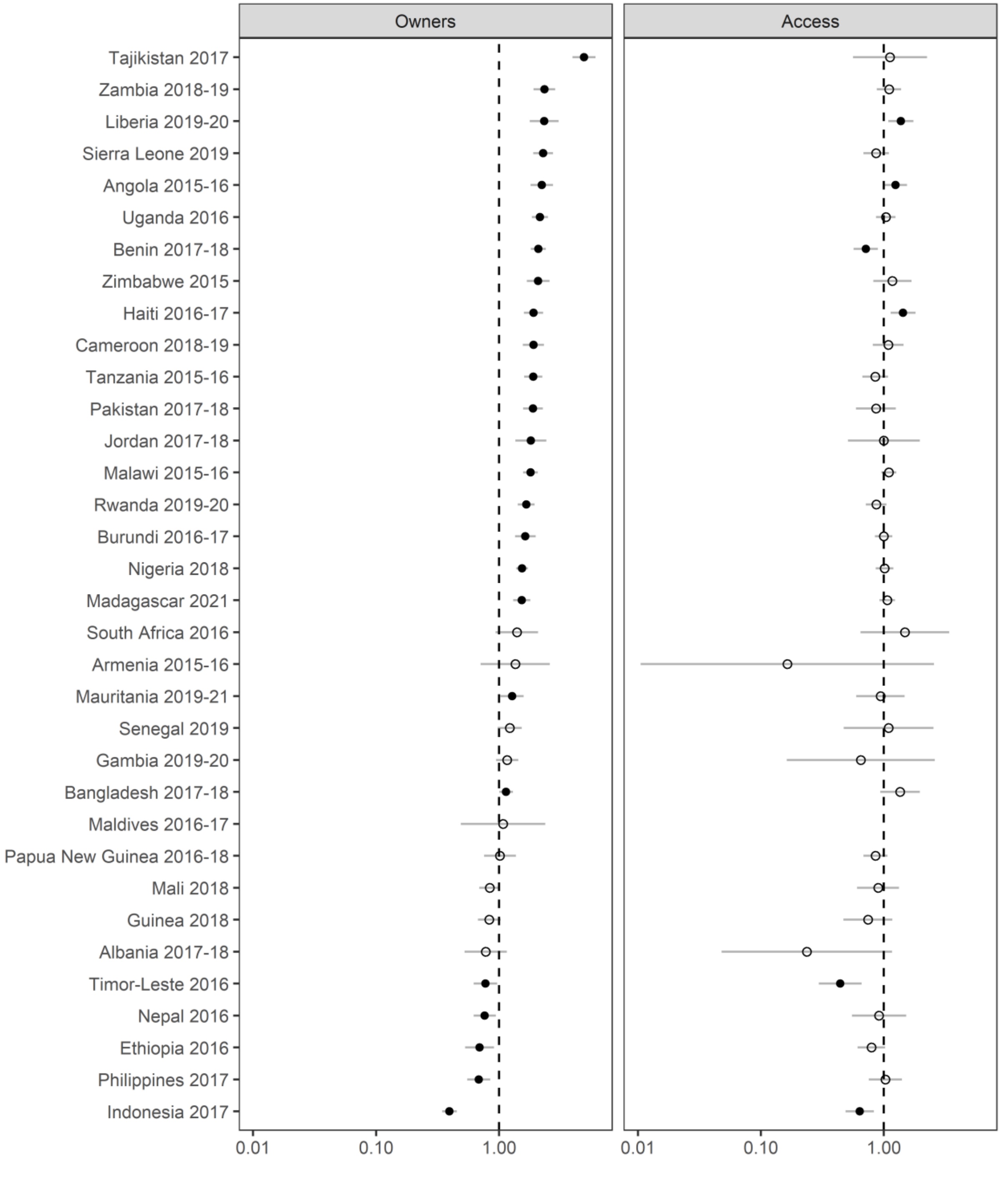

Note: Estimates for access to a mobile phone cannot be computed for Maldives due to small sample size. Fill dots mean that estimates are statistically significant at the 95% confidence level, while empty dots mean that estimates are not statistically significant.

Appendix Figure 1.5: Effect of the 40-44 age group on the odds of owning and having access to a mobile phone from binomial logistic regressions (20-24 age group is the reference). X-axis in log scale.

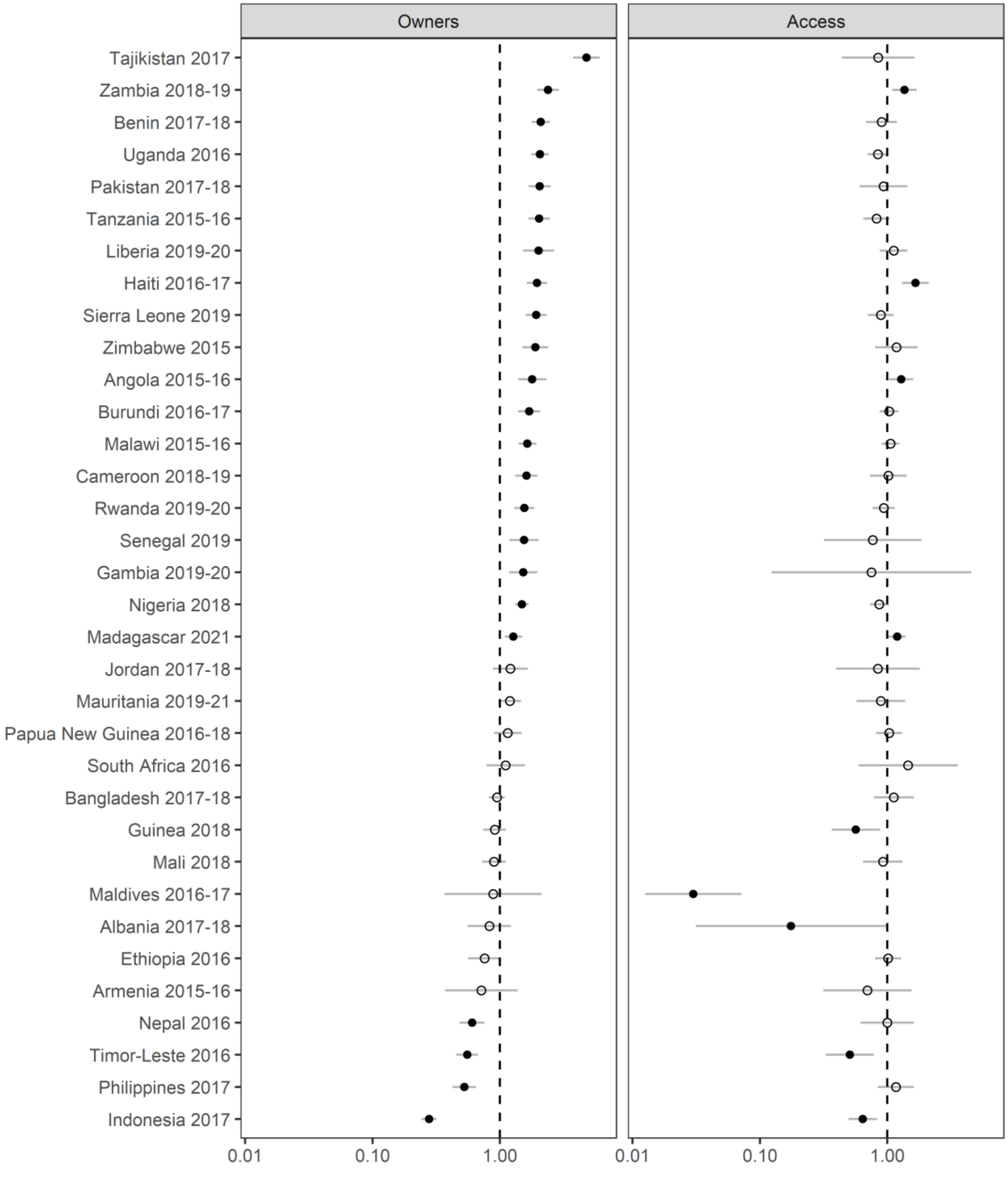

Note: Fill dots mean that estimates are statistically significant at the 95% confidence level, while empty dots mean that estimates are not statistically significant.

Appendix Figure 1.6: Effect of the 45-49 age group on the odds of owning and having access to a mobile phone from binomial logistic regressions (20-24 age group is the reference). X-axis in log scale.

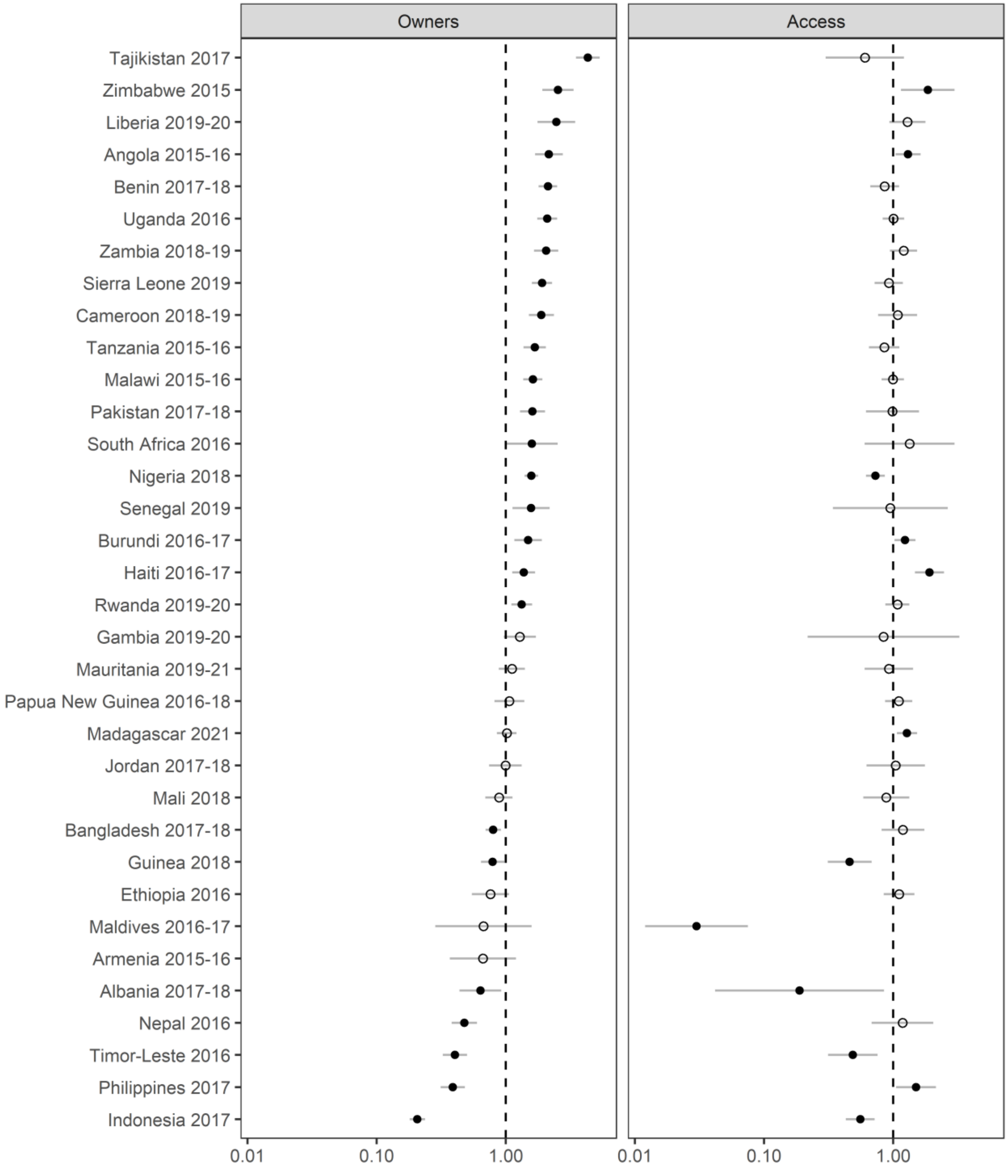

Note: Estimates for access to a mobile phone cannot be computed for Armenia due to small sample size. Fill dots mean that estimates are statistically significant at the 95% confidence level, while empty dots mean that estimates are not statistically significant.

Appendix Figure 2.1: Effect of primary education on the odds of owning and having access to a mobile phone from binomial logistic regressions (no education is the reference). X-axis in log scale.

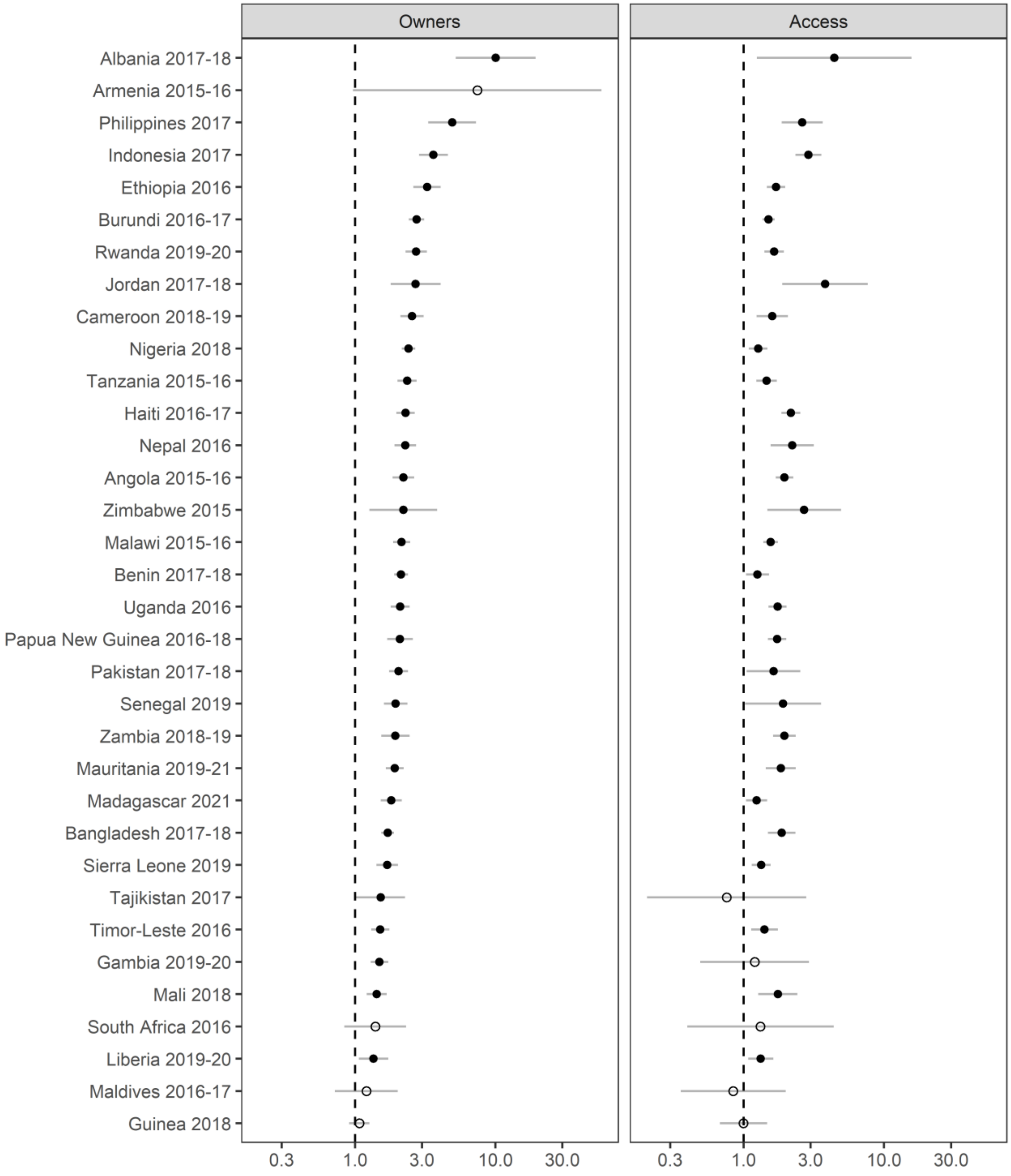

Note: Estimates for access to a mobile phone cannot be computed for Armenia due to small sample size. Fill dots mean that estimates are statistically significant at the 95% confidence level, while empty dots mean that estimates are not statistically significant.

Appendix Figure 2.2: Effect of secondary and higher education on the of odds owning and having access to a mobile phone from binomial logistic regressions (no education is the reference). X-axis in log scale.

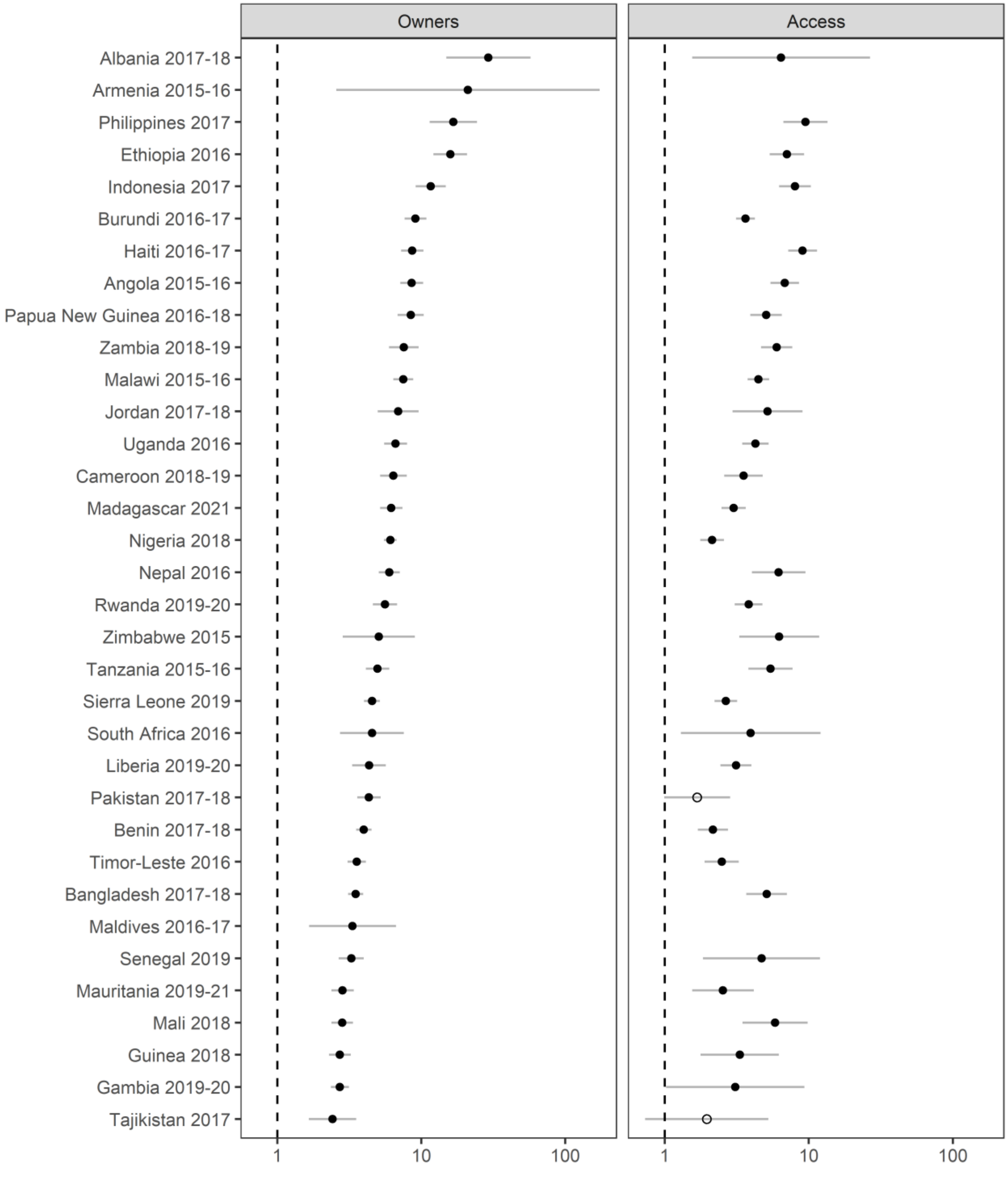

Note: Estimates for access to a mobile phone cannot be computed for Armenia and Maldives due to small sample size. Fill dots mean that estimates are statistically significant at the 95% confidence level, while empty dots mean that estimates are not statistically significant.

Appendix Figure 3: Effect of living in urban areas on the odds of owning and having access to a mobile phone from binomial logistic regressions (rural area is the reference). X-axis in log scale.

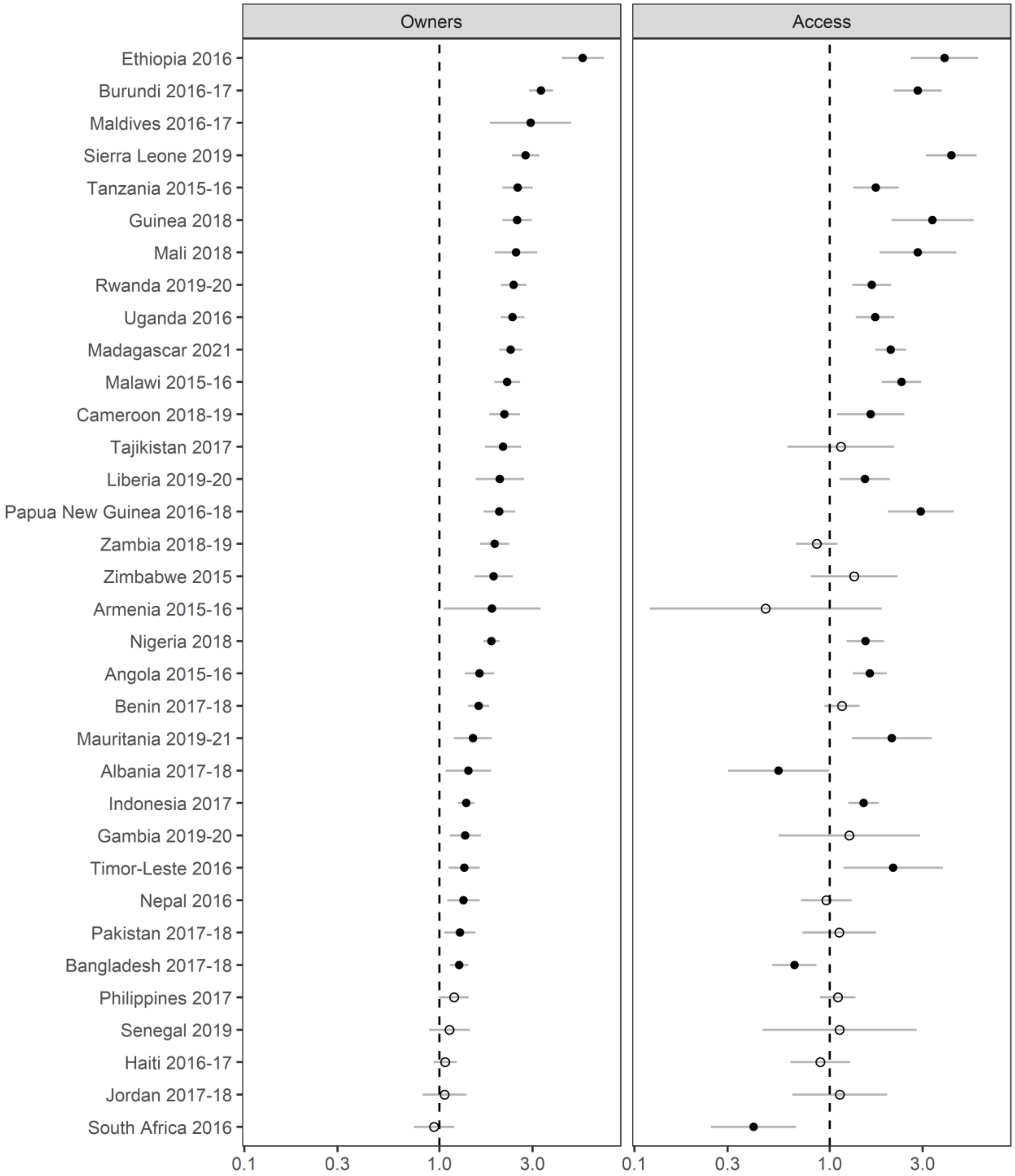

Note: Estimates for access to a mobile phone cannot be computed for Maldives due to small sample size. Fill dots mean that estimates are statistically significant at the 95% confidence level, while empty dots mean that estimates are not statistically significant.

Appendix Figure 4: Effect of being non-poor on the odds of owning and having access to a mobile phone from binomial logistic regressions (poor is the reference). X-axis in log scale.

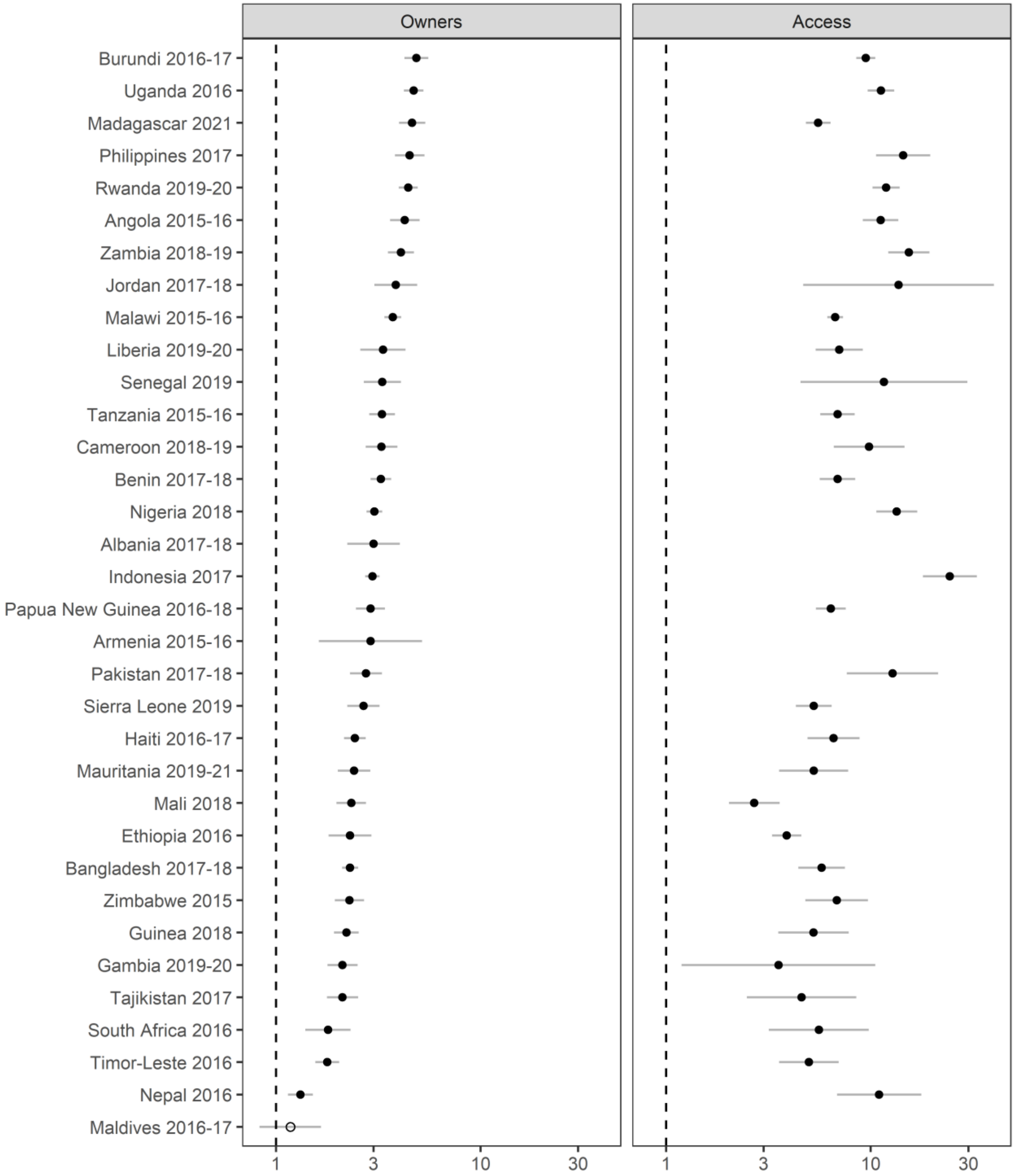

Note: Estimates for access to a mobile phone cannot be computed for Albania, Armenia, and Maldives due to small sample size. Fill dots mean that estimates are statistically significant at the 95% confidence level, while empty dots mean that estimates are not statistically significant.

Appendix Figure 5: Effect of not being in union on the odds of owning and having access to a mobile phone from binomial logistic regressions (married is the reference). X-axis in log scale.

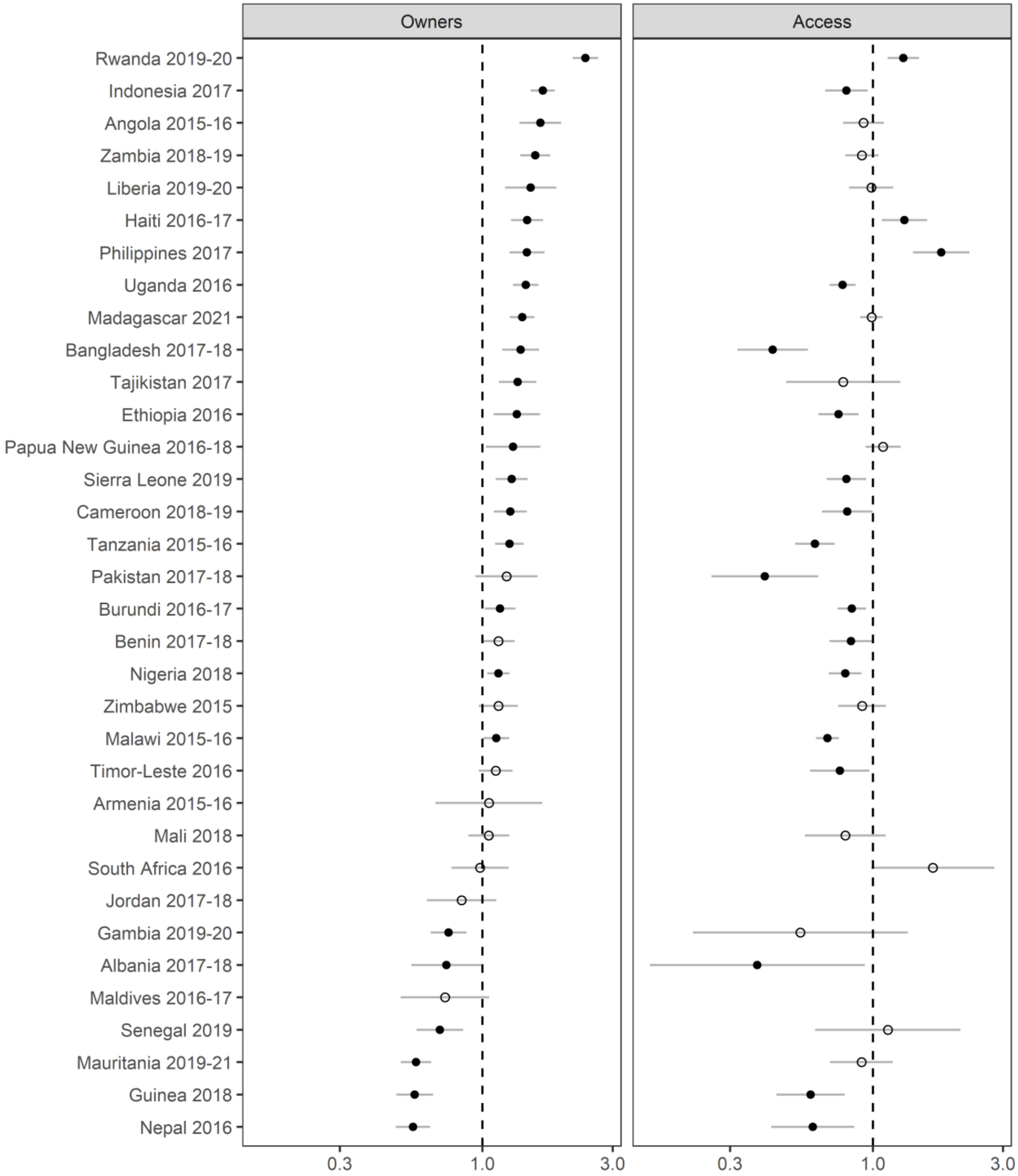

Note: Estimates for access to a mobile phone cannot be computed for Armenia, Jordan, and Maldives due to small sample size. Fill dots mean that estimates are statistically significant at the 95% confidence level, while empty dots mean that estimates are not statistically significant.

Appendix Figure 6: Under-five mortality rate and total fertility rate estimates testing for post-stratification weights from women who have access to a mobile phone.

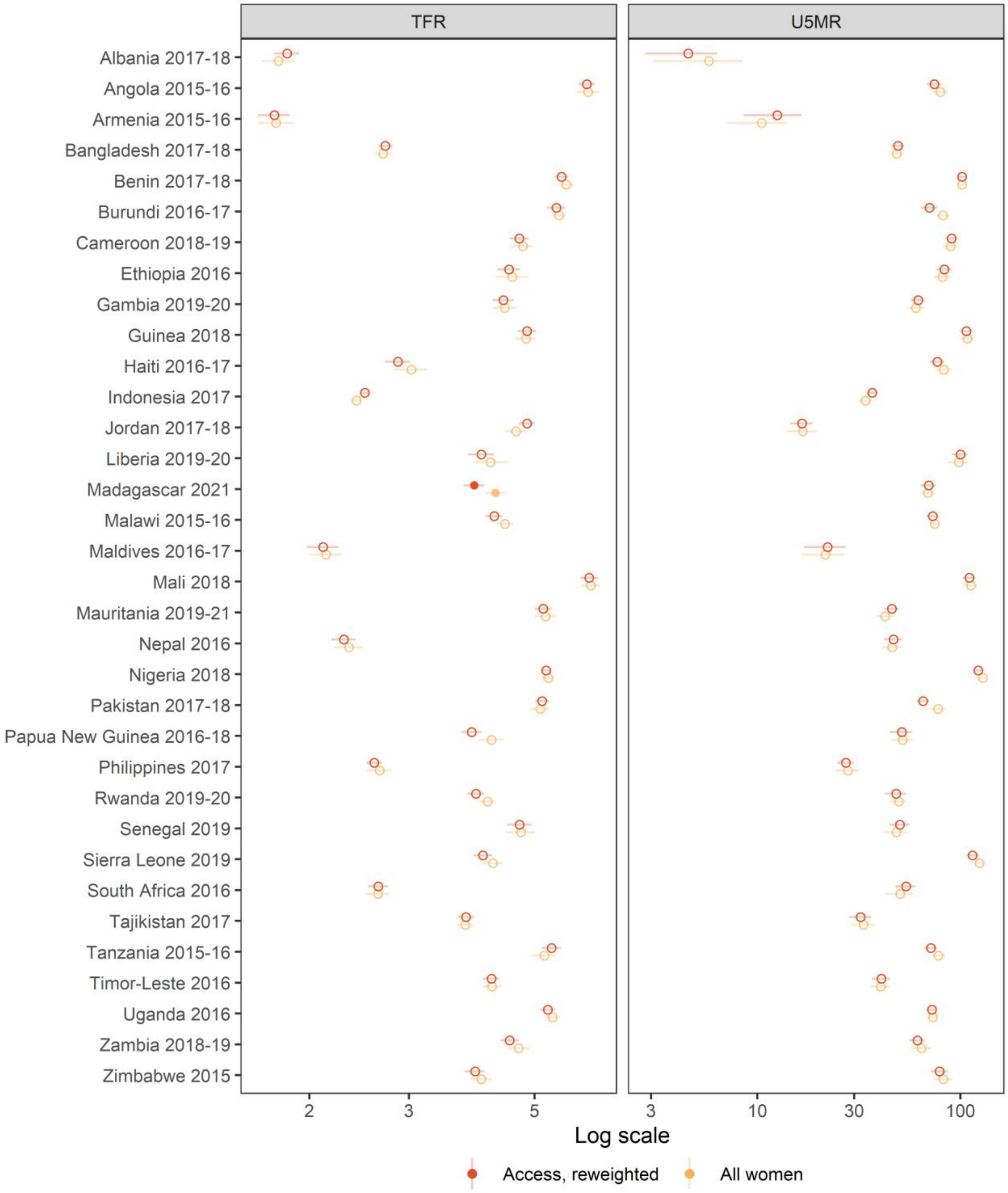

Note: Fill dots mean that the difference between the actual rate and the re-weighted rate is statistically significant at the 95% confidence level, while empty dots mean that the difference is not statistically significant.

Appendix Figure 7: Estimates of the difference in current use of modern contraceptives (MCU), sexual activity (SexAct), duration of postpartum amenorrhea (Amen), and duration of postpartum abstinence (Abst), between having and not having access to a mobile phone.

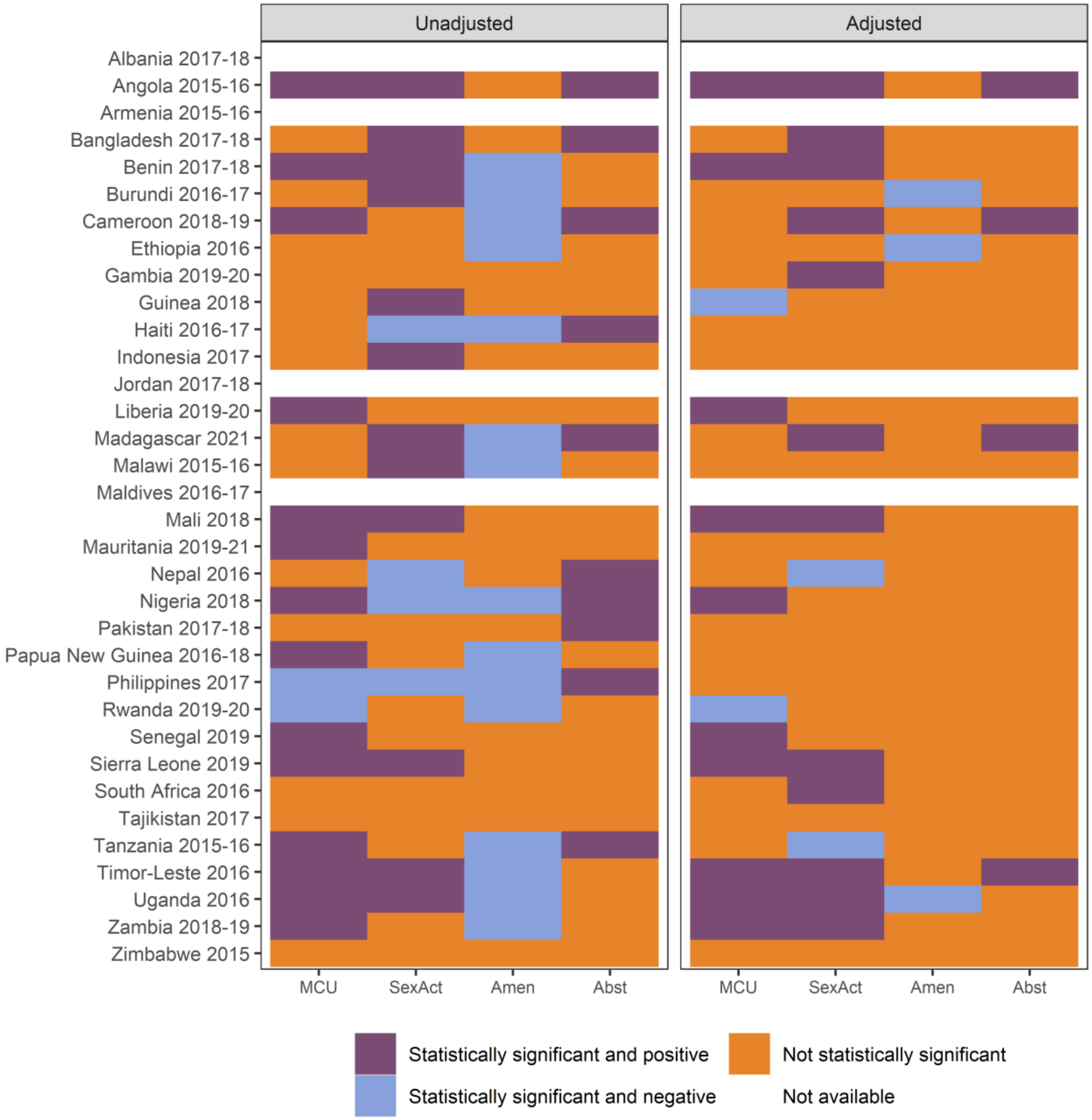

Appendix Figure 8: Estimates of the difference in exclusive breastfeeding (BrFeed), births who had at least 4 antenatal care visits (ANC), newborns who received at least 2 signal functions (PNC), children who received basic immunization (Vax), births delivered by skilled professionals (Skill), children who are underweight (UnderW), between having and not having access to a mobile phone.

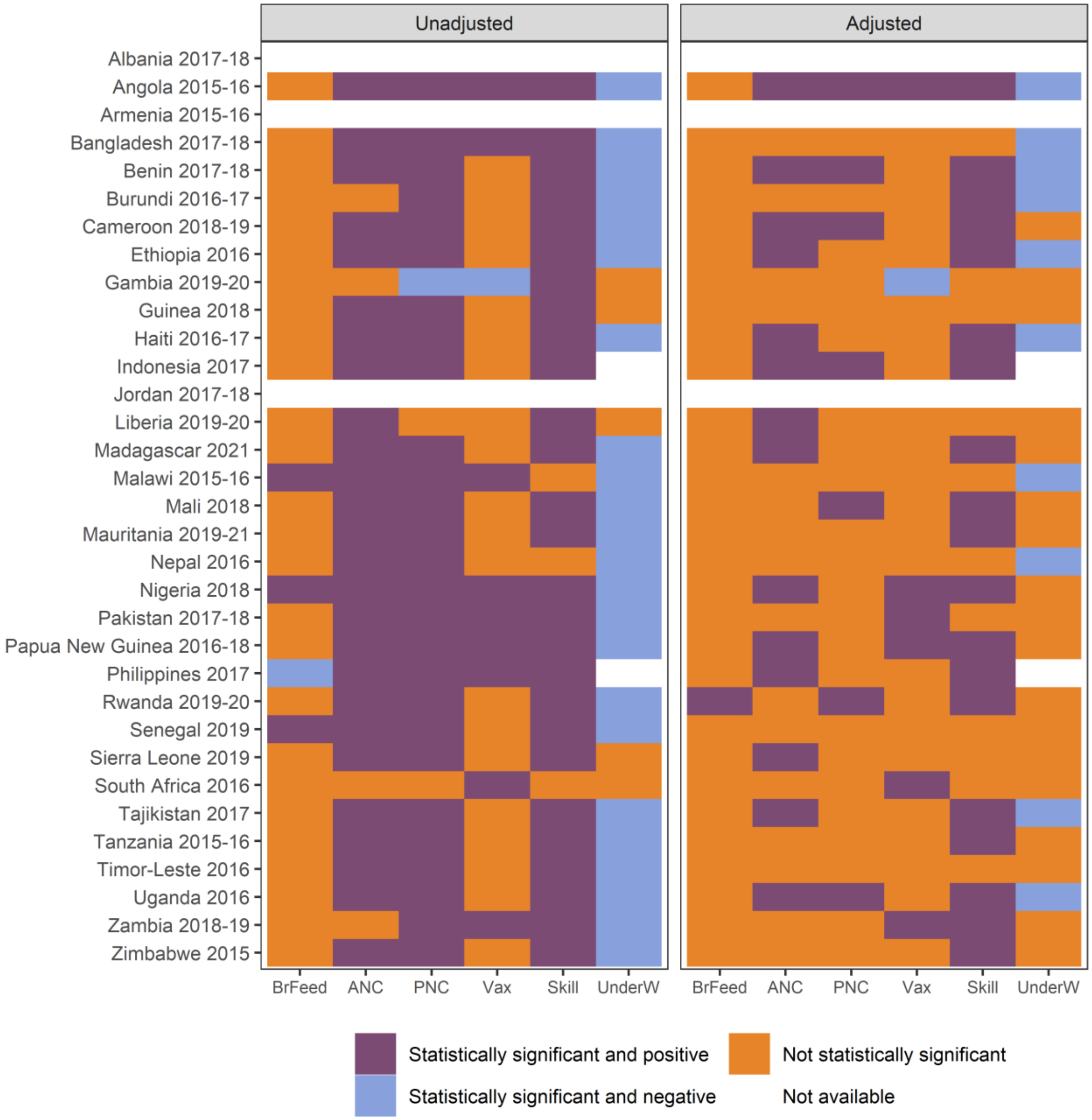

STROBE Statement—Checklist of items that should be included in reports of ***cross-sectional studies***

	Item No	Recommendation	Page No
**Title and abstract**	1	(*a*) Indicate the study's design with a commonly used term in the title or the abstract	2
		(*b*) Provide in the abstract an informative and balanced summary of what was done and what was found	2
**Introduction**			
Background/rationale	2	Explain the scientific background and rationale for the investigation being reported	4-6
Objectives	3	State specific objectives, including any prespecified hypotheses	6
**Methods**			
Study design	4	Present key elements of study design early in the paper	6-8
Setting	5	Describe the setting, locations, and relevant dates, including periods of recruitment, exposure, follow-up, and data collection	7
Participants	6	(*a*) Give the eligibility criteria, and the sources and methods of selection of participants	NA
Variables	7	Clearly define all outcomes, exposures, predictors, potential confounders, and effect modifiers. Give diagnostic criteria, if applicable	7-9
Data sources/ measurement	8*	For each variable of interest, give sources of data and details of methods of assessment (measurement). Describe comparability of assessment methods if there is more than one group	6-7
Bias	9	Describe any efforts to address potential sources of bias	7-9
Study size	10	Explain how the study size was arrived at	6-7
Quantitative variables	11	Explain how quantitative variables were handled in the analyses. If applicable, describe which groupings were chosen and why	7-9
Statistical methods	12	(*a*) Describe all statistical methods, including those used to control for confounding	7-9
		(*b*) Describe any methods used to examine subgroups and interactions	8-9
		(*c*) Explain how missing data were addressed	7
		(*d*) If applicable, describe analytical methods taking account of sampling strategy	NA
		(*e*) Describe any sensitivity analyses	NA
**Results**			
Participants	13*	(a) Report numbers of individuals at each stage of study—eg numbers potentially eligible, examined for eligibility, confirmed eligible, included in the study, completing follow-up, and analysed	NA
		(b) Give reasons for non-participation at each stage	NA
		(c) Consider use of a flow diagram	NA
Descriptive data	14*	(a) Give characteristics of study participants (eg demographic, clinical, social) and information on exposures and potential confounders	21-22
		(b) Indicate number of participants with missing data for each variable of interest	7
Outcome data	15*	Report numbers of outcome events or summary measures	9-10
Main results	16	(*a*) Give unadjusted estimates and, if applicable, confounder-adjusted estimates and their precision (eg, 95% confidence interval). Make clear which confounders were adjusted for and why they were included	10-11

For peer review only

		(*b*) Report category boundaries when continuous variables were categorized	NA
		(*c*) If relevant, consider translating estimates of relative risk into absolute risk for a meaningful time period	NA
Other analyses	17	Report other analyses done—eg analyses of subgroups and interactions, and sensitivity analyses	11-13
**Discussion**			
Key results	18	Summarise key results with reference to study objectives	13-16
Limitations	19	Discuss limitations of the study, taking into account sources of potential bias or imprecision. Discuss both direction and magnitude of any potential bias	15-16
Interpretation	20	Give a cautious overall interpretation of results considering objectives, limitations, multiplicity of analyses, results from similar studies, and other relevant evidence	16
Generalisability	21	Discuss the generalisability (external validity) of the study results	16
**Other information**			
Funding	22	Give the source of funding and the role of the funders for the present study and, if applicable, for the original study on which the present article is based	16

*Give information separately for exposed and unexposed groups.

Note: An Explanation and Elaboration article discusses each checklist item and gives methodological background and published examples of transparent reporting. The STROBE checklist is best used in conjunction with this article (freely available on the Web sites of PLoS Medicine at http://www.plosmedicine.org/, Annals of Internal Medicine at http://www.annals.org/, and Epidemiology at http://www.epidem.com/). Information on the STROBE Initiative is available at www.strobe-statement.org.

For peer review only

BMJ Open

Measuring under-five mortality and fertility through mobile phone surveys: an assessment of selection bias in 34 low- and middle-income countries

Journal:	*BMJ Open*
Manuscript ID	bmjopen-2023-071791.R1
Article Type:	Original research
Date Submitted by the Author:	24-May-2023
Complete List of Authors:	Sánchez-Páez, David; Université catholique de Louvain, Centre for Demographic Research<br>Masquelier, Bruno; Université catholique de Louvain, Centre for Demographic Research<br>Menashe-Oren, Ashira; Université catholique de Louvain, Centre de Recherché en Démographie<br>Baruwa, Ololade; University of the Witwatersrand Johannesburg<br>Reniers, Georges; London School of Hygiene & Tropical Medicine, Population Studies Group
**Primary Subject Heading**:	Research methods
Secondary Subject Heading:	Global health, Research methods
Keywords:	Demography < TROPICAL MEDICINE, STATISTICS & RESEARCH METHODS, Epidemiology < TROPICAL MEDICINE

SCHOLARONE™
Manuscripts

I, the Submitting Author has the right to grant and does grant on behalf of all authors of the Work (as defined in the below author licence), an exclusive licence and/or a non-exclusive licence for contributions from authors who are: i) UK Crown employees; ii) where BMJ has agreed a CC-BY licence shall apply, and/or iii) in accordance with the terms applicable for US Federal Government officers or employees acting as part of their official duties; on a worldwide, perpetual, irrevocable, royalty-free basis to BMJ Publishing Group Ltd ("BMJ") its licensees and where the relevant Journal is co-owned by BMJ to the co-owners of the Journal, to publish the Work in this journal and any other BMJ products and to exploit all rights, as set out in our licence.

The Submitting Author accepts and understands that any supply made under these terms is made by BMJ to the Submitting Author unless you are acting as an employee on behalf of your employer or a postgraduate student of an affiliated institution which is paying any applicable article publishing charge ("APC") for Open Access articles. Where the Submitting Author wishes to make the Work available on an Open Access basis (and intends to pay the relevant APC), the terms of reuse of such Open Access shall be governed by a Creative Commons licence – details of these licences and which Creative Commons licence will apply to this Work are set out in our licence referred to above.

Other than as permitted in any relevant BMJ Author's Self Archiving Policies, I confirm this Work has not been accepted for publication elsewhere, is not being considered for publication elsewhere and does not duplicate material already published. I confirm all authors consent to publication of this Work and authorise the granting of this licence.

For peer review only

Measuring under-five mortality and fertility through mobile phone surveys: an assessment of selection bias in 34 low- and middle-income countries

AUTHORS:

1. David A. Sánchez-Páez (Corresponding author).
   Affiliation: Centre for Demographic Research, Université catholique de Louvain.
   Address: Place Montesquieu 1/L2.08.03
   1348 Louvain-la-Neuve
   Belgium.
   E-mail: david.sanchezpaez@uclouvain.be
   ORCID: 0000-0002-7828-8193

2. Bruno Masquelier.
   Affiliation: Centre for Demographic Research, Université catholique de Louvain.
   Address: Louvain-la-Neuve, Belgium.
   ORCID: 0000-0002-9585-891X

3. Ashira Menashe-Oren.
   Affiliation: Centre for Demographic Research, Université catholique de Louvain.
   Address: Louvain-la-Neuve, Belgium.
   ORCID: 0000-0002-4781-5384

4. Ololade Julius Baruwa.
   Affiliation: Centre for Demographic Research, Université catholique de Louvain.
   Address: Louvain-la-Neuve, Belgium.
   ORCID: 0000-0002-0776-528X

5. Georges Reniers.
   Affiliation: Population Studies Group, London School of Hygiene & Tropical Medicine.
   Address: London, United Kingdom.
   ORCID: 0000-0001-6582-1692

Keywords: Under-five mortality; fertility; mobile phone surveys; low- and middle-income countries; selection bias; Demographic and Health Surveys; mobile phone ownership; mobile phone access.

Word count: 3,929 exclusive of title page, abstract, summary box, references, figures, tables, and declaration statements.

ABSTRACT

Introduction: In countries lacking comprehensive systems of civil registration and vital statistics, the collection of demographic data through mobile surveys is increasingly feasible, as mobile phone coverage has rapidly expanded. However, mobile phone users are often younger, male, with higher education, and wealthier, potentially biasing demographic estimates based on phone surveys.

Methods: We assess selection bias in estimates of under-five mortality and fertility using data from 34 Demographic and Health Surveys. We use logistic regressions to characterize individuals who own or have access to a mobile phone in terms of sociodemographic background characteristics, and Poisson regressions to estimate the association between mobile phone ownership (or access) and total fertility and under-five mortality rates. We further evaluate the potential reasons why estimates by mobile phone ownership may differ using a set of behavioral characteristics.

Results: Fertility and mortality rates would be biased downwards if estimates are only based on women who own or have access to mobile phones. Under-five mortality estimates can be adjusted through post-stratification using age, educational level, area of residence, wealth, and marital status as weights. However, fertility estimates remain biased even after adjusting for these covariates. This difference is associated with behavioral factors that are not captured by the post-stratification variables, but for which there are also differences between mobile phone owners and non-owners. In many countries, modern contraceptive use is positively associated with mobile phone ownership, which may complicate fertility estimates based on mobile phone surveys.

Conclusions: Mobile phone surveys need to include background questions on socioeconomic status to be able to weight and adjust mortality estimates ex post-facto. Fertility estimates from mobile phone surveys will be biased unless further research uncovers the mechanisms driving the bias.

For peer review only

Strengths and limitations of this study

- Mobile phone ownership and access is associated with socioeconomic status, which could bias demographic estimates derived from data collected through mobile phone surveys.
- In empirical studies analyzing selection bias in estimates derived from mobile phone surveys we find isolated evidence from individual countries suggesting selection bias in fertility and mortality estimates.
- We established that fertility and under-five mortality estimates derived from birth histories administered for women who own or have access to a mobile phone will be biased downwards.
- Post-stratification on sociodemographic background characteristics corrects the bias in mortality estimates, but adjusted fertility estimates remain lower than in a nationally representative sample, including women who do not own or have access to a mobile phone. This may be due to the fact that fertility decision-making is volitional and not fully captured by women's background and living conditions.
- The prevalence of mobile phone surveys is expected to increase in low- and middle-income countries, providing a valuable means of generating comprehensive statistics on demographic and health factors at the national level. However, mobile phone ownership and access will remain unevenly distributed for some time to come, and as this is likely to be correlated with many of the outcomes of interest, it is important to take this into account in the design of the survey. Post-stratification is only one possible solution to calibrate or increase the external validity of the estimates. Other – complementary – solutions include stratified sampling or more complex designs that combine mobile phone interviews with other data collection modalities.

For peer review only

INTRODUCTION

Use of mobile phones has spread widely across the world, including in low- and middle-income countries (LMIC) where the technological infrastructure for landlines is often lacking[1,2]. This spread has allowed populations to communicate faster and effortlessly, to access internet, and it has accelerated social and economic development[3,4]. Widespread and increasing coverage of mobile phones also provides an opportunity to conduct mobile phone surveys (MPS). Some benefits of MPS include cost efficiency, shorter fieldwork duration due to reduced time devoted to travel, improved ability to supervise data collection through the recording of interviews and automated storage of paradata, and increased access to populations that are harder to reach[5,6]. MPS have already covered some health-related topics, including family planning and sexual and reproductive health[1,7], but few MPS have included questions to monitor trends in fertility and mortality.

In countries lacking comprehensive systems of civil registration and vital statistics, data on mortality and fertility are generally collected through face-to-face interviews in survey programs such as the Demographic and Health Surveys (DHS) or Multiple Indicator Cluster Surveys (MICS). However, face-to-face interviews might not be the most cost-effective approach and they are not suitable in settings affected by conflicts, disasters or epidemic outbreaks. During the Ebola epidemic in Liberia, MPS were used to estimate mortality both from Ebola and other causes based on reports of recent household deaths[8]. During the COVID-19 pandemic, MPS were proposed as a viable way to assess excess mortality without posing additional risks to survey teams and respondents[9,10]. MPS thus represent a promising alternative to collect demographic data.

For peer review only

MPS have several disadvantages that require careful consideration, such as higher non-response rates and shortened interview duration – requiring changes to the questionnaires used in face-to-face interviews[11]. Moreover, MPS may not be representative, especially when based on random digit dialing, as compared to a survey using phone numbers previously collected in face-to-face surveys[1]. This is because inequalities in use of digital technology, known as a "digital divide", persist. Mobile phone use is related to gender, socioeconomic status, technological proficiency and literacy. Women have been noted to be at a disadvantage in mobile phone use, although the more common mobile phones are, the smaller the gender gap is[12–14]. This gender divide in mobile phone use reflects multiple barriers women face, including lack of financial resources, lack of time due to care responsibilities or household chores, socio-cultural restrictions, and lower educational attainment[15]. Mobile phone users are typically also better educated[16] and younger[17]. Moreover, rural dwellers use mobile phones relatively less than urbanites[18], although overall access remains high in rural areas as well[19]. Other factors, not related directly to an individual's characteristics, also determine selectivity in mobile phone users. For example, in remote locations and near borders, cellular network coverage may be weak, leaving populations living in such areas under-represented in MPS.

At the aggregate level, use of mobile phone has been linked to sustainable development[2]. The higher the diffusion of mobile phones, the lower under-five (U5M) and maternal mortality, the greater the use of contraception, and the smaller ideal family size[2,20]. This is in part because mobile phone use is a good proxy for socioeconomic development, but also because mobile phone use can improve access to health services, including reproductive health and antenatal care[21–25]. Such benefits could hinder obtaining a representative sample, even after post-sampling weighting. For example, a study conducted in Burkina Faso found higher modern contraception use among

For peer review only

respondents reached by an MPS (40%) compared to respondents in a face-to-face survey (26%)[7]. This difference in modern contraceptive use persisted after post-stratification. Similarly, Lefevre and colleagues[26] explored variations in several reproductive maternal, newborn and child health indicators, such as antenatal and postnatal care, skilled attendance at birth, measles and tetanus vaccinations, using DHS surveys in 15 countries. Even after controlling for a wide range of sociodemographic variables, child health and natal care was better when mothers had a mobile phone. A notable exception was the adoption of initial, exclusive, and continuous breastfeeding, which was less common among mobile phone users.

These previous studies suggest that fertility and mortality estimates derived from MPS are likely to be biased, but this issue has not been systematically evaluated. In this study, we leverage existing DHS data containing information on mobile phone use by women to assess the selection bias in U5M and fertility estimates. We expect that these estimates are biased downwards, since mortality and fertility tend to be higher in lower socioeconomic groups[27,28]. We assess mobile phone use by distinguishing between women owning a mobile phone (henceforth referred to as 'ownership') and those who can potentially be reached through another household member who owns a phone (henceforth referred to as 'access'). We first identify correlates of mobile phone ownership and access through logistic regressions and establish through Poisson regressions whether ownership and access are associated with variations in U5M and fertility.

DATA AND METHODS

Data collection

We used all available DHS[29] that include information on mobile phone ownership and access. The question on mobile phone *ownership* (v169a) is included in the individual questionnaire, while the

For peer review only

question on *access* to a mobile phone (hv243a) is in the household questionnaire. Access to mobile phone means that the woman either owns a mobile phone or lives in a household where the household head owns a mobile phone. Some countries have more than one survey with such data, so in these cases, we used the most recent one. In total, we used 34 DHS, mostly from Sub-Saharan Africa (see Appendix Table 1 in the supplementary material). These surveys were collected between 2015 and 2021. All women have complete information for all variables of interest included in this study.

The survey India 2019-21 is excluded from the analysis as the information is missing for about 85% of respondents. In Papua New Guinea 2016-18 information is missing for 0.4% of respondents, in Indonesia 2017 for 0.1%, and in Pakistan 2017-18 for 0.01%. We removed the observations with missing data from these three samples.

Data analysis

Using data from the birth histories administered for women aged 15–49, we computed total fertility rates (TFR) for the 36-month period preceding the survey and under-five mortality rates (U5MR) for the 10-year period preceding the survey. We used Poisson regressions to estimate the association between mobile phone ownership and access and fertility and U5M rates. For fertility, the outcome variable was the number of births, while for U5M, it was the number of deaths before age 5. We conducted two sets of regressions. In the first set — unadjusted estimates —, we regressed the outcome variable on the ownership of a mobile phone (model 1) or access to a mobile phone in the household (model 2), including covariates for the mother's age group when estimating fertility, and the child's age when estimating mortality. In the second set — adjusted estimates — , we added educational level, place of residence, wealth, and marital status as independent variables

For peer review only

to models 1 and 2. As for the categories of these independent variables, place of residence has two values: urban and rural; educational level takes three values: no education, primary education, and secondary and higher education; wealth has two values: poor (wealth quintiles 1 and 2) and not poor (wealth quintiles 3, 4 and 5); and, marital status takes two values: not in union (never married, not living together, divorced, or widowed) and in union (married or living together). We also included exposure (person-years calculated for women for fertility and for children for mortality) as an offset in all models. In addition, we computed raking weights to match the marginal distributions of the same sociodemographic variables and examine whether potential estimation biases could be addressed using post-stratification.

Differences in U5MR and TFR could be due to differences in women's behavior, which could be captured by mobile phone use, but not by sociodemographic variables. We proposed a series of logistic regressions to analyze the association between mobile phone ownership and access, and some variables connected to U5M and fertility. For mortality, we used six outcome variables. First, we modeled the likelihood of a mother having at least 4 antenatal care visits among those who gave birth in the 5 years before each survey. Second, the likelihood of a newborn receiving at least two signal functions as part of postnatal care (among births that occurred in the last 24 months). Third, the likelihood of a child being exclusively breastfed for the first 6 months (among the most recent birth in the last 6 months). Fourth, the likelihood of a child receiving eight basic immunizations among children aged 12 to 35 months at the time of the survey. The eight basic immunizations are one dose of BCG, three doses of DPT, three doses of Polio (excluding at birth), and one dose of Measles. Fifth, we analyzed the likelihood of skilled attendance at birth, which includes the presence of a doctor, nurse or nurse-midwife during delivery. All births in the 5-year period before the survey are considered. The last outcome variable connected with U5M that was analyzed was

For peer review only

the prevalence of underweight among children aged 0–5 at the time of the survey. A child is considered as being underweight if the weight-for-age z-score is below two standard deviations below the mean on the WHO Child Growth Standards. On the other hand, for fertility we used four outcome variables. First, the use of modern contraceptives. Second, sexual activity. We considered a woman to be sexually active when she reported having had sexual intercourse in the last month or being pregnant. Lastly, we analyzed duration of postpartum amenorrhea and duration of postpartum abstinence. For the latter two variables, we used a linear model since duration is measured as the number of months. Similar to the estimates of under-five fertility and mortality rates, unadjusted and adjusted models were estimated for all outcome variables.

We conducted this analysis using the programming language for statistical computing and graphics `R`[30]. We used the following packages: `rdhs`[31] for downloading the DHS datasets, `tidyverse`[32] for data manipulation and graphics, `demogsurv`[33] for preparing person-period tables stratified by the independent variables, `survey`[34] and `broom`[35] for econometric estimations and visualization, `anesrake`[36] for computing raking weights to match the marginal distributions of sociodemographic variables for testing for post-stratification, `MetBrewer`[37] for color palettes in our plots, and `kableExtra`[38] and `knitr`[39] for writing and editing the manuscript.

Patient and public involvement

Patients and/or the public were not involved in the design, or conduct, or reporting, or dissemination plans of this research.

For peer review only

RESULTS

Across the 34 surveys used, the percentage of women owning a mobile phone ranges from 23.6% in Burundi to 96.7% in Armenia. The percentage of women who have access to a mobile phone ranges from 55% in Burundi to 100% in the Maldives (see Appendix Table 1). The median ratio of the TFR calculated among non-owners to the TFR calculated among mobile phone owners is 1.48. Fertility is always higher among non-owners, with the lowest ratio at 1.04 in Bangladesh and the highest at 1.93 in Haiti. In absolute terms, these differences correspond to more than one child per woman in 25 of the 34 surveys studied here. Similarly, U5M is higher when calculated among non-owners, with the exception of South Africa 2016 and Zambia 2018-19. The median ratio of U5M calculated from reports of non-owners to that calculated from mothers with mobile phones is 1.29, and it ranges from 0.85 in South Africa to 2.5 in Albania. Differences in TFR and U5MR remain when access to a mobile phone is considered, but they are slightly reduced.

A series of logistic regressions (see Appendix Figures 1 to 5 in the supplementary material) conducted for each survey separately indicate that women with mobile phones differ from non-owners by age group, educational level, area of residence, wealth quintile, and marital status. Except for age, differences do not disappear when access to a mobile phone instead of ownership is considered. Compared to young adults aged 20–24, adolescents aged 15–19 are less likely to own a phone in all countries, although this is not true in 27 countries when we consider access rather than ownership. Age patterns of phone ownership vary by country for older women, but in most cases, differences are not statistically significant when access is considered.

Compared to women without education, women with primary education are significantly more likely to own a mobile phone in 30 surveys and women with secondary or higher education are

For peer review only

significantly more likely to own a mobile phone in all surveys. The number of surveys with a significant difference decreases, respectively, to 28 surveys and 30 surveys when access is considered. Women living in urban areas are significantly more likely to own a mobile phone in 29 surveys and to have access in 19 surveys. Odds ratios for women who own a mobile phone range from 0.94 (95% CI: 0.74 – 1.19) in South Africa to 5.42 (95% CI: 4.23 – 6.95) in Ethiopia, while for access, from 0.41 (95% CI: 0.25 – 0.67) in South Africa to 4.2 (95% CI: 3.12 – 5.67) in Sierra Leone.

In terms of household assets, the gaps are very large between women in the poorest households (quintiles 1 to 2) and those living in the most affluent households (quintiles 3 to 5). Unsurprisingly, women living in households with more amenities are between 1.17 (Maldives, 95% CI: 0.83 – 1.66) times and 4.85 (Burundi, 95% CI: 4.24 – 5.55) times more likely to own a mobile phone. As for access, the odds ratios range between 2.7 (Mali, 95% CI: 2.03 – 3.58) and 24.37 (Indonesia, 95% CI: 18.01 – 32.97). Women not in union are more likely to own a mobile phone than women in union in 19 countries, with odds ratios ranging from 0.56 (95% CI: 0.48 – 0.64) in Nepal to 1.78 (95% CI: 1.4 – 2.26) in the Philippines.

Figures 1 and 2 present the exponentiated coefficients of mobile phone ownership and access from Poisson regressions on U5M and fertility, respectively. Left panels of both figures show unadjusted estimates, while right panels contain adjusted estimates. Unadjusted coefficients suggest that in most countries, ownership is associated with lower odds of experiencing a birth or the death of a child under five years of age. When considering U5M estimates and mobile phone ownership, coefficients are statistically significant in all countries but nine (left panel of Figure 1). However, in most countries differences by ownership are no longer significant once we adjust for marital status, education, wealth, and place of residence (right panel of Figure 1). Women who own a

For peer review only

mobile phone are less likely to lose a child in only four countries (Cameroon, Gambia, Nepal, and Nigeria) after adjusting for sociodemographic characteristics. Overall, U5M is not statistically associated with owning or having access to a mobile phone, after controlling for basic sociodemographic characteristics. U5MR estimates obtained using post-stratification weights from mobile phone owners are in line with these findings (see right panel of Figure 3). Only in Nigeria is it not possible to reproduce actual U5MR using ex-post weightings. Results for access are presented in Appendix Figure 6.

Unadjusted coefficients from the Poisson regressions on fertility for mobile phone owners are also statistically significant in 30 countries (left panel of Figure 2). However, in contrast to what we observed for U5M, the negative effect of owning a phone persists for fertility in most countries even after controlling for covariates (right panel of Figure 2). Ethiopia is the only survey where this association loses significance after controlling for sociodemographic characteristics. When access is considered, the effect is not significant in five additional countries (Burundi, Cameroon, Mali, Pakistan, and Senegal). In most countries, ownership or access is associated with lower fertility, even after controlling for basic sociodemographic characteristics. The use of post-stratification weights based on the marginal distribution of sociodemographic variables of mobile phone owners shows that it is possible to reproduce the observed TFR in a total of 22 countries (see left panel of Figure 3). However, this also means that post-stratification cannot provide unbiased fertility estimates in one-third of the countries included in our study. When access is considered (see Appendix Figure 6), there are no statistical differences with the TFRs observed in the general population. As mobile phone access is very high, marginal distributions of the sociodemographic variables of women with access are very similar to that of the whole population so post-stratification plays a small role in this case.

For peer review only

In Figure 4, we present the estimates of the association between ownership and variables connected to fertility. Results for access to mobile phones are presented in Appendix Figure 7. We observe statistically significant differences in use of modern contraceptives in 23 surveys. In most of them, owners are more likely to use contraceptives. After controlling for covariates, differences remain in 20 surveys. When access is considered, the number of surveys is reduced to 16. In contrast, mobile phone owners are less likely to be sexually active in 14 surveys, even after controlling for sociodemographic characteristics. However, differences disappear in most surveys when access is considered. Postpartum amenorrhea periods are significantly shorter among owners in 19 surveys, although only in 8 surveys after including controls. In contrast, owners have significantly longer postpartum abstinence periods in 15 surveys, but only in 8 surveys after adjusting for controls.

Analyses of the association between ownership and variables connected to U5M are presented in Figure 5. Results for access are in Appendix Figure 8. Owners are more likely to have had at least four antenatal care visits in all but 3 surveys. Differences are not statistically significant in 6 surveys after controlling for covariates. Likewise, births born to mothers who own a mobile phone are more likely to have received at least two signal functions as part of postnatal check-ups in 28 surveys, although the number of surveys is reduced to 18 after controlling for covariates. In contrast, owners are more likely to exclusively breastfeed for the first 6 months to newborns in 4 surveys, and less likely in 4 other surveys, although these differences disappear in virtually all surveys after controlling for covariates. Children born to owners are more likely to have received basic immunization in only 5 surveys after adjusting for controls. In contrast, children born to owners are more likely to have been attended by health professionals at delivery in 27 surveys. Owners are less likely to have underweight children in more than half of the surveys, even after controlling for

For peer review only

covariates. Differences disappear for most variables when access is considered. Exceptions are antenatal care, the presence of a health professional at delivery, and, to a lesser extent, underweight.

CONCLUSIONS

We analyzed the social gradient in mobile phone ownership and access in 34 LMIC, and evaluated the implications for fertility and U5M estimates derived from MPS. Consistent with previous research[16–19], we found that well educated, better-off, and urban women are more likely to own and have access to a mobile phone. These differences in the use of mobile phones could bias demographic estimates from MPS.

Fertility and U5M are higher in less advantaged populations[27,28], groups which are underrepresented in MPS. Therefore, MPS are likely to underestimate true levels of fertility and U5M in the wider population. This is an important first conclusion of this study: MPS will underestimate U5M and total fertility. However, the negative association between U5M and mobile phone ownership disappears once we control for a number of sociodemographic characteristics that can be easily collected in MPS. This is a second, and methodologically, important finding of our study as it suggests that any downward bias in U5M estimates from MPS due to selectivity of respondents can be corrected using a post-stratification procedure. More substantively, our results contradict the hypothesis that at the aggregate level the expansion of mobile phones is associated with lower child mortality as this association seems spurious[2].

Surprisingly, the negative association between mobile phone ownership and fertility persists in many of the countries included in this study even after controlling for sociodemographic characteristics. From a methodological standpoint, this suggest that fertility rates could be biased downwards if they are estimated from MPS, even after post-stratification. This third important

For peer review only

finding is consistent with previous research arguing that mobile phone use is associated with higher rates of contraceptive use[2,7,20]. Our results suggest that the use of modern contraceptives is more likely among owners in two out of five surveys analyzed in this study, even after controlling for wealth, education, place of residence, marital status and age. Further, there are some apps that track menstrual periods and send alerts on ovulation days, allowing for better birth control[2,20]. Even less sophisticated models of mobile phones — i.e., not smartphones — include calendars that can be used to schedule reminders for tracking the menstrual cycle or taking contraceptives. In addition, we found that mobile phone owners are less likely to be sexually active in most of the surveys. Higher odds of contraceptive use and lower odds of sexual activity lead to lower fertility rates. It is worth noting that one difference between fertility and mortality is that fertility outcomes can be more easily controlled based on preferences than exposure to illness and injuries. These fertility preferences may be correlated with, but are not fully captured by, the sociodemographic characteristics.

Our results also show that there are no significant differences between owners and non-owners in terms of exclusive breastfeeding. This does not contradict what Lefevre and colleagues[26] found, as they estimated their results using differences in means without controlling for covariates while we have used the association of exclusive breastfeeding conditional on mobile phone ownership and other sociodemographic variables. Our findings can help to explain why the differences in U5MR by ownership disappear when controlling for covariates, since the health benefits from exclusive breastfeeding and basic immunization could be offsetting the negative effects of no or few prenatal and postnatal checkups, or low birth weight.

As for logistical implications for fieldwork, MPS should preferably be conducted at times when household heads are likely to be present so that women in the household who do not own a mobile

For peer review only

phone can be surveyed. But biases related to the selection of phone users may remain even after using this approach. A different way of accounting for selection bias (apart from post-stratification), is to make use of sampling quotas (i.e., pre-stratification). This is usually based on a set of simple screening questions like age, rural/urban residence and educational attainment. However, pre-stratification might be more expensive and it will require greater effort to reach respondents with the desired attributes. Finally, from the perspective of survey designers, conducting complementary face-to-face surveys could be suitable for reaching populations that are typically difficult to cover through MPS. It should be noted that our study evaluates selection bias due to sampling frame limitations (mobile phone access/ownership) and not due to non-response. Non-response is often high in MPS, and considerably higher than in face-to-face surveys. We did not consider either the recall errors which could be more systematic in MPS due to lower engagement of the respondents.

In conclusion, while MPS are a cheaper alternative or complement to classical face-to-face surveys for estimating demographic and health indicators, estimates derived from MPS cannot be taken at face value owing to the unequal ownership and access of mobile phones in many LMIC. In this study, we have shown that under-five mortality estimates can be corrected using a post-stratification on a number of sociodemographic background characteristics to match nationally representative estimates. Adjusting fertility estimates is less straightforward, most likely because fertility decision making is an expression of individual preferences in addition to background characteristics and living conditions.

For peer review only

Contributors

DASP and BM led the conception and design of the study. DASP and BM analyzed the data, produced the estimates and drafted the manuscript. AMO, JB and GR reviewed the estimates and helped with the interpretation of the results. All authors critically reviewed the manuscript and approved the final version of the paper. DASP is the guarantor of the overall content of this manuscript and had final responsibility for the decision to submit for publication.

Competing interests

We declare no competing interests.

Funding

This study was supported by the Bill & Melinda Gates Foundation grant number INV-023211. The funders of the study had no role in the study design, data analysis, data interpretation, or writing of the report.

Data availability statement

All datasets are publicly and freely available on the DHS Program website. https://dhsprogram.com/data/

Patient consent for publication

Not applicable.

Ethics Approval

Not applicable since the study was a secondary analysis of publicly available data.

For peer review only

References

1 Gibson DG, Pereira A, Farrenkopf BA, Labrique AB, Pariyo GW, Hyder AA. Mobile phone surveys for collecting population-level estimates in low-and middle-income countries: A literature review. *Journal of Medical Internet Research* 2017; **19**: e139.

2 Rotondi V, Kashyap R, Pesando LM, Spinelli S, Billari FC. Leveraging mobile phones to attain sustainable development. *Proceedings of the National Academy of Sciences* 2020; **117**: 13413–20.

3 Donner J. The use of mobile phones by microentrepreneurs in Kigali, Rwanda: Changes to social and business networks. *Information Technologies and International Development* 2007; **3**: 3–19.

4 Jagun A, Heeks R, Whalley J. The impact of mobile telephony on developing country micro-enterprise: A Nigerian case study. *Information Technologies and International Development* 2008; **4**: 47–65.

5 Firchow P, Ginty RM. Including hard-to-access populations using mobile phone surveys and participatory indicators. *Sociological Methods & Research* 2020; **49**: 133–60.

6 Kempf AM, Remington PL. New challenges for telephone survey research in the twenty-first century. *Annual Review of Public Health* 2007; **28**: 113–26.

7 Greenleaf AR, Gadiaga A, Guiella G, *et al.* Comparability of modern contraceptive use estimates between a face-to-face survey and a cellphone survey among women in Burkina Faso. *PLOS ONE* 2020; **15**: e0231819.

8 Kuehne A, Lynch E, Marshall E, *et al.* Mortality, morbidity and health-seeking behaviour during the Ebola epidemic 2014–2015 in Monrovia results from a mobile phone survey. *PLoS Neglected Tropical Diseases* 2016; **10**: 1–2.

9 Adjiwanou V, Alam N, Alkema L, *et al.* Measuring excess mortality during the COVID-19 pandemic in low- and lower-middle income countries: The need for mobile phone surveys. 2020. DOI:10.31235/osf.io/4bu3q.

10 Chasukwa M, Choko AT, Muthema F, *et al.* Collecting mortality data via mobile phone surveys: A non-inferiority randomized trial in Malawi. *PLOS Global Public Health* 2022; **2**: e0000852.

11 Dabalen A, Etang A, Hoogeveen J, Mushi E, Schipper Y, Engelhardt J von. Mobile phone panel surveys in developing countries: A practical guide for microdata collection. Washington, DC: World Bank, 2016.

12 Blumenstock JE, Hall MG, Eagle N. Divided we call: Disparities in access and use of mobile phones in Rwanda. *Information Technologies and International Development* 2012; **8**: 1–6.

For peer review only

13 Huyer S, Hafkin N, Ertl H, Dryburgh H. Women in the Information Society. In: The digital divide to digital opportunities: Measuring infostates for development. NRC Press, 2005: 135–96.

14 Murphy LL, Priebe AE. "My co-wife can borrow my mobile phone!": Gendered geographies of cell phone usage and significance for rural Kenyans. *Gender, Technology and Development* 2011; **15**: 1–23.

15 Huyer S, Carr M. Information and Communication Technologies: A priority for women. *Gender, Technology and Development* 2002; **6**: 85–100.

16 L'Engle K, Sefa E, Adimazoya EA, *et al.* Survey research with a random digit dial national mobile phone sample in Ghana: Methods and sample quality. *PLoS ONE* 2018; **13**: e0190902.

17 Aker JC, Mbiti IM. Mobile phones and economic development in Africa. *Journal of Economic Perspectives* 2010; **24**: 207–32.

18 Wesolowski A, Eagle N, Noor AM, Snow RW, Buckee CO. Heterogeneous mobile phone ownership and usage patterns in Kenya. *PLos ONE* 2012; **7**: e35319.

19 Wakadha H, Chandir S, Victor E, *et al.* The feasibility of using mobile-phone based SMS reminders and conditional cash transfers to improve timely immunization in rural Kenya. *Vaccine* 2013; **31**: 987–93.

20 Billari F, Rotondi V, Trinitapoli J. Mobile phones, digital inequality and fertility: Longitudinal evidence from Malawi. *Demographic Research* 2020; **42**: 1057–96.

21 Smith C, Gold J, D Ngo T, Sumpter C, Free C. Mobile phone-based interventions for improving contraception use. London: Cochrane Database of Systematic Reviews, 2015.

22 Coleman J, Bohlin KC, Thorson A, *et al.* Effectiveness of an SMS-based maternal mHealth intervention to improve clinical outcomes of HIV-positive pregnant women. *AIDS Care* 2017; **29**: 890–7.

23 Hall CS, Fottrell E, Wilkinson S, Byass P. Assessing the impact of mHealth interventions in low- and middle-income countries - what has been shown to work? *Global Health Action* 2014; **7**. DOI:10.3402/gha.v7.25606.

24 Lund S, Nielsen BB, Hemed M, *et al.* Mobile phones improve antenatal care attendance in Zanzibar: A cluster randomized controlled trial. *BMC Pregnancy and Childbirth* 2014; **14**: 1–0.

25 Noordam AC, Kuepper BM, Stekelenburg J, Milen A. Improvement of maternal health services through the use of mobile phones. *Tropical Medicine and International Health* 2011; **16**: 622–6.

26 LeFevre AE, Shah N, Harrisson Bashingwa JJ, George AS, Mohan D. Does women's mobile phone ownership matter for health? Evidence from15 countries. *BMJ Global Health* 2020; **5**: e002524.

For peer review only

27 Finlay JE, Mejía-Guevara I, Akachi Y. Inequality in total fertility rates and the proximate determinants of fertility in 21 sub-Saharan African countries. *PLoS ONE* 2018; **13**: e0203344.

28 Rogers RG, Wofford S. Life expectancy in less developed countries: Socioeconomic development or public health? *Journal of Biosocial Science* 2008; **21**: 245–52.

[dataset] 29 ICF. Data from: Demographic and Health Surveys. The DHS Program, 2022. http://www.dhsprogram.com/Data/.

30 R Core Team. R: A language and environment for statistical computing. R version 4.2.1 patched. Vienna, Austria: R Foundation for Statistical Computing, 2022 https://www.R-project.org/.

31 Watson OJ, FitzJohn R, Eaton JW. Rdhs: An R package to interact with the Demographic and Health Surveys (DHS) Program datasets. *Wellcome Open Research* 2019; **4**: 103–25.

32 Wickham H, Averick M, Bryan J, *et al.* Welcome to the Tidyverse. *Journal of Open Source Software* 2019; **4**: 1686–91.

33 Eaton J, Masquelier B. Demogsurv: Demographic analysis of DHS and other household surveys. R package version 0.2.5. 2021 https://github.com/mrc-ide/demogsurv.

34 Lumley T. Analysis of complex survey samples. *Journal of Statistical Software* 2004; **9**: 1–9.

35 Robinson D, Hayes A, Couch S. broom: Convert statistical analysis objects into tidy tibbles. R package version 0.7.12. 2022 https://CRAN.R-project.org/package=broom.

36 Pasek J. Anesrake: ANES Raking Implementation. R package version 0.80. 2018 https://CRAN.R-project.org/package=anesrake.

37 Mills BR. MetBrewer: Color palettes inspired by works at the Metropolitan Museum of Art. R package version 0.2.0. 2022 https://CRAN.R-project.org/package=MetBrewer.

38 Zhu H. kableExtra: Construct complex table with 'kable' and pipe syntax. R package version 1.3.4. 2021 https://CRAN.R-project.org/package=kableExtra.

39 Xie Y. Knitr: A general-purpose package for dynamic report generation in R. R package version 1.37. 2021 https://yihui.org/knitr/.

For peer review only

Figure 1: Unadjusted and adjusted estimates of the effect of owning and having access to a mobile phone on under-five mortality from Poisson regressions.

Figure 2: Unadjusted and adjusted estimates of the effect of owning and having access to a mobile phone on fertility from Poisson regressions.

Figure 3: Under-five mortality rate and total fertility rate estimates testing for post-stratification weights from women who own a mobile phone.

Figure 4: Estimates of the difference in current use of modern contraceptives (MCU), sexual activity (SexAct), duration of postpartum amenorrhea (Amen), and duration of postpartum abstinence (Abst), between owners and non-owners of a mobile phone.

Figure 5: Estimates of the difference in exclusive breastfeeding (BrFeed), births who had at least 4 antenatal care visits (ANC), newborns who received at least 2 signal functions (PNC), children who received basic immunization (Vax), births delivered by skilled professionals (Skill), children who are underweight (UnderW), between owners and non-owners of a mobile phone.

For peer review only

Figure 1: Unadjusted and adjusted estimates of the effect of owning and having access to a mobile phone on under-five mortality from Poisson regressions.

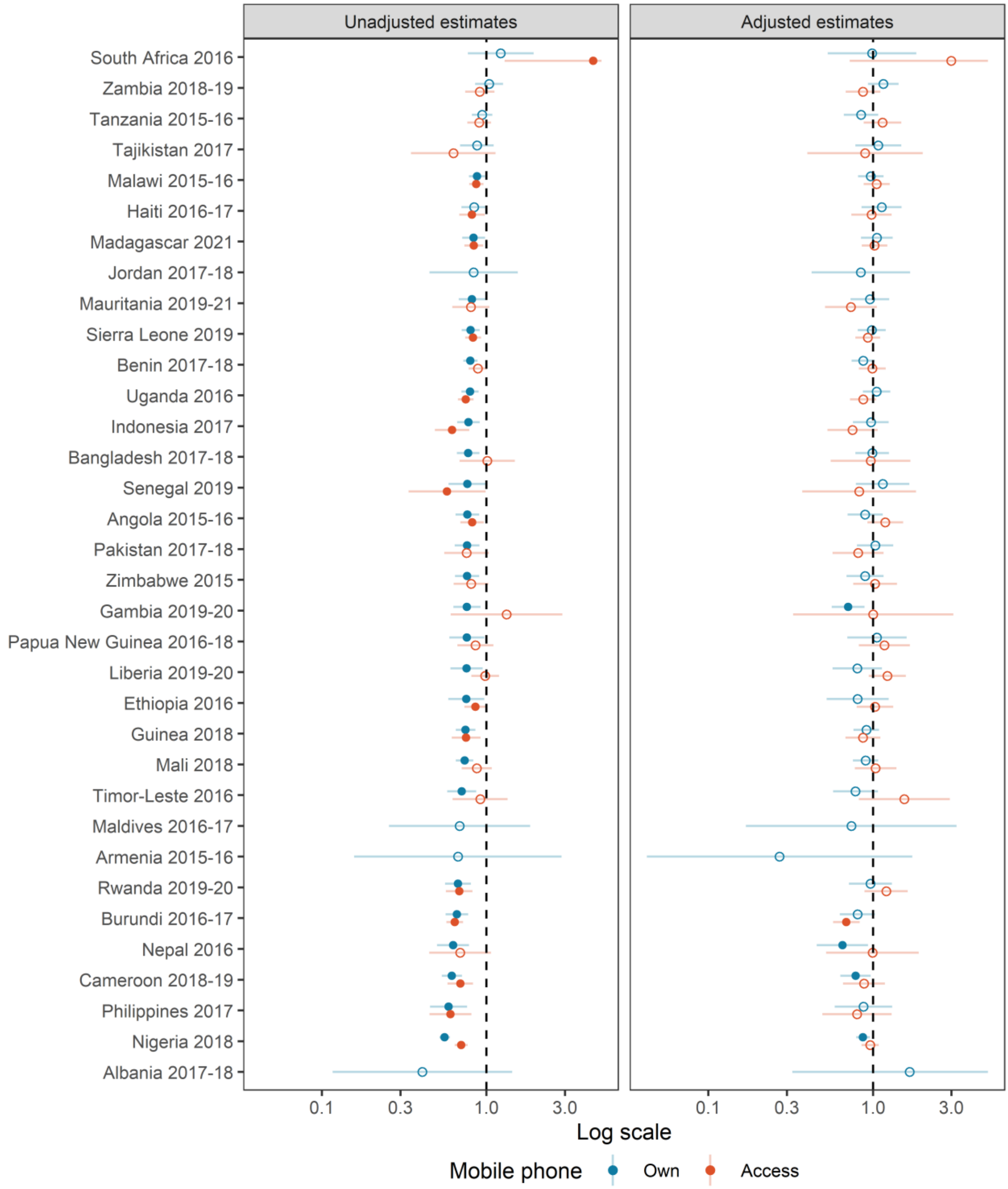

Note: Estimates for access to a mobile phone are not presented for Albania, Armenia, Jordan and Maldives, as access is almost 100%. Fill dots mean that estimates are statistically significant at the 95% confidence level, while empty dots mean that estimates are not statistically significant.

Figure 2: Unadjusted and adjusted estimates of the effect of owning and having access to a mobile phone on fertility from Poisson regressions.

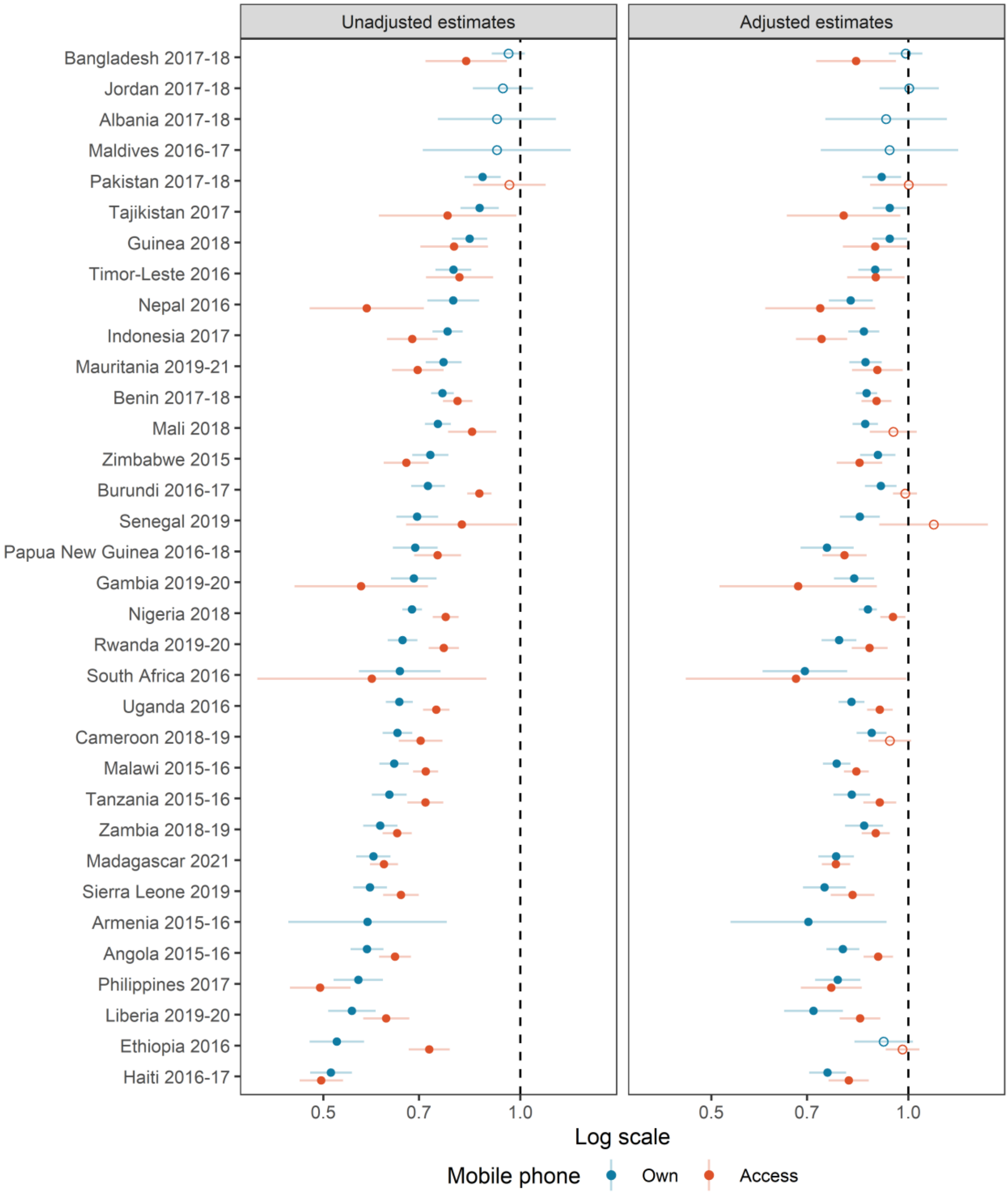

Note: Estimates for access to a mobile phone are not presented for Albania, Armenia, Jordan and Maldives, as access is almost 100%. Fill dots mean that estimates are statistically significant at the 95% confidence level, while empty dots mean that estimates are not statistically significant.

Figure 3: Under-five mortality rate and total fertility rate estimates testing for post-stratification weights from women who own a mobile phone.

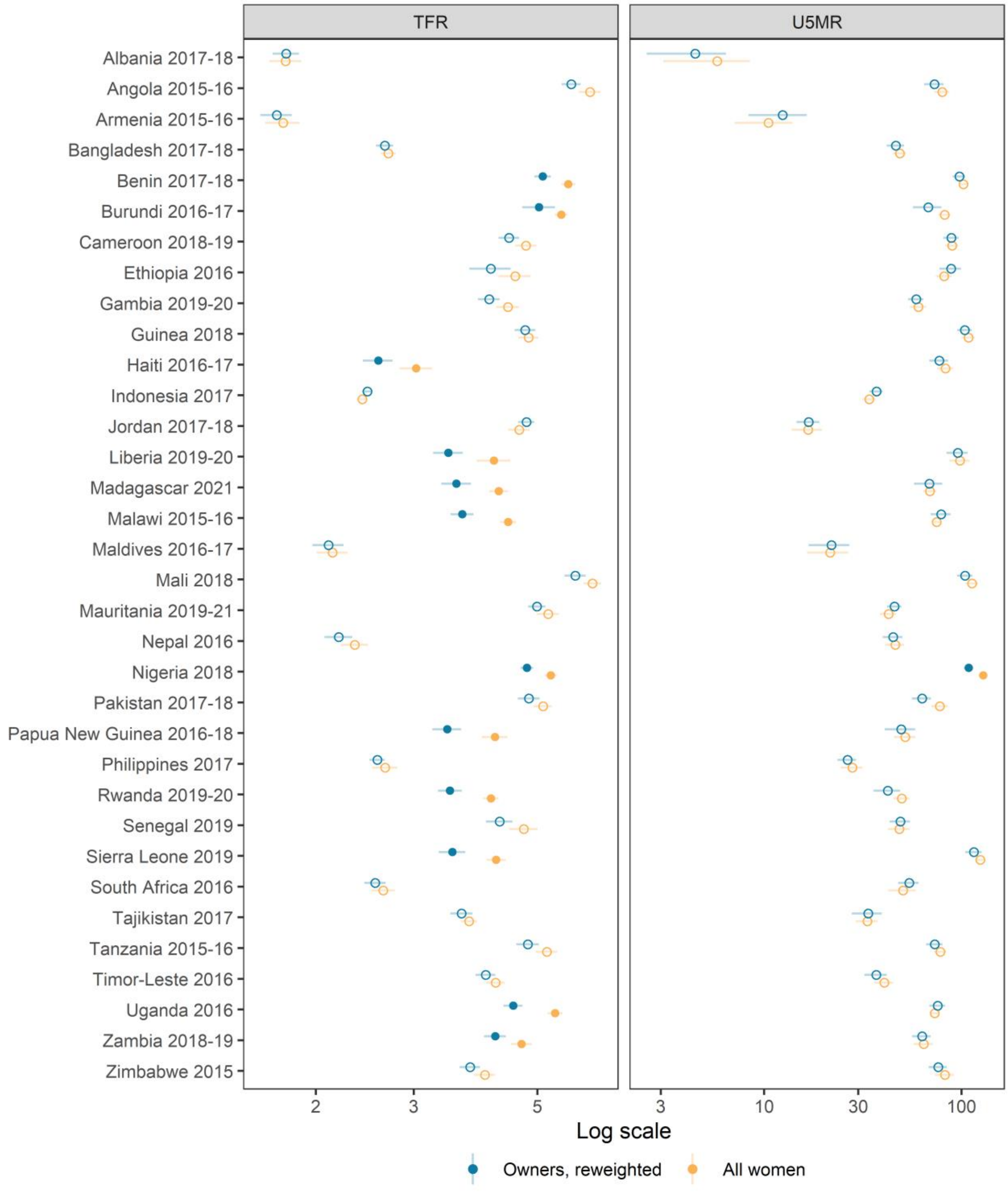

Note: Fill dots mean that the difference between the actual rate and the re-weighted rate is statistically significant at the 95% confidence level, while empty dots mean that the difference is not statistically significant.

Figure 4: Estimates of the difference in current use of modern contraceptives (MCU), sexual activity (SexAct), duration of postpartum amenorrhea (Amen), and duration of postpartum abstinence (Abst), between owners and non-owners of a mobile phone.

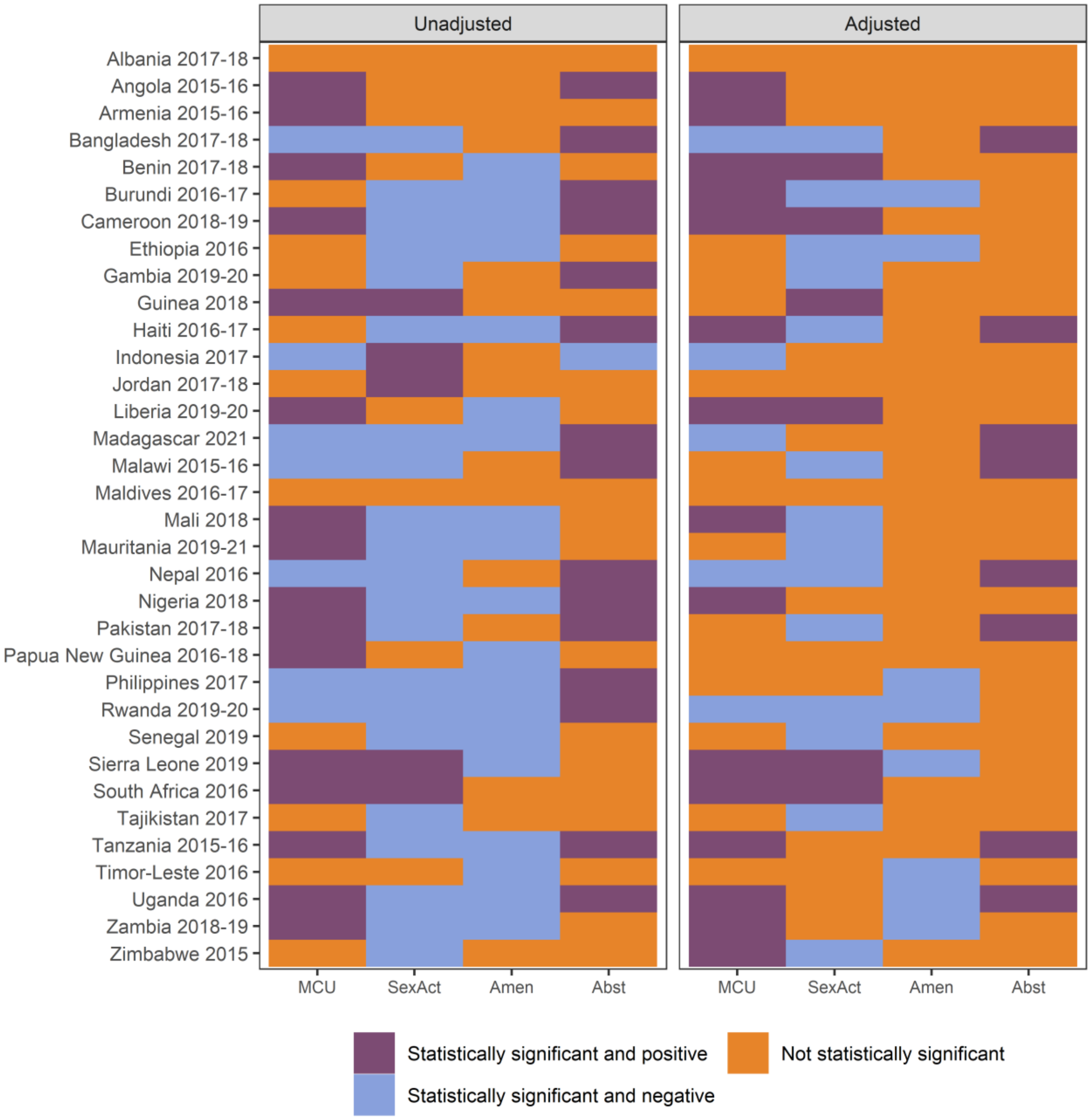

Figure 5: Estimates of the difference in exclusive breastfeeding (BrFeed), births who had at least 4 antenatal care visits (ANC), newborns who received at least 2 signal functions (PNC), children who received basic immunization (Vax), births delivered by skilled professionals (Skill), children who are underweight (UnderW), between owners and non-owners of a mobile phone.

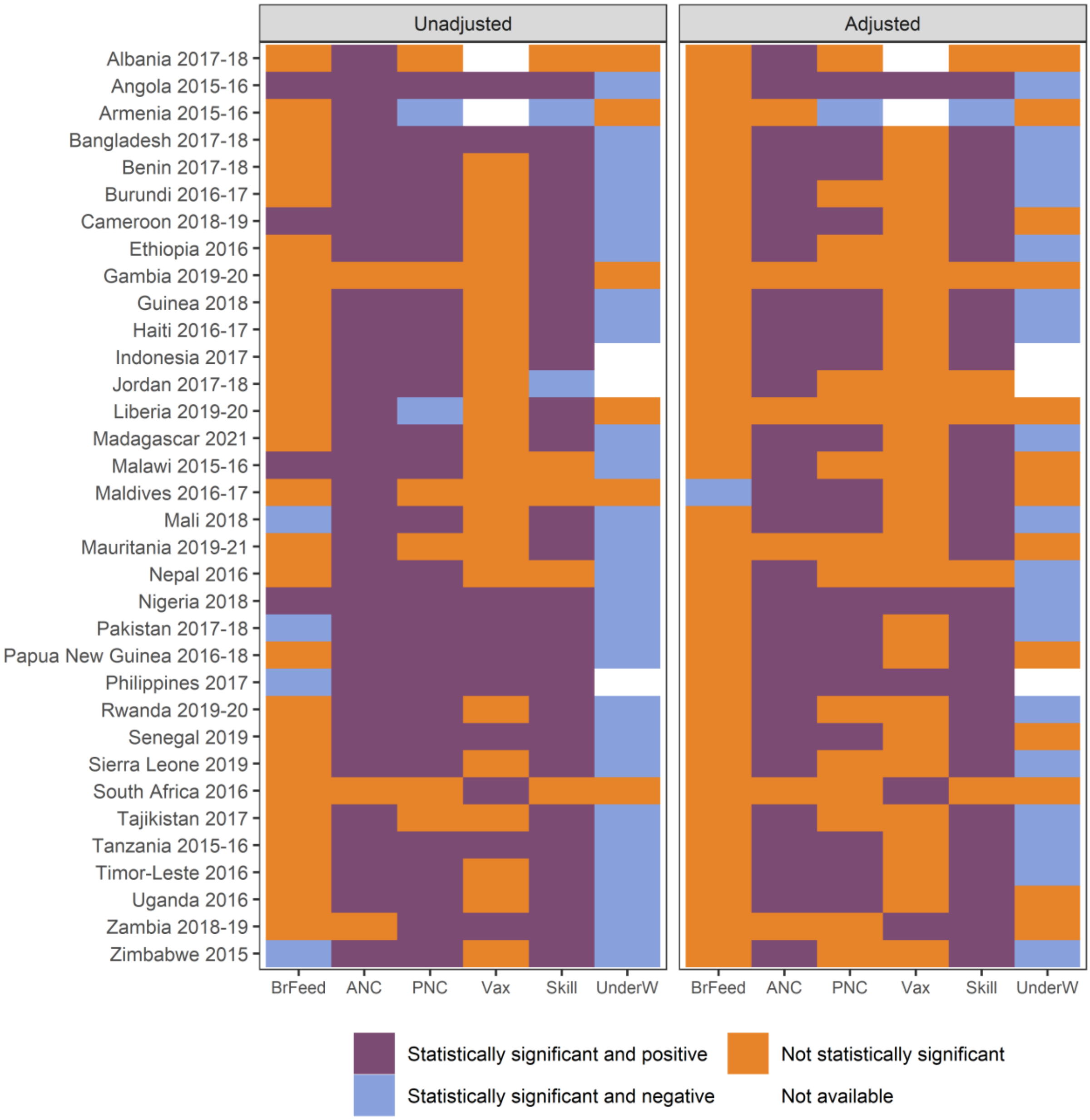

Appendix Table 1: DHS including information on mobile phone ownership and access, and total fertility rate (TFR) for the 3-year period before the survey and under-five mortality rate (U5MR) for the 10-year period before the survey by mobile phone status.

		Mobile phone ownership					Access to a mobile phone				
		Owns a mobile phone			Does not own a mobile phone		Can access a mobile phone			Cannot access a mobile phone	
Survey	Sample size	% of women	TFR	U5MR	TFR	U5MR	% of women	TFR	U5MR	TFR	U5MR
Albania 2017-18	10,970	90.1	1.8	5.1	2.0	12.7	99.3	1.8	5.7	3.0	18.8
Angola 2015-16	14,379	51.2	4.7	67.9	8.0	89.1	75.3	5.5	74.0	8.4	92.3
Armenia 2015-16	6,116	96.7	1.7	10.3	3.0	15.5	99.8	1.7	10.5	*NA*	*NA*
Bangladesh 2017-18	20,127	60.2	2.2	43.9	2.3	56.6	97.2	2.2	48.7	2.7	46.7
Benin 2017-18	15,928	51.1	5.0	91.5	6.7	113.2	90.4	5.5	100.0	7.1	117.0
Burundi 2016-17	17,269	23.6	4.4	58.8	5.9	88.3	55.0	5.3	65.3	5.9	99.0
Cameroon 2018-19	13,901	63.2	4.0	72.1	6.4	115.4	91.5	4.6	85.0	6.0	126.6
Ethiopia 2016	15,683	27.3	2.8	65.8	5.2	84.1	62.1	4.0	74.9	5.5	88.7
Gambia 2019-20	11,865	76.4	4.1	56.0	6.1	75.2	99.6	4.4	60.3	8.1	69.3
Guinea 2018	10,874	68.9	4.6	98.7	5.5	129.6	94.1	4.8	105.9	6.0	141.9
Haiti 2016-17	14,371	57.1	2.3	75.9	4.4	89.8	86.3	2.7	78.1	5.3	98.9
Indonesia 2017	49,569	78.5	2.3	31.9	3.2	40.5	96.3	2.4	33.1	3.7	49.8
Jordan 2017-18	14,689	91.8	2.7	16.5	2.9	19.2	99.2	2.7	16.8	3.0	7.9
Liberia 2019-20	8,065	46.7	2.9	84.0	5.4	107.9	78.6	3.7	96.8	5.9	101.6
Madagascar 2021	18,869	34.6	3.0	60.1	5.0	72.5	61.3	3.2	63.7	5.0	71.7

For peer review only

Malawi 2015-16	24,562	32.8	3.2	67.7	5.1	77.9	62.4	3.8	69.9	5.4	81.4
Maldives 2016-17	7,699	95.5	2.1	21.5	2.4	27.7	100.0	2.1	21.6	*NA*	*NA*
Mali 2018	10,519	58.3	5.5	97.1	7.4	130.1	94.3	6.2	111.8	7.4	126.3
Mauritania 2019-21	15,714	76.7	4.9	40.6	6.8	49.5	96.1	5.1	41.7	7.5	57.7
Nepal 2016	12,862	72.6	2.2	40.3	3.1	62.8	97.3	2.3	45.3	4.0	64.9
Nigeria 2018	41,821	55.3	4.4	93.0	6.5	163.6	92.6	5.2	123.3	6.6	181.5
Pakistan 2017-18	12,363	39.2	3.2	64.4	3.8	85.1	95.4	3.5	77.0	4.1	88.8
Papua New Guinea 2016-18	15,141	34.3	3.2	40.6	4.7	56.2	66.6	3.7	48.5	5.1	56.4
Philippines 2017	25,074	85.8	2.5	24.6	4.3	41.4	95.5	2.5	27.0	5.2	36.8
Rwanda 2019-20	14,634	47.9	3.4	38.6	5.1	59.3	79.1	3.9	44.4	5.1	64.4
Senegal 2019	8,649	69.7	4.2	44.3	6.3	57.1	98.9	4.7	47.5	5.9	98.7
Sierra Leone 2019	15,574	42.6	3.1	110.3	5.2	132.6	81.4	3.9	118.2	5.9	142.0
South Africa 2016	8,514	91.2	2.6	51.2	4.1	43.7	98.8	2.6	51.2	4.1	10.5
Tajikistan 2017	10,718	53.7	3.5	31.5	4.1	35.6	98.1	3.7	32.6	5.1	64.4
Tanzania 2015-16	13,266	52.2	4.1	75.4	6.7	80.8	86.2	4.9	75.6	6.8	88.8
Timor-Leste 2016	12,607	65.6	3.9	35.5	5.0	49.4	94.3	4.2	40.8	5.1	38.1
Uganda 2016	18,506	45.5	4.3	65.2	6.7	79.7	81.8	5.1	68.6	6.8	89.7
Zambia 2018-19	13,683	53.0	3.7	65.4	6.1	63.4	81.2	4.2	62.7	6.6	69.1
Zimbabwe 2015	9,955	69.5	3.7	75.6	5.1	99.3	92.2	3.9	79.8	5.8	102.2

Note: Fertility and mortality rates for women who cannot access a mobile phone cannot be computed for Armenia and Maldives due to the small sample size.

For peer review only

Appendix Figure 1.1: Effect of the 15-19 age group on the odds of owning and having access to a mobile phone from binomial logistic regressions (20-24 age group is the reference). X-axis in log scale.

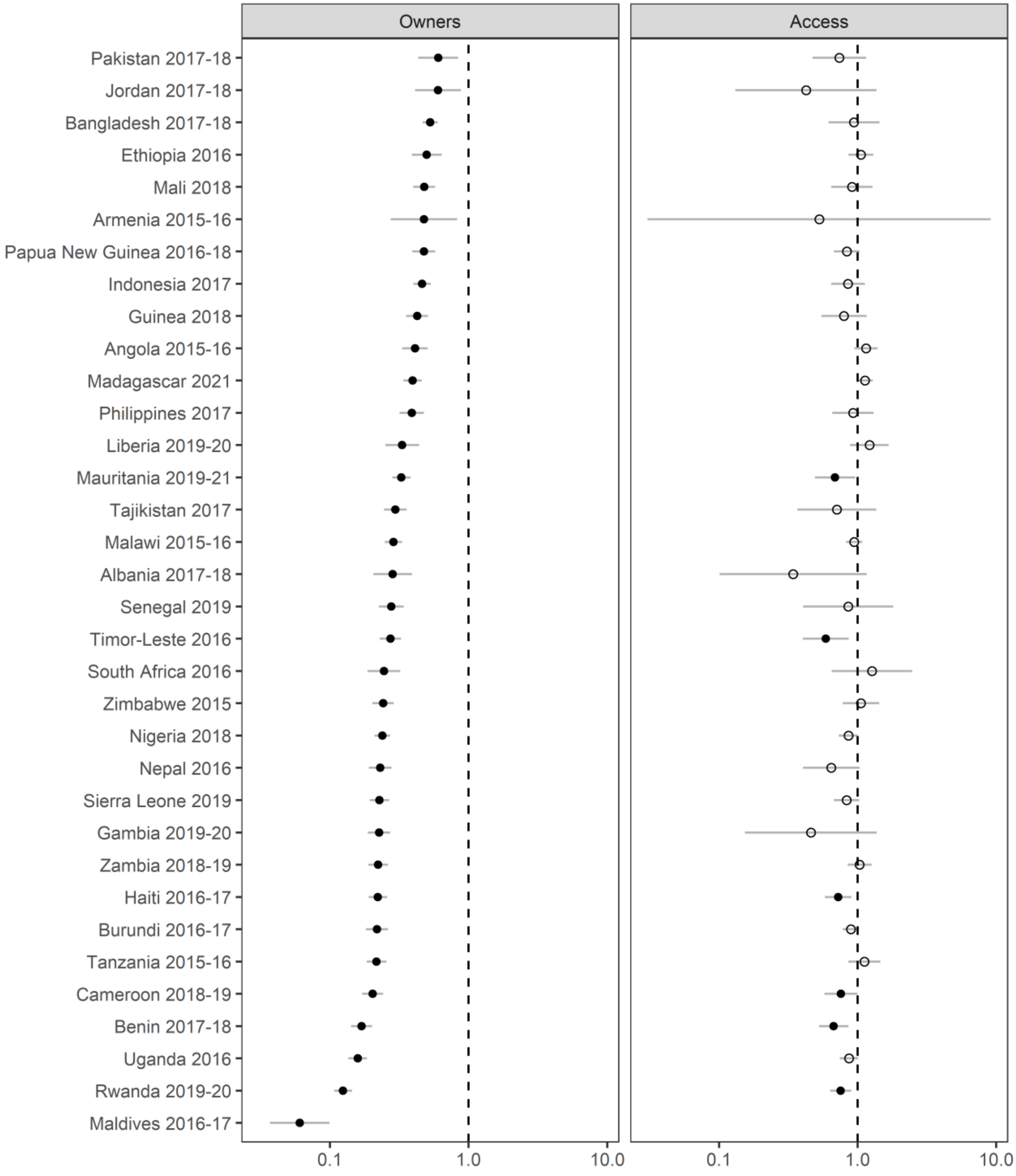

Note: Estimates for access to a mobile phone cannot be computed for Maldives due to small sample size. Fill dots mean that estimates are statistically significant at the 95% confidence level, while empty dots mean that estimates are not statistically significant.

Appendix Figure 1.2: Effect of the 25-29 age group on the odds of owning and having access to a mobile phone from binomial logistic regressions (20-24 age group is the reference). X-axis in log scale.

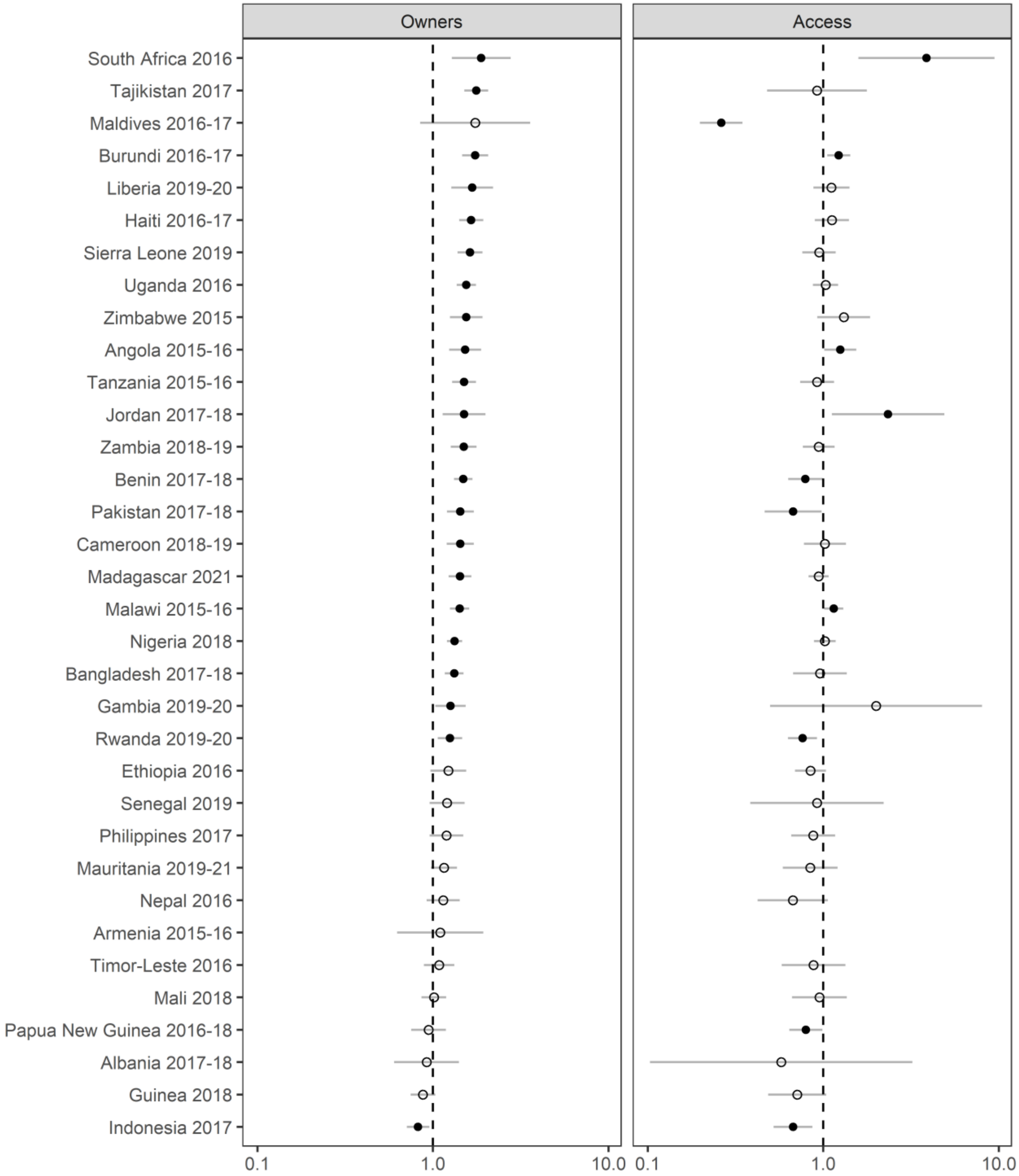

Note: Estimates for access to a mobile phone cannot be computed for Armenia due to small sample size. Fill dots mean that estimates are statistically significant at the 95% confidence level, while empty dots mean that estimates are not statistically significant.

Appendix Figure 1.3: Effect of the 30-34 age group on the odds of owning and having access to a mobile phone from binomial logistic regressions (20-24 age group is the reference). X-axis in log scale.

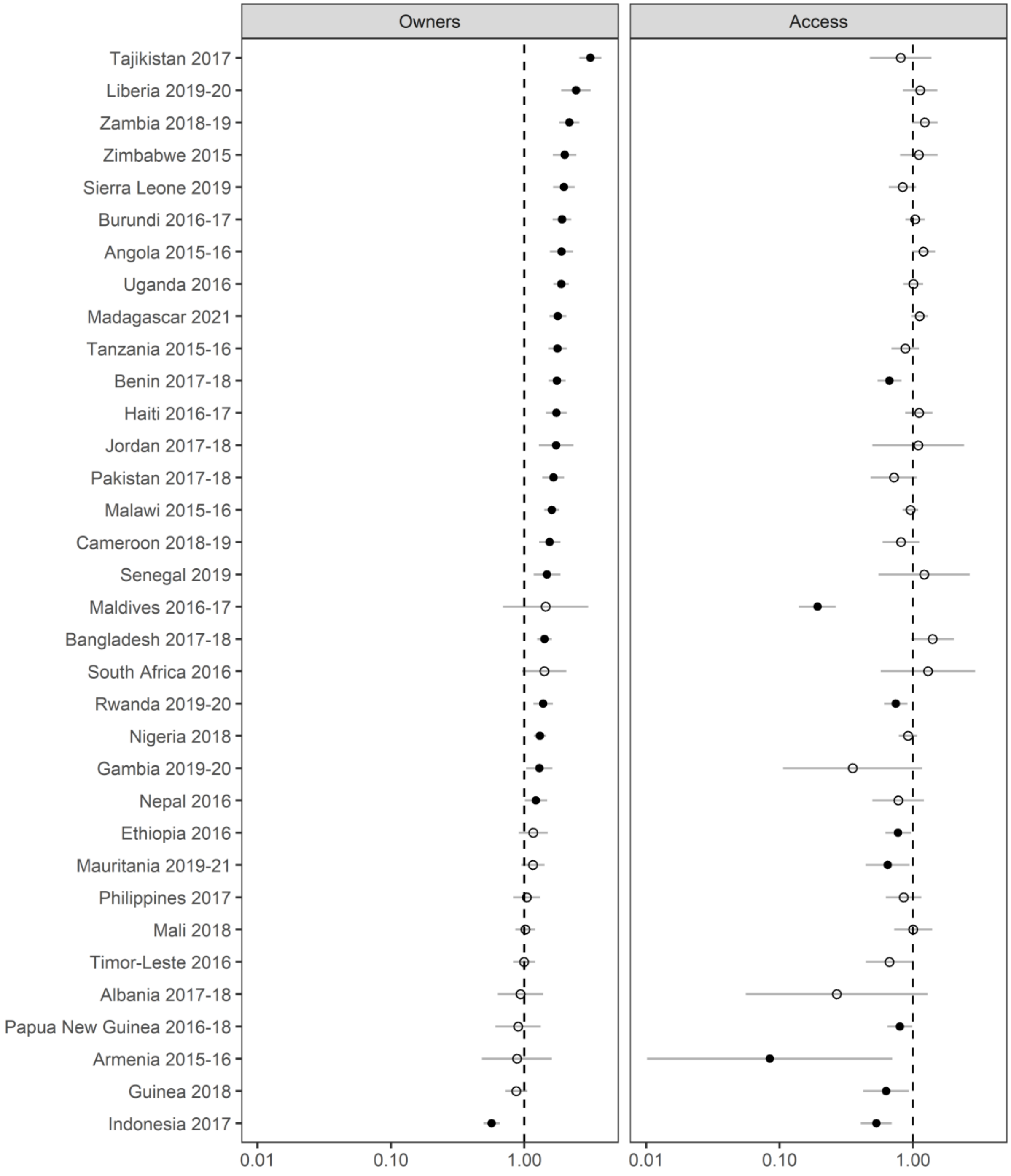

Note: Fill dots mean that estimates are statistically significant at the 95% confidence level, while empty dots mean that estimates are not statistically significant.

Appendix Figure 1.4: Effect of the 35-39 age group on the odds of owning and having access to a mobile phone from binomial logistic regressions (20-24 age group is the reference). X-axis in log scale.

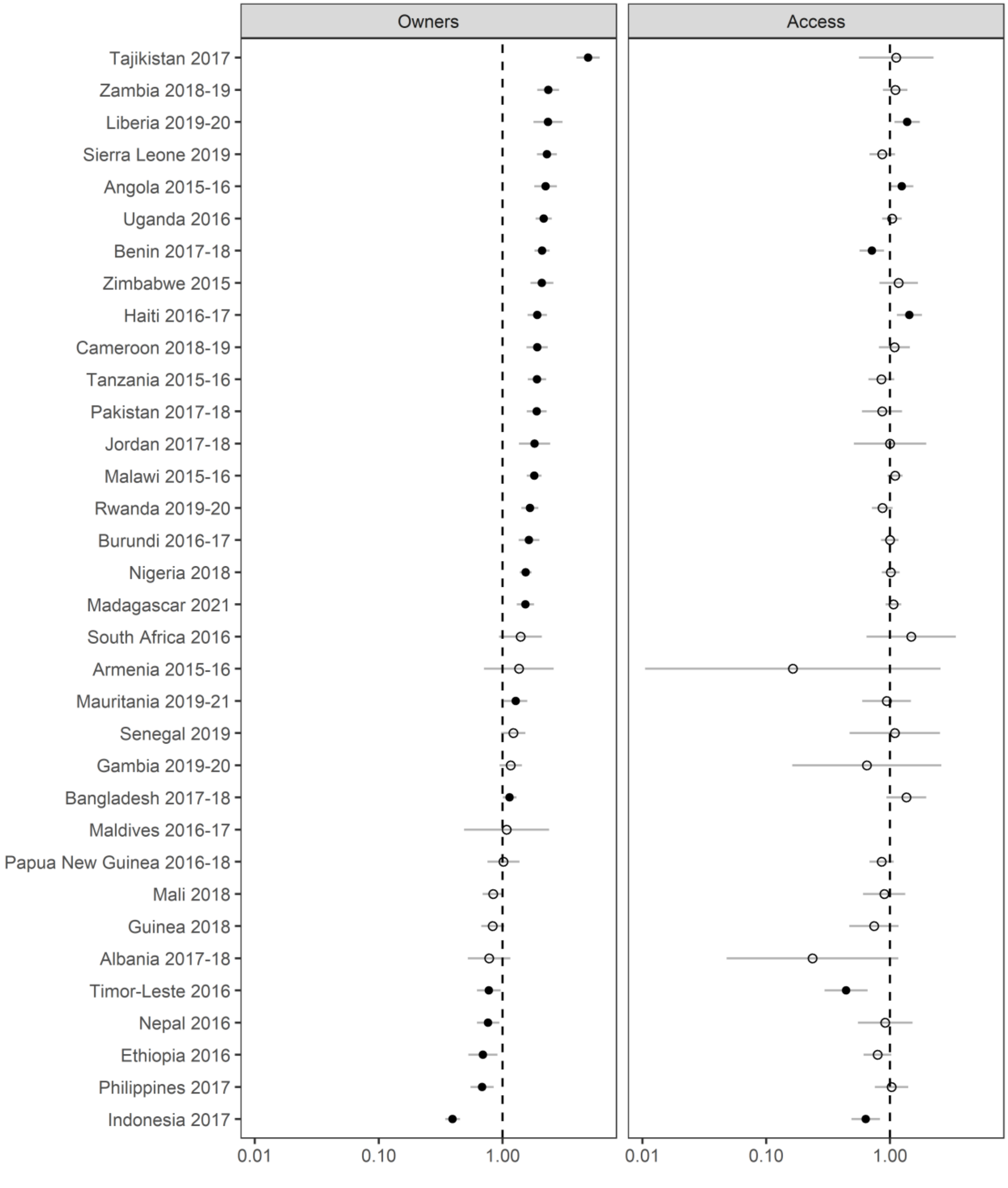

Note: Estimates for access to a mobile phone cannot be computed for Maldives due to small sample size. Fill dots mean that estimates are statistically significant at the 95% confidence level, while empty dots mean that estimates are not statistically significant.

Appendix Figure 1.5: Effect of the 40-44 age group on the odds of owning and having access to a mobile phone from binomial logistic regressions (20-24 age group is the reference). X-axis in log scale.

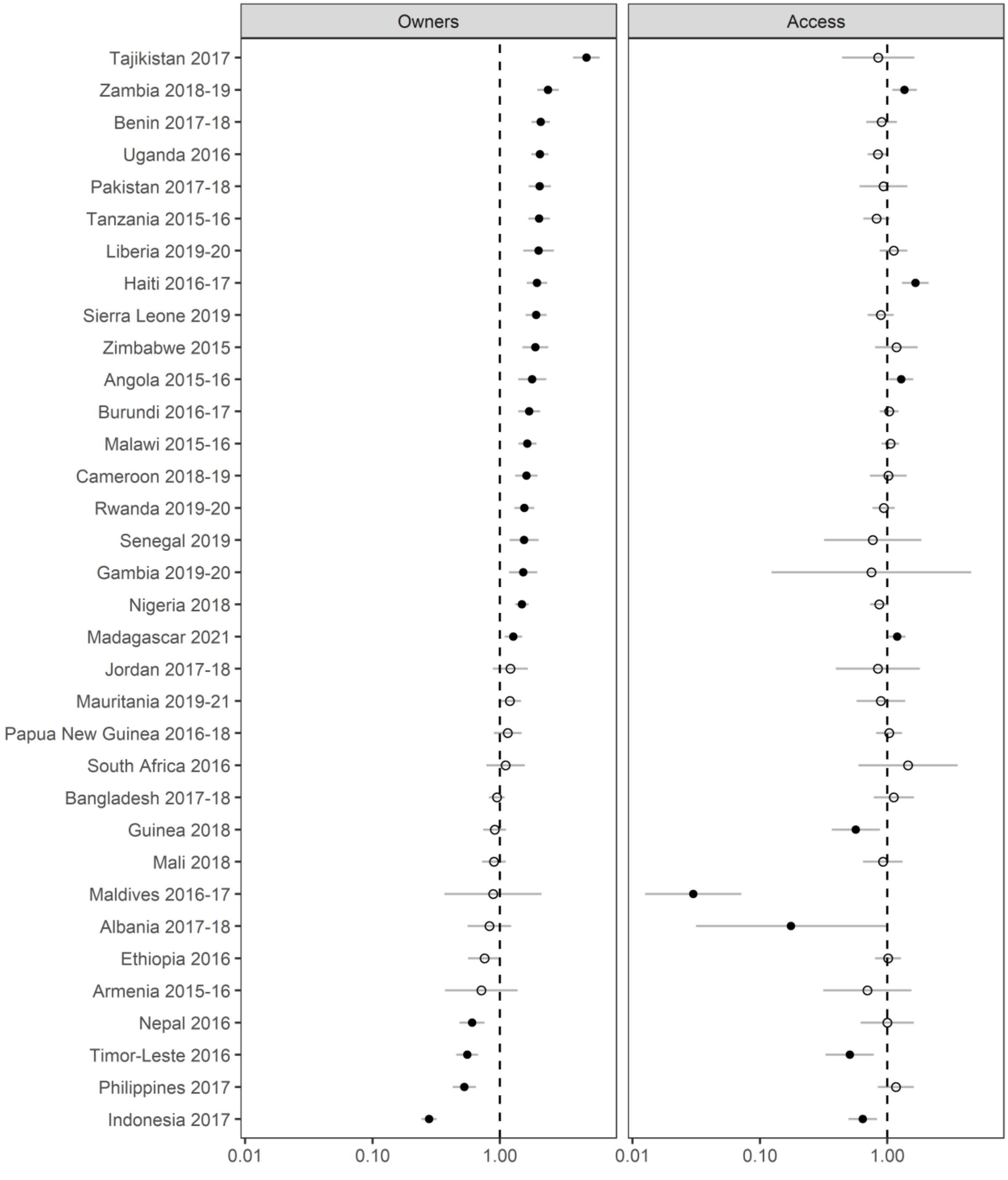

Note: Fill dots mean that estimates are statistically significant at the 95% confidence level, while empty dots mean that estimates are not statistically significant.

Appendix Figure 1.6: Effect of the 45-49 age group on the odds of owning and having access to a mobile phone from binomial logistic regressions (20-24 age group is the reference). X-axis in log scale.

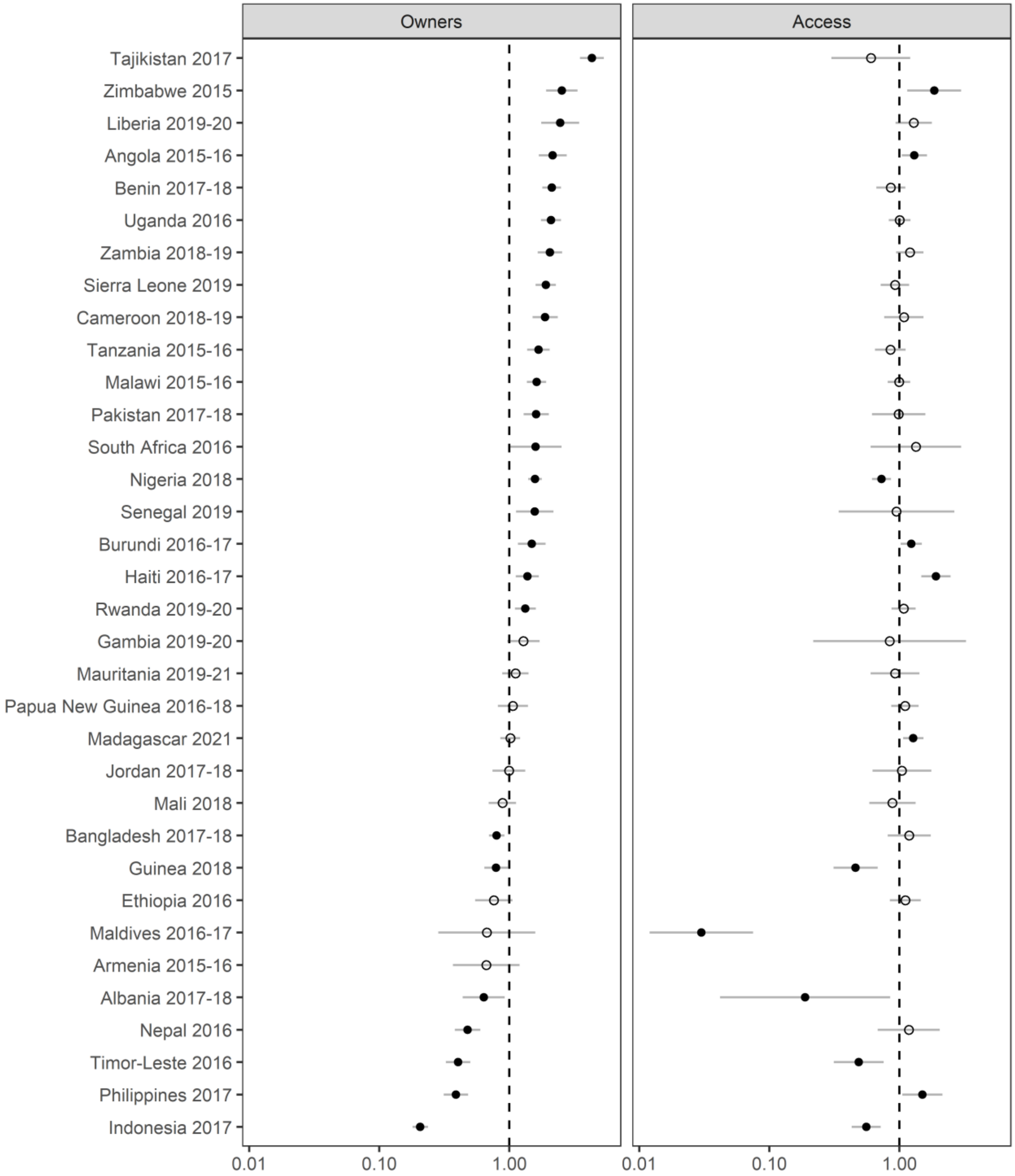

Note: Estimates for access to a mobile phone cannot be computed for Armenia due to small sample size. Fill dots mean that estimates are statistically significant at the 95% confidence level, while empty dots mean that estimates are not statistically significant.

Appendix Figure 2.1: Effect of primary education on the odds of owning and having access to a mobile phone from binomial logistic regressions (no education is the reference). X-axis in log scale.

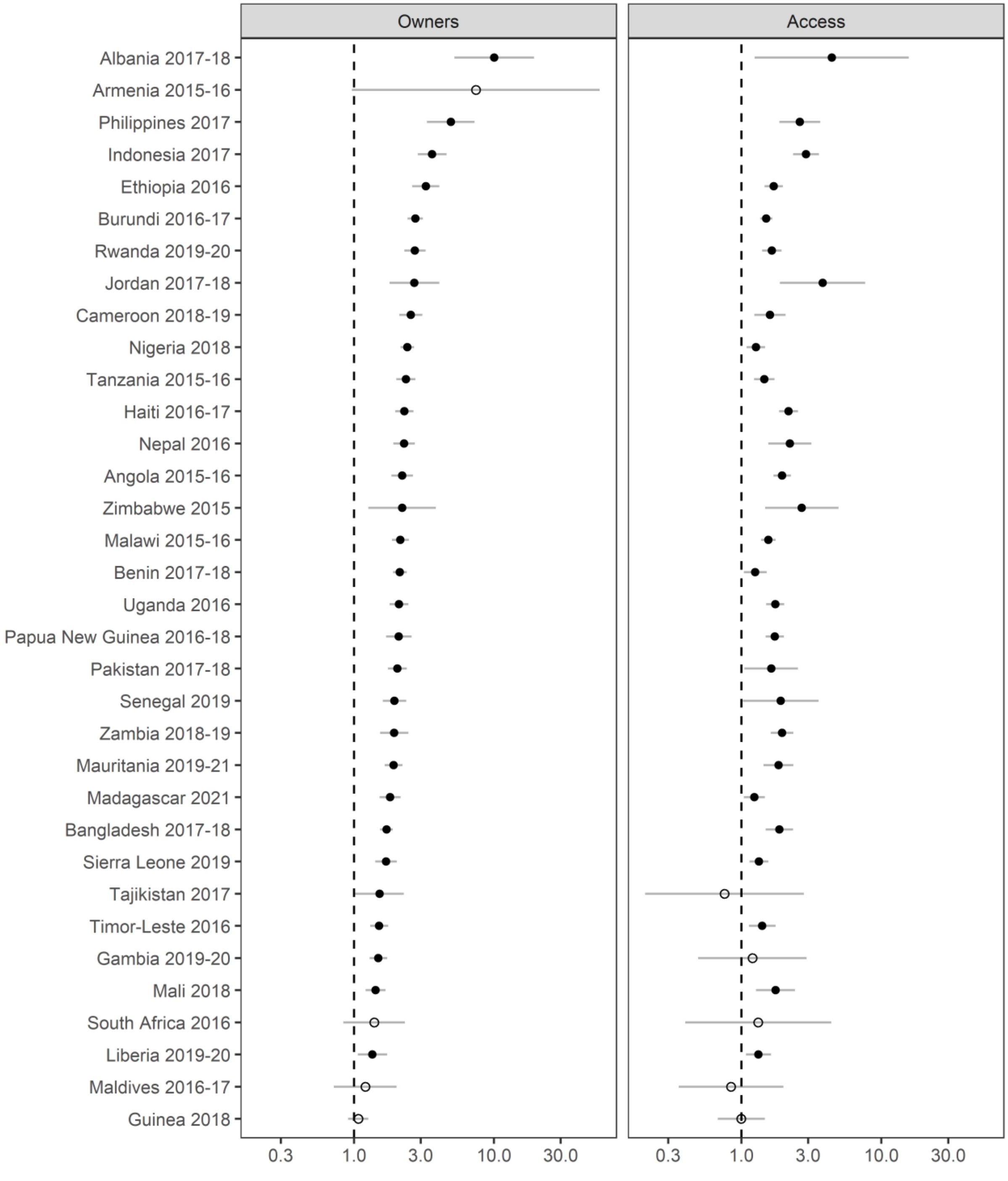

Note: Estimates for access to a mobile phone cannot be computed for Armenia due to small sample size. Fill dots mean that estimates are statistically significant at the 95% confidence level, while empty dots mean that estimates are not statistically significant.

Appendix Figure 2.2: Effect of secondary and higher education on the of odds owning and having access to a mobile phone from binomial logistic regressions (no education is the reference). X-axis in log scale.

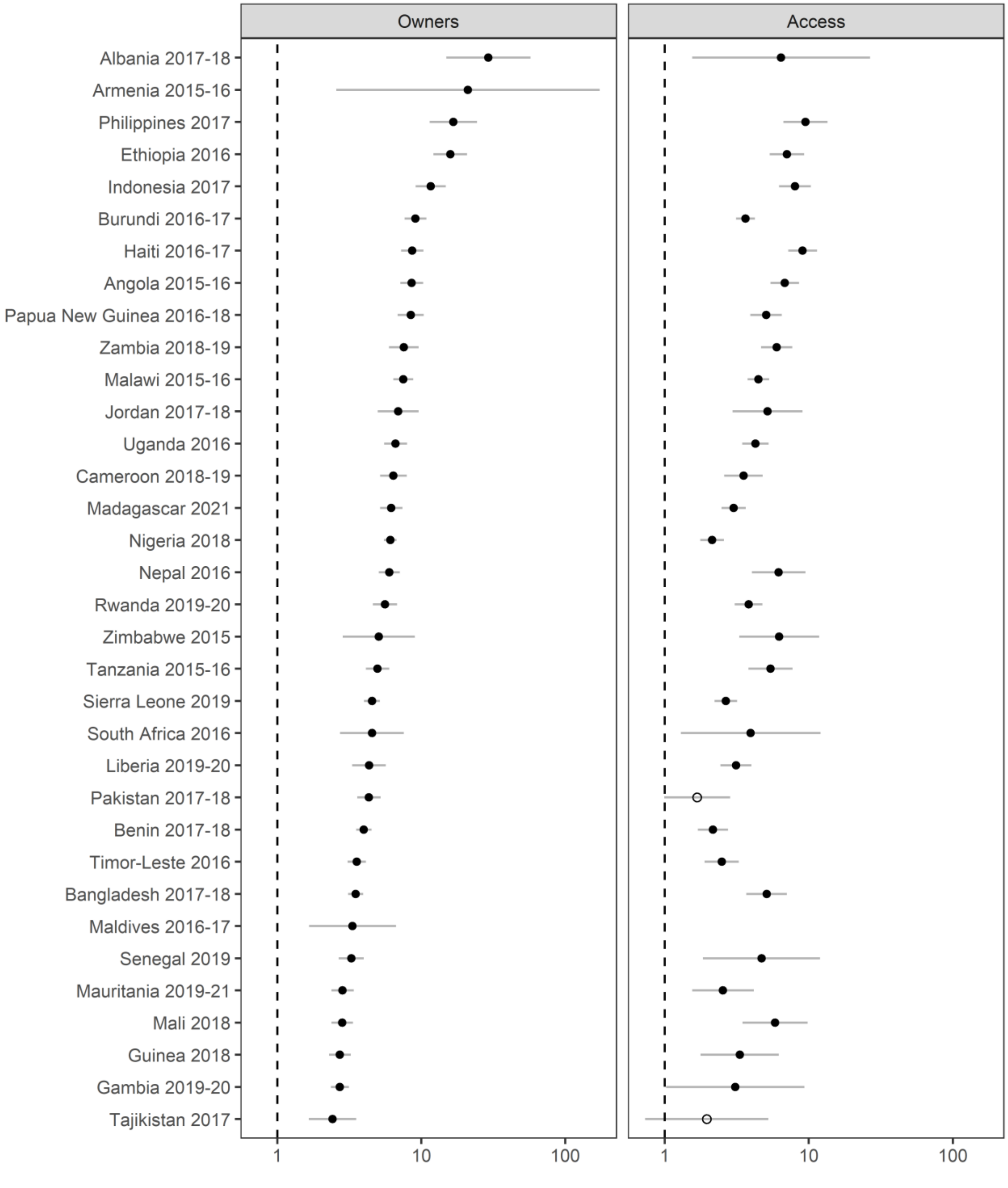

Note: Estimates for access to a mobile phone cannot be computed for Armenia and Maldives due to small sample size. Fill dots mean that estimates are statistically significant at the 95% confidence level, while empty dots mean that estimates are not statistically significant.

Appendix Figure 3: Effect of living in urban areas on the odds of owning and having access to a mobile phone from binomial logistic regressions (rural area is the reference). X-axis in log scale.

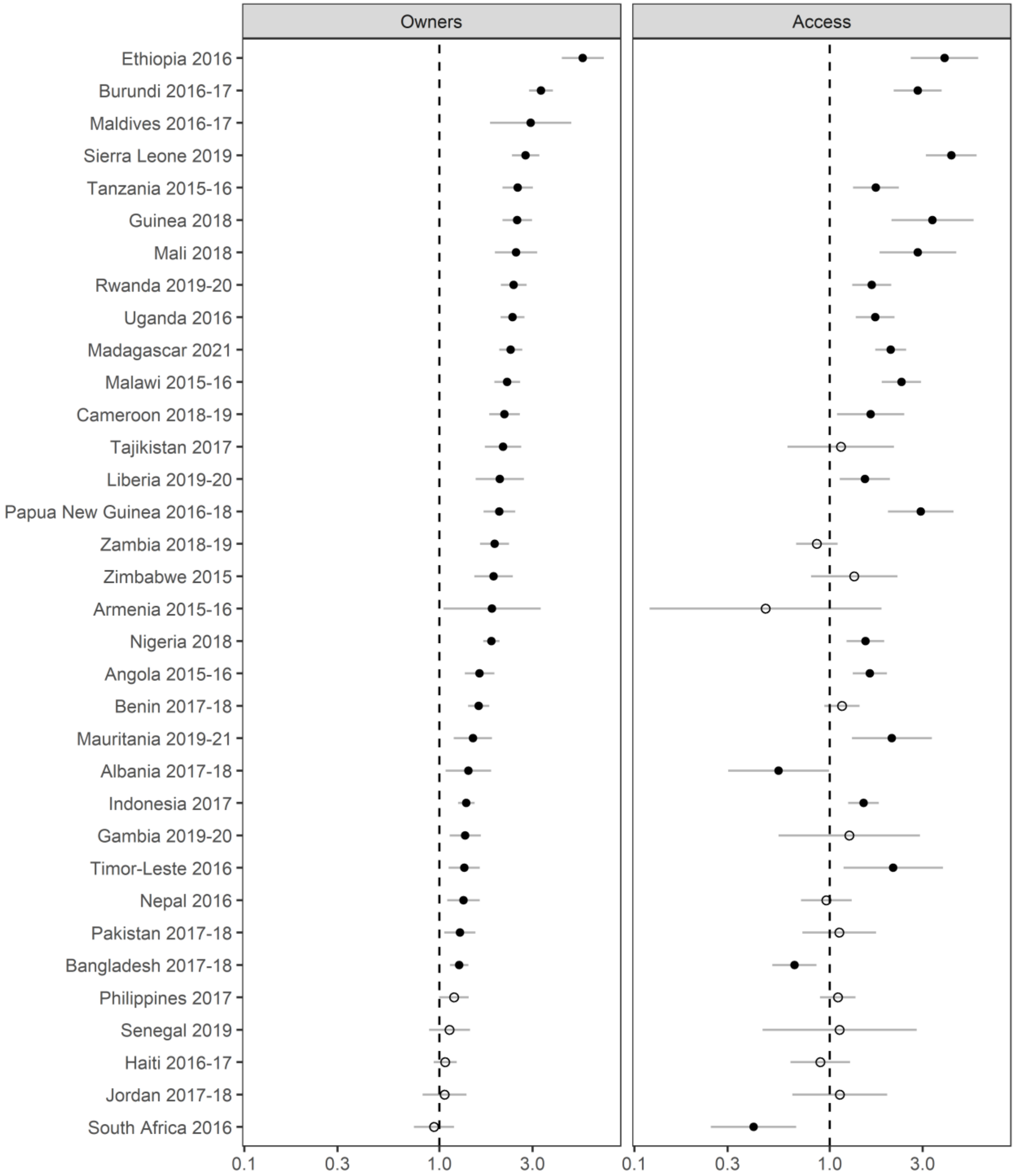

Note: Estimates for access to a mobile phone cannot be computed for Maldives due to small sample size. Fill dots mean that estimates are statistically significant at the 95% confidence level, while empty dots mean that estimates are not statistically significant.

Appendix Figure 4: Effect of being non-poor on the odds of owning and having access to a mobile phone from binomial logistic regressions (poor is the reference). X-axis in log scale.

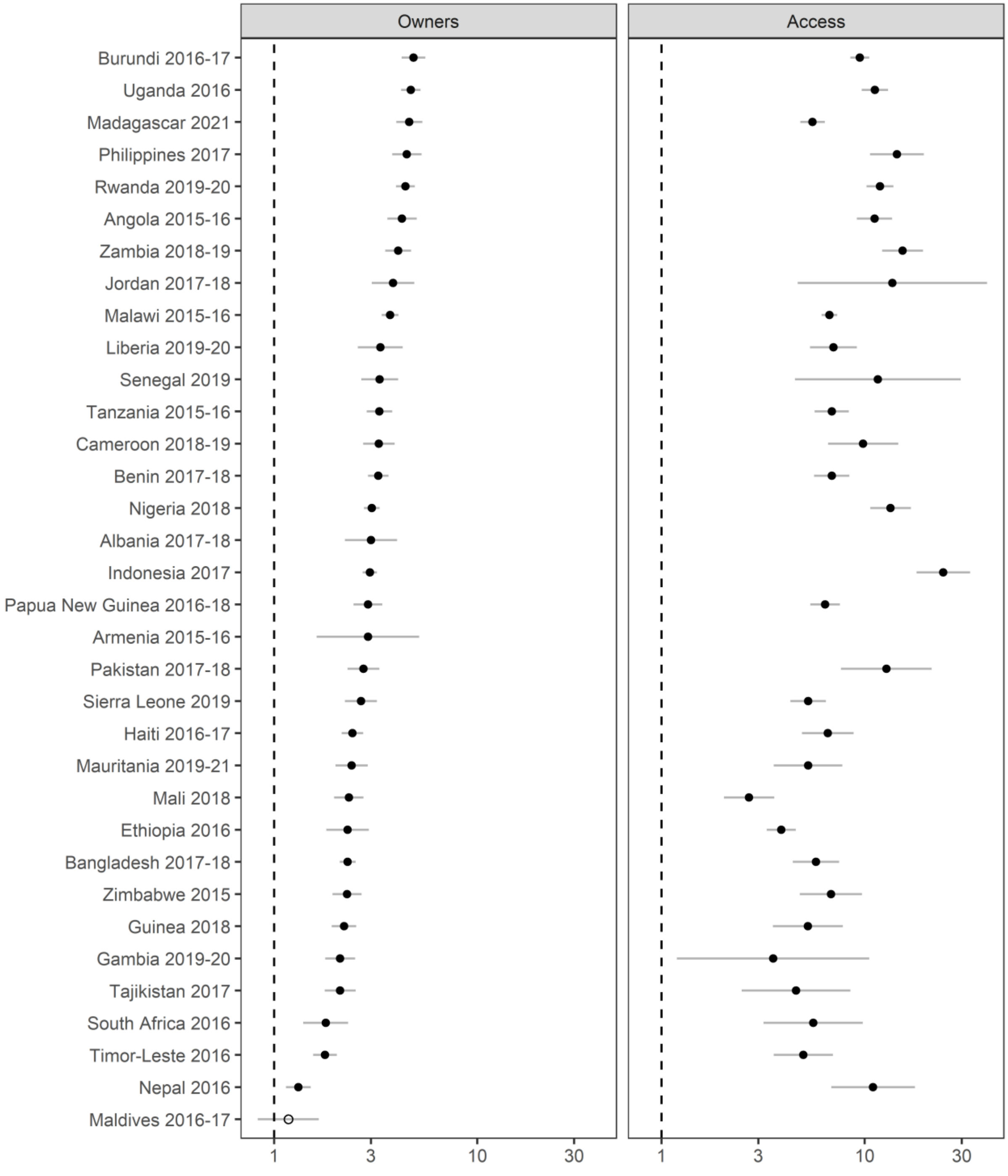

Note: Estimates for access to a mobile phone cannot be computed for Albania, Armenia, and Maldives due to small sample size. Fill dots mean that estimates are statistically significant at the 95% confidence level, while empty dots mean that estimates are not statistically significant.

Appendix Figure 5: Effect of not being in union on the odds of owning and having access to a mobile phone from binomial logistic regressions (married is the reference). X-axis in log scale.

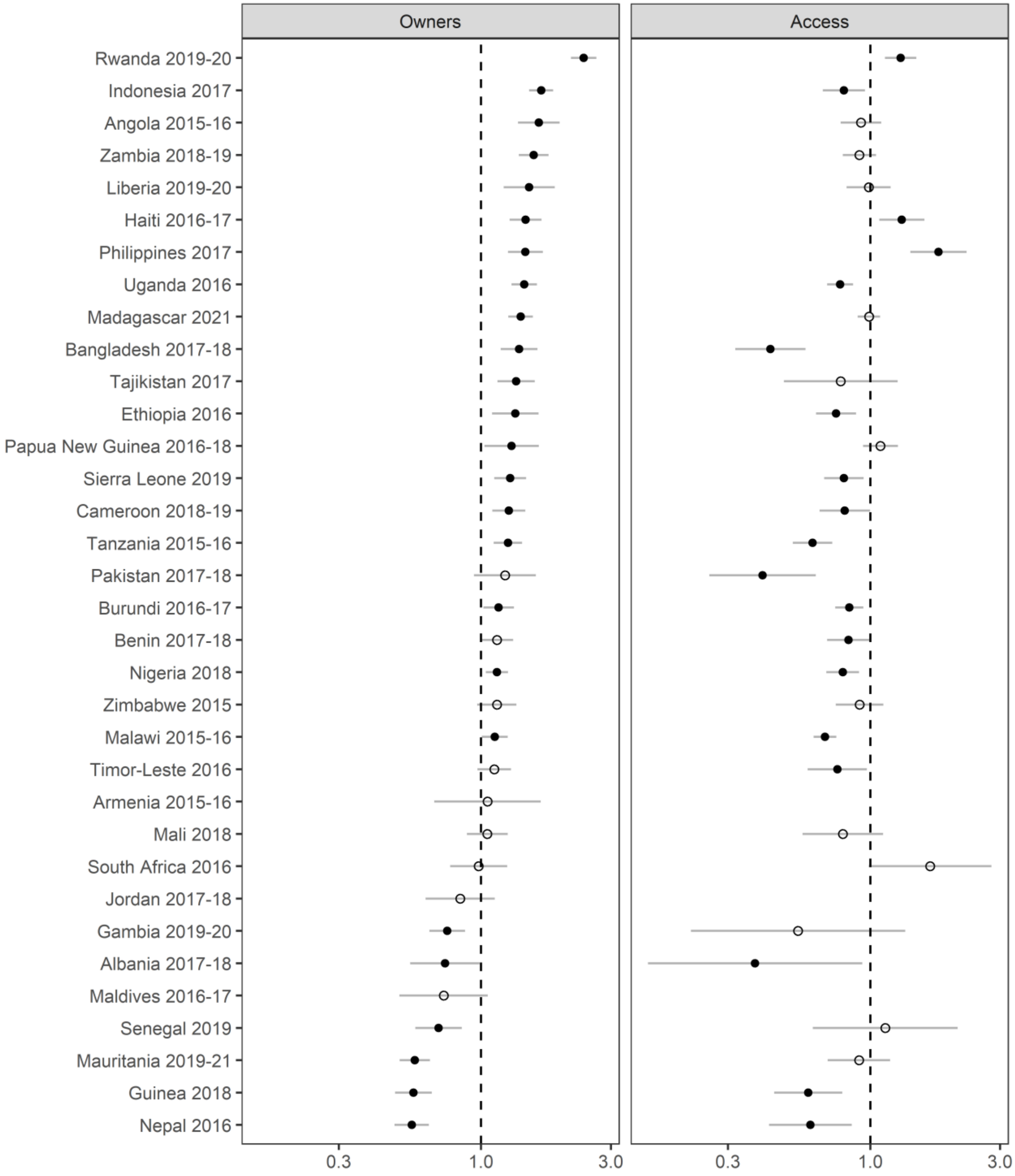

Note: Estimates for access to a mobile phone cannot be computed for Armenia, Jordan, and Maldives due to small sample size. Fill dots mean that estimates are statistically significant at the 95% confidence level, while empty dots mean that estimates are not statistically significant.

Appendix Figure 6: Under-five mortality rate and total fertility rate estimates testing for post-stratification weights from women who have access to a mobile phone.

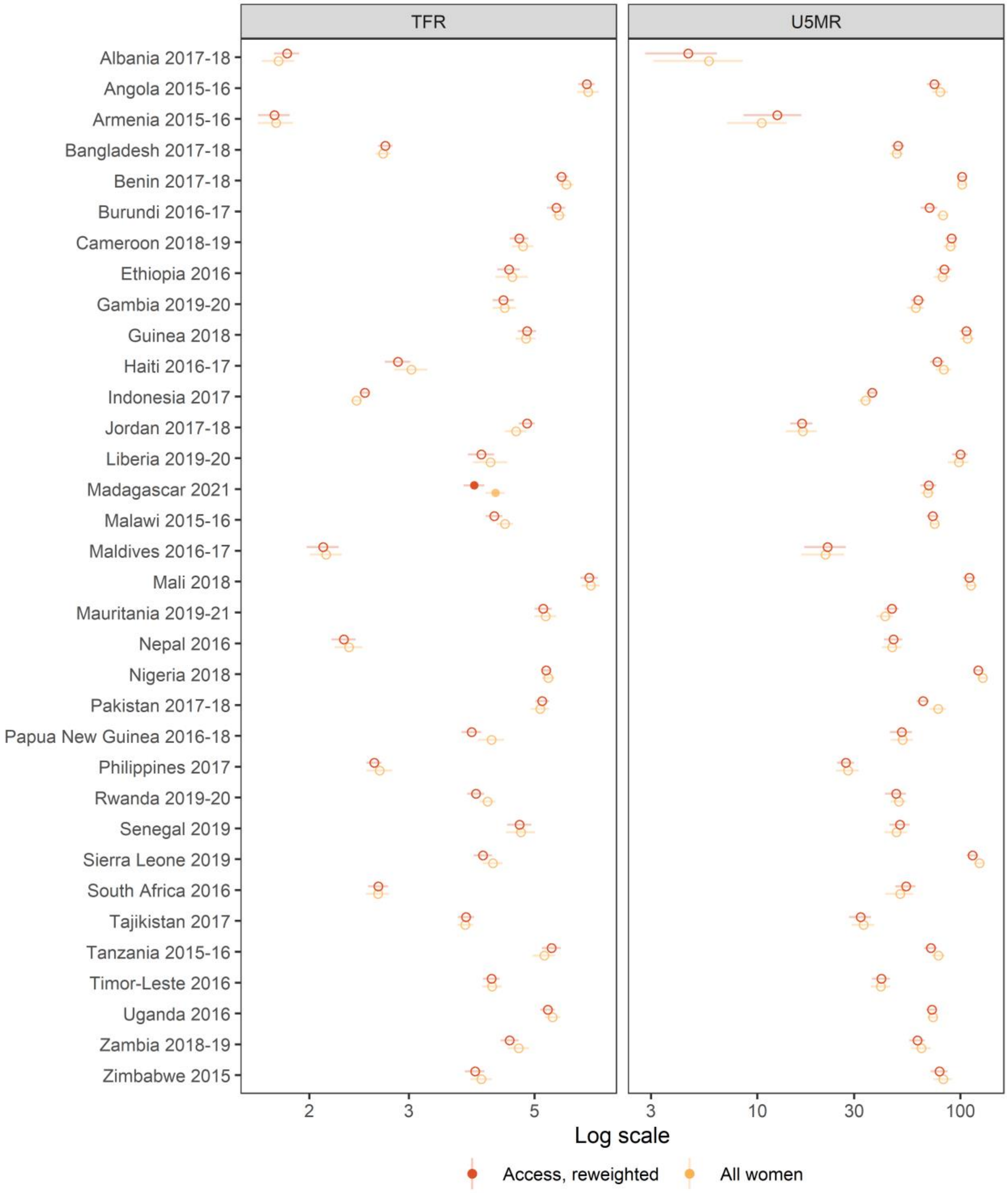

Note: Fill dots mean that the difference between the actual rate and the re-weighted rate is statistically significant at the 95% confidence level, while empty dots mean that the difference is not statistically significant.

Appendix Figure 7: Estimates of the difference in current use of modern contraceptives (MCU), sexual activity (SexAct), duration of postpartum amenorrhea (Amen), and duration of postpartum abstinence (Abst), between having and not having access to a mobile phone.

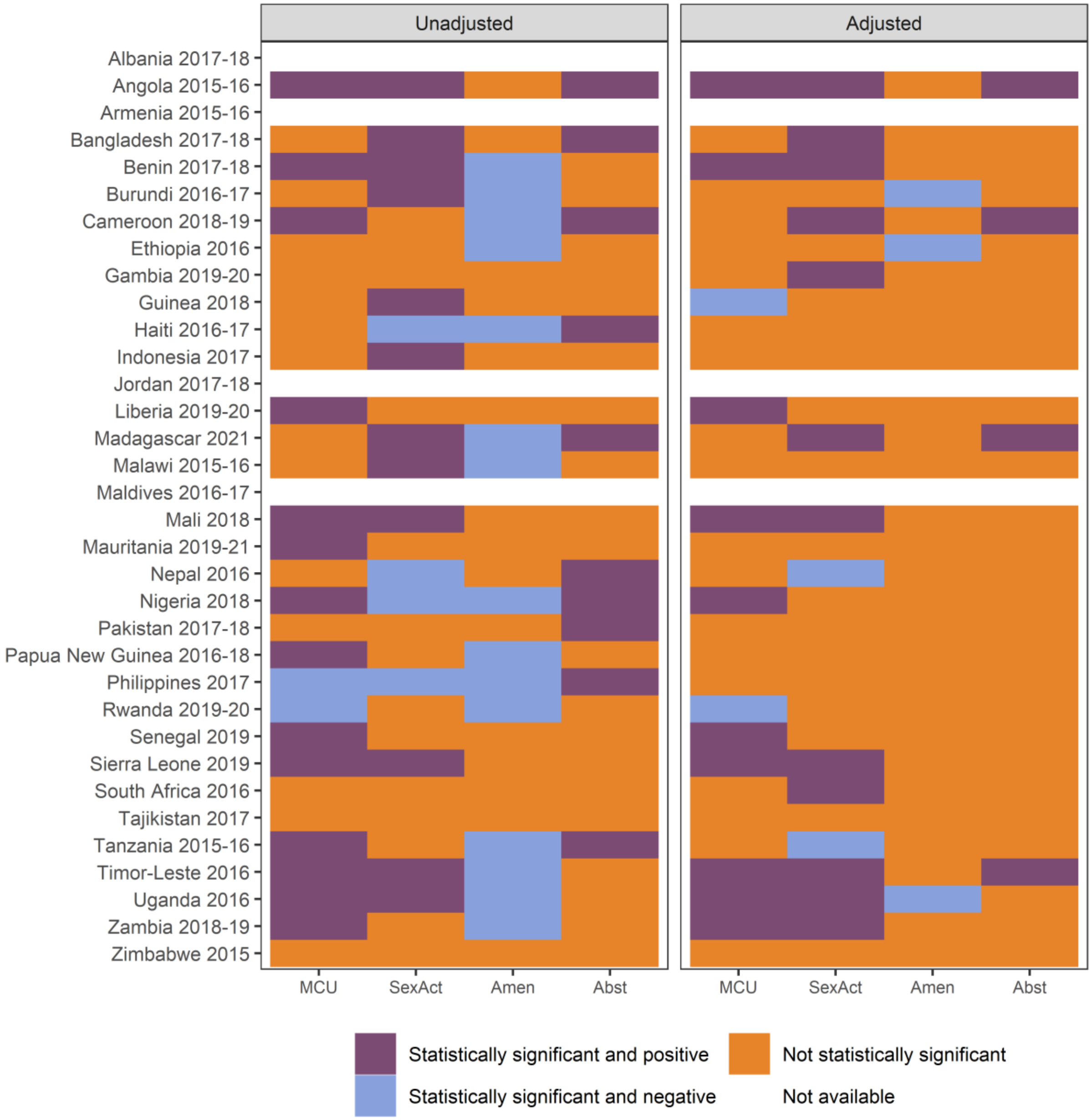

Appendix Figure 8: Estimates of the difference in exclusive breastfeeding (BrFeed), births who had at least 4 antenatal care visits (ANC), newborns who received at least 2 signal functions (PNC), children who received basic immunization (Vax), births delivered by skilled professionals (Skill), children who are underweight (UnderW), between having and not having access to a mobile phone.

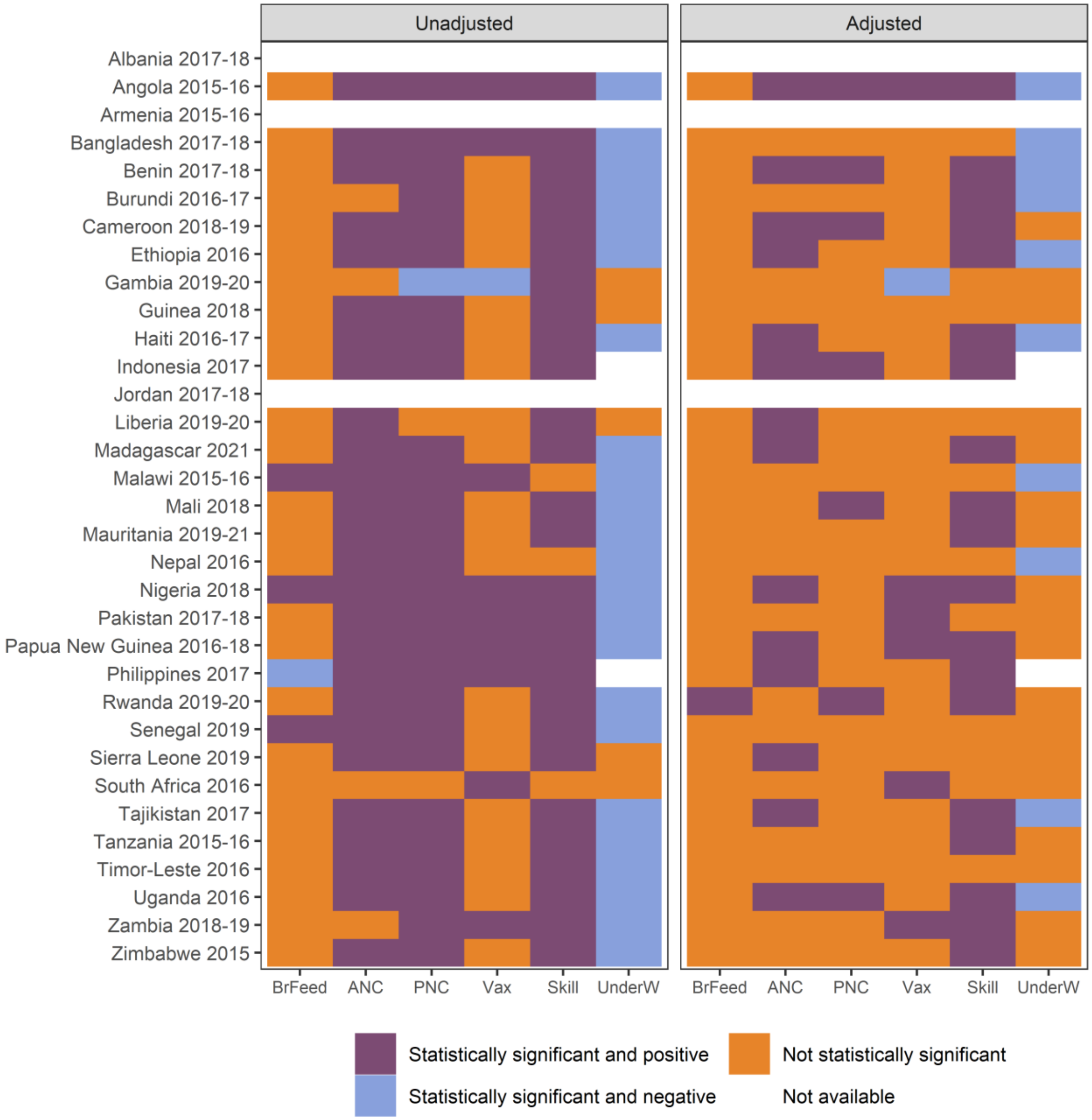

STROBE Statement—Checklist of items that should be included in reports of ***cross-sectional studies***

	Item No	Recommendation	Page No
**Title and abstract**	1	(*a*) Indicate the study's design with a commonly used term in the title or the abstract	2
		(*b*) Provide in the abstract an informative and balanced summary of what was done and what was found	2
**Introduction**			
Background/rationale	2	Explain the scientific background and rationale for the investigation being reported	4-6
Objectives	3	State specific objectives, including any prespecified hypotheses	6
**Methods**			
Study design	4	Present key elements of study design early in the paper	6-8
Setting	5	Describe the setting, locations, and relevant dates, including periods of recruitment, exposure, follow-up, and data collection	7
Participants	6	(*a*) Give the eligibility criteria, and the sources and methods of selection of participants	NA
Variables	7	Clearly define all outcomes, exposures, predictors, potential confounders, and effect modifiers. Give diagnostic criteria, if applicable	7-9
Data sources/ measurement	8*	For each variable of interest, give sources of data and details of methods of assessment (measurement). Describe comparability of assessment methods if there is more than one group	6-7
Bias	9	Describe any efforts to address potential sources of bias	7-9
Study size	10	Explain how the study size was arrived at	6-7
Quantitative variables	11	Explain how quantitative variables were handled in the analyses. If applicable, describe which groupings were chosen and why	7-9
Statistical methods	12	(*a*) Describe all statistical methods, including those used to control for confounding	7-9
		(*b*) Describe any methods used to examine subgroups and interactions	8-9
		(*c*) Explain how missing data were addressed	7
		(*d*) If applicable, describe analytical methods taking account of sampling strategy	NA
		(*e*) Describe any sensitivity analyses	NA
**Results**			
Participants	13*	(a) Report numbers of individuals at each stage of study—eg numbers potentially eligible, examined for eligibility, confirmed eligible, included in the study, completing follow-up, and analysed	NA
		(b) Give reasons for non-participation at each stage	NA
		(c) Consider use of a flow diagram	NA
Descriptive data	14*	(a) Give characteristics of study participants (eg demographic, clinical, social) and information on exposures and potential confounders	21-22
		(b) Indicate number of participants with missing data for each variable of interest	7
Outcome data	15*	Report numbers of outcome events or summary measures	9-10
Main results	16	(*a*) Give unadjusted estimates and, if applicable, confounder-adjusted estimates and their precision (eg, 95% confidence interval). Make clear which confounders were adjusted for and why they were included	10-11

For peer review only - http://bmjopen.bmj.com/site/about/guidelines.xhtml

		(*b*) Report category boundaries when continuous variables were categorized	NA
		(*c*) If relevant, consider translating estimates of relative risk into absolute risk for a meaningful time period	NA
Other analyses	17	Report other analyses done—eg analyses of subgroups and interactions, and sensitivity analyses	11-13
**Discussion**			
Key results	18	Summarise key results with reference to study objectives	13-16
Limitations	19	Discuss limitations of the study, taking into account sources of potential bias or imprecision. Discuss both direction and magnitude of any potential bias	15-16
Interpretation	20	Give a cautious overall interpretation of results considering objectives, limitations, multiplicity of analyses, results from similar studies, and other relevant evidence	16
Generalisability	21	Discuss the generalisability (external validity) of the study results	16
**Other information**			
Funding	22	Give the source of funding and the role of the funders for the present study and, if applicable, for the original study on which the present article is based	16

*Give information separately for exposed and unexposed groups.

Note: An Explanation and Elaboration article discusses each checklist item and gives methodological background and published examples of transparent reporting. The STROBE checklist is best used in conjunction with this article (freely available on the Web sites of PLoS Medicine at http://www.plosmedicine.org/, Annals of Internal Medicine at http://www.annals.org/, and Epidemiology at http://www.epidem.com/). Information on the STROBE Initiative is available at www.strobe-statement.org.

For peer review only

For peer review only - http://bmjopen.bmj.com/site/about/guidelines.xhtml

BMJ Open

Measuring under-five mortality and fertility through mobile phone surveys: an assessment of selection bias in 34 low- and middle-income countries

Journal:	*BMJ Open*
Manuscript ID	bmjopen-2023-071791.R2
Article Type:	Original research
Date Submitted by the Author:	19-Oct-2023
Complete List of Authors:	Sánchez-Páez, David; Universidad de Valladolid, Department of Economics; Université catholique de Louvain, Centre for Demographic Research<br>Masquelier, Bruno; Université catholique de Louvain, Centre for Demographic Research<br>Menashe-Oren, Ashira; Université catholique de Louvain, Centre for Demographic Research<br>Baruwa, Ololade; University of the Witwatersrand Johannesburg<br>Reniers, Georges; London School of Hygiene and Tropical Medicine, Population Studies Group
**Primary Subject Heading**:	Research methods
Secondary Subject Heading:	Global health, Research methods
Keywords:	Demography < TROPICAL MEDICINE, STATISTICS & RESEARCH METHODS, Epidemiology < TROPICAL MEDICINE

SCHOLARONE™
Manuscripts

I, the Submitting Author has the right to grant and does grant on behalf of all authors of the Work (as defined in the below author licence), an exclusive licence and/or a non-exclusive licence for contributions from authors who are: i) UK Crown employees; ii) where BMJ has agreed a CC-BY licence shall apply, and/or iii) in accordance with the terms applicable for US Federal Government officers or employees acting as part of their official duties; on a worldwide, perpetual, irrevocable, royalty-free basis to BMJ Publishing Group Ltd ("BMJ") its licensees and where the relevant Journal is co-owned by BMJ to the co-owners of the Journal, to publish the Work in this journal and any other BMJ products and to exploit all rights, as set out in our licence.

The Submitting Author accepts and understands that any supply made under these terms is made by BMJ to the Submitting Author unless you are acting as an employee on behalf of your employer or a postgraduate student of an affiliated institution which is paying any applicable article publishing charge ("APC") for Open Access articles. Where the Submitting Author wishes to make the Work available on an Open Access basis (and intends to pay the relevant APC), the terms of reuse of such Open Access shall be governed by a Creative Commons licence – details of these licences and which Creative Commons licence will apply to this Work are set out in our licence referred to above.

Other than as permitted in any relevant BMJ Author's Self Archiving Policies, I confirm this Work has not been accepted for publication elsewhere, is not being considered for publication elsewhere and does not duplicate material already published. I confirm all authors consent to publication of this Work and authorise the granting of this licence.

For peer review only

Measuring under-five mortality and fertility through mobile phone surveys: an assessment of selection bias in 34 low- and middle-income countries

AUTHORS:

1. David A. Sánchez-Páez (Corresponding author).
   Affiliation: Department of Economics, Universidad de Valladolid.
   Address: Avenida del Valle Esgueva 6
   47011 Valladolid
   Spain.
   E-mail: david.sanchezpaez@uva.es
   ORCID: 0000-0002-7828-8193

2. Bruno Masquelier.
   Affiliation: Centre for Demographic Research, Université catholique de Louvain.
   Address: Louvain-la-Neuve, Belgium.
   ORCID: 0000-0002-9585-891X

3. Ashira Menashe-Oren.
   Affiliation: Centre for Demographic Research, Université catholique de Louvain.
   Address: Louvain-la-Neuve, Belgium.
   ORCID: 0000-0002-4781-5384

4. Ololade Julius Baruwa.
   Affiliation: Centre for Demographic Research, Université catholique de Louvain.
   Address: Louvain-la-Neuve, Belgium.
   ORCID: 0000-0002-0776-528X

5. Georges Reniers.
   Affiliation: Population Studies Group, London School of Hygiene & Tropical Medicine.
   Address: London, United Kingdom.
   ORCID: 0000-0001-6582-1692

Keywords: Under-five mortality; fertility; mobile phone surveys; low- and middle-income countries; selection bias; Demographic and Health Surveys; mobile phone ownership; mobile phone access.

Word count: 3,929 exclusive of title page, abstract, summary box, references, figures, tables, and declaration statements.

For peer review only

ABSTRACT

Objectives: To assess sample selection bias in mobile phone survey estimates of fertility and under-five mortality.

Design: With data from the Demographic and Health Surveys, we use logistic regressions to identify socio-demographic correlates of mobile phone ownership and access, and Poisson regressions to estimate the association between mobile phone ownership (or access) and total fertility and under-five mortality rates. We evaluate the potential reasons why estimates by mobile phone ownership may differ using a set of behavioral characteristics.

Setting: 34 low- and middle-income countries, mostly in sub-Saharan Africa.

Participants: 534,536 women between the ages of 15 and 49.

Outcome measures: Under-five mortality rate (U5MR) and total fertility rate (TFR).

Results: Mobile phone ownership ranges from 23.6% in Burundi to 96.7% in Armenia. The median TFR ratio and U5MR ratio between the non-owners and the owners of a mobile phone are 1.48 and 1.29, respectively. Fertility and mortality rates would be biased downwards if estimates are only based on women who own or have access to mobile phones. Estimates of U5MR can be adjusted through post-stratification using age, educational level, area of residence, wealth, and marital status as weights. However, estimates of TFR remain biased even after adjusting for these covariates. This difference is associated with behavioral factors (e.g., contraceptive use) that are not captured by the post-stratification variables, but for which there are also differences between mobile phone owners and non-owners.

For peer review only

Conclusions: Mobile phone surveys need to collect data on socio-demographic background characteristics to be able to weight and adjust mortality estimates ex post-facto. Fertility estimates from mobile phone surveys will be biased unless further research uncovers the mechanisms driving the bias.

Strengths and limitations of this study

- Mobile phone ownership and access are associated with socio-demographic background attributes, which could bias demographic estimates derived from data collected through mobile phone surveys.
- We established that fertility and under-five mortality estimates derived from birth histories administered for women who own or have access to a mobile phone will be biased downwards.
- Post-stratification on socio-demographic background characteristics corrects the bias in under-five mortality estimates, but adjusted fertility estimates remain lower than in a nationally representative sample including women who do not own or have access to a mobile phone.
- This study evaluates selection bias associated with the limitation of the sampling frame in a mobile phone survey, but does not evaluate bias that may result from selective participation in a mobile phone survey.
- Mobile phone ownership is increasing rapidly in many low- and middle-income countries, but its distribution will remain uneven for some time, so sample selection bias should be considered when deriving demographic estimates from mobile phone surveys.

For peer review only

INTRODUCTION

Use of mobile phones has spread widely across the world, including in low- and middle-income countries (LMIC) where the technological infrastructure for landlines is often lacking [1,2]. This spread has allowed populations to communicate faster and effortlessly, to access internet, and it has accelerated social and economic development [3,4]. Widespread and increasing coverage of mobile phones also provides an opportunity to conduct mobile phone surveys (MPS). Some benefits of MPS include cost efficiency, shorter fieldwork duration due to reduced time devoted to travel, improved ability to supervise data collection through the recording of interviews and automated storage of paradata, and increased access to populations that are harder to reach [5,6]. MPS have already covered some health-related topics, including family planning and sexual and reproductive health [1,7], but few MPS have included questions to monitor trends in fertility and mortality.

In countries lacking comprehensive systems of civil registration and vital statistics, data on mortality and fertility are generally collected through face-to-face interviews in survey programs such as the Demographic and Health Surveys (DHS) or Multiple Indicator Cluster Surveys (MICS). However, face-to-face interviews might not be the most cost-effective approach and they are not suitable in settings affected by conflicts, disasters or epidemic outbreaks. During the Ebola epidemic in Liberia, MPS were used to estimate mortality both from Ebola and other causes based on reports of recent household deaths [8]. During the COVID-19 pandemic, MPS were proposed as a viable way to assess excess mortality without posing additional risks to survey teams and respondents [9,10]. MPS thus represent a promising alternative to collect demographic data.

For peer review only

MPS have several disadvantages that require careful consideration, such as higher non-response rates and shortened interview duration – requiring changes to the questionnaires used in face-to-face interviews [11]. Moreover, MPS may not be representative, especially when based on random digit dialing, as compared to a survey using phone numbers previously collected in face-to-face surveys [1]. This is because inequalities in use of digital technology, known as a "digital divide", persist. Mobile phone use is related to gender, socioeconomic status, technological proficiency and literacy. Women have been noted to be at a disadvantage in mobile phone use, although the more common mobile phones are, the smaller the gender gap is [12–14]. This gender divide in mobile phone use reflects multiple barriers women face, including lack of financial resources, lack of time due to care responsibilities or household chores, socio-cultural restrictions, and lower educational attainment [15]. Mobile phone users are typically also better educated [16] and younger [17]. Moreover, rural dwellers use mobile phones relatively less than urbanites [18], although overall access remains high in rural areas as well [19]. Other factors, not related directly to an individual's characteristics, also determine selectivity in mobile phone users. For example, in remote locations and near borders, cellular network coverage may be weak, leaving populations living in such areas under-represented in MPS.

At the aggregate level, use of mobile phone has been linked to sustainable development [2]. The higher the diffusion of mobile phones, the lower under-five (U5M) and maternal mortality, the greater the use of contraception, and the smaller ideal family size [2,20]. This is in part because mobile phone use is a good proxy for socioeconomic development, but also because mobile phone use can improve access to health services, including reproductive health and antenatal care [21–25]. Such benefits could hinder obtaining a representative sample, even after post-sampling weighting. For example, a study conducted in Burkina Faso found higher modern contraception

For peer review only

use among respondents reached by an MPS (40%) compared to respondents in a face-to-face survey (26%) [7]. This difference in modern contraceptive use persisted after post-stratification. Similarly, Lefevre and colleagues [26] explored variations in several reproductive maternal, newborn and child health indicators, such as antenatal and postnatal care, skilled attendance at birth, measles and tetanus vaccinations, using DHS surveys in 15 countries. Even after controlling for a wide range of sociodemographic variables, child health and natal care was better when mothers had a mobile phone. A notable exception was the adoption of initial, exclusive, and continuous breastfeeding, which was less common among mobile phone users.

These previous studies suggest that fertility and mortality estimates derived from MPS are likely to be biased, but this issue has not been systematically evaluated. In this study, we leverage existing DHS data containing information on mobile phone use by women to assess the selection bias in U5M and fertility estimates. We expect that these estimates are biased downwards, since mortality and fertility tend to be higher in lower socioeconomic groups [27,28]. We assess mobile phone use by distinguishing between women owning a mobile phone (henceforth referred to as 'ownership') and those who can potentially be reached through another household member who owns a phone (henceforth referred to as 'access'). We first identify correlates of mobile phone ownership and access through logistic regressions and establish through Poisson regressions whether ownership and access are associated with variations in U5M and fertility.

DATA AND METHODS

Data collection

We used all available DHS [29] that include information on mobile phone ownership and access. The question on mobile phone *ownership* (v169a) is included in the individual questionnaire, while

For peer review only

the question on *access* to a mobile phone (hv243a) is in the household questionnaire. Access to mobile phone means that the woman either owns a mobile phone or lives in a household where the household head owns a mobile phone. Some countries have more than one survey with such data, so in these cases, we used the most recent one. In total, we used 34 DHS, mostly from Sub-Saharan Africa (see Appendix Table 1 in the supplementary material). These surveys were collected between 2015 and 2021. All women have complete information for all variables of interest included in this study.

The survey India 2019-21 is excluded from the analysis as the information is missing for about 85% of respondents. In Papua New Guinea 2016-18 information is missing for 0.4% of respondents, in Indonesia 2017 for 0.1%, and in Pakistan 2017-18 for 0.01%. We removed the observations with missing data from these three samples.

Data analysis

Using data from the birth histories administered for women aged 15–49, we computed total fertility rates (TFR) for the 36-month period preceding the survey and under-five mortality rates (U5MR) for the 10-year period preceding the survey. We used Poisson regressions to estimate the association between mobile phone ownership and access and fertility and U5M rates. For fertility, the outcome variable was the number of births, while for U5M, it was the number of deaths before age 5. We conducted two sets of regressions. In the first set — unadjusted estimates —, we regressed the outcome variable on the ownership of a mobile phone (model 1) or access to a mobile phone in the household (model 2), including covariates for the mother's age group when estimating fertility, and the child's age when estimating mortality. In the second set — adjusted estimates —, we added educational level, place of residence, wealth, and marital status as independent variables

For peer review only

to models 1 and 2. As for the categories of these independent variables, place of residence has two values: urban and rural; educational level takes three values: no education, primary education, and secondary and higher education; wealth has two values: poor (wealth quintiles 1 and 2) and not poor (wealth quintiles 3, 4 and 5); and, marital status takes two values: not in union (never married, not living together, divorced, or widowed) and in union (married or living together). We also included exposure (person-years calculated for women for fertility and for children for mortality) as an offset in all models. In addition, we computed raking weights to match the marginal distributions of the same sociodemographic variables and examine whether potential estimation biases could be addressed using post-stratification.

Differences in U5MR and TFR could be due to differences in women's behavior, which could be captured by mobile phone use, but not by sociodemographic variables. We proposed a series of logistic regressions to analyze the association between mobile phone ownership and access, and some variables connected to U5M and fertility. For mortality, we used six outcome variables. First, we modeled the likelihood of a mother having at least 4 antenatal care visits among those who gave birth in the 5 years before each survey. Second, the likelihood of a newborn receiving at least two signal functions as part of postnatal care (among births that occurred in the last 24 months). Third, the likelihood of a child being exclusively breastfed for the first 6 months (among the most recent birth in the last 6 months). Fourth, the likelihood of a child receiving eight basic immunizations among children aged 12 to 35 months at the time of the survey. The eight basic immunizations are one dose of BCG, three doses of DPT, three doses of Polio (excluding at birth), and one dose of Measles. Fifth, we analyzed the likelihood of skilled attendance at birth, which includes the presence of a doctor, nurse or nurse-midwife during delivery. All births in the 5-year period before the survey are considered. The last outcome variable connected with U5M that was analyzed was

For peer review only

the prevalence of underweight among children aged 0–5 at the time of the survey. A child is considered as being underweight if the weight-for-age z-score is below two standard deviations below the mean on the WHO Child Growth Standards. On the other hand, for fertility we used four outcome variables. First, the use of modern contraceptives. Second, sexual activity. We considered a woman to be sexually active when she reported having had sexual intercourse in the last month or being pregnant. Lastly, we analyzed duration of postpartum amenorrhea and duration of postpartum abstinence. For the latter two variables, we used a linear model since duration is measured as the number of months. Similar to the estimates of under-five fertility and mortality rates, unadjusted and adjusted models were estimated for all outcome variables.

We conducted this analysis using the programming language for statistical computing and graphics `R` [30]. We used the following packages: `rdhs` [31] for downloading the DHS datasets, `tidyverse` [32] for data manipulation and graphics, `demogsurv` [33] for preparing person-period tables stratified by the independent variables, `survey` [34] and `broom` [35] for econometric estimations and visualization, `anesrake` [36] for computing raking weights to match the marginal distributions of sociodemographic variables for testing for post-stratification, `MetBrewer` [37] for color palettes in our plots, and `kableExtra` [38] and `knitr` [39] for writing and editing the manuscript.

Patient and public involvement

Patients and/or the public were not involved in the design, or conduct, or reporting, or dissemination plans of this research.

For peer review only

RESULTS

Across the 34 surveys used, the percentage of women owning a mobile phone ranges from 23.6% in Burundi to 96.7% in Armenia. The percentage of women who have access to a mobile phone ranges from 55% in Burundi to 100% in the Maldives (see Appendix Table 1). The median ratio of the TFR calculated among non-owners to the TFR calculated among mobile phone owners is 1.48. Fertility is always higher among non-owners, with the lowest ratio at 1.04 in Bangladesh and the highest at 1.93 in Haiti. In absolute terms, these differences correspond to more than one child per woman in 25 of the 34 surveys studied here. Similarly, U5M is higher when calculated among non-owners, with the exception of South Africa 2016 and Zambia 2018-19. The median ratio of U5M calculated from reports of non-owners to that calculated from mothers with mobile phones is 1.29, and it ranges from 0.85 in South Africa to 2.5 in Albania. Differences in TFR and U5MR remain when access to a mobile phone is considered, but they are slightly reduced.

A series of logistic regressions (see Appendix Figures 1 to 5 in the supplementary material) conducted for each survey separately indicate that women with mobile phones differ from non-owners by age group, educational level, area of residence, wealth quintile, and marital status. Except for age, differences do not disappear when access to a mobile phone instead of ownership is considered. Compared to young adults aged 20–24, adolescents aged 15–19 are less likely to own a phone in all countries, although this is not true in 27 countries when we consider access rather than ownership. Age patterns of phone ownership vary by country for older women, but in most cases, differences are not statistically significant when access is considered.

Compared to women without education, women with primary education are significantly more likely to own a mobile phone in 30 surveys and women with secondary or higher education are

For peer review only

significantly more likely to own a mobile phone in all surveys. The number of surveys with a significant difference decreases, respectively, to 28 surveys and 30 surveys when access is considered. Women living in urban areas are significantly more likely to own a mobile phone in 29 surveys and to have access in 19 surveys. Odds ratios for women who own a mobile phone range from 0.94 (95% CI: 0.74 – 1.19) in South Africa to 5.42 (95% CI: 4.23 – 6.95) in Ethiopia, while for access, from 0.41 (95% CI: 0.25 – 0.67) in South Africa to 4.2 (95% CI: 3.12 – 5.67) in Sierra Leone.

In terms of household assets, the gaps are very large between women in the poorest households (quintiles 1 to 2) and those living in the most affluent households (quintiles 3 to 5). Unsurprisingly, women living in households with more amenities are between 1.17 (Maldives, 95% CI: 0.83 – 1.66) times and 4.85 (Burundi, 95% CI: 4.24 – 5.55) times more likely to own a mobile phone. As for access, the odds ratios range between 2.7 (Mali, 95% CI: 2.03 – 3.58) and 24.37 (Indonesia, 95% CI: 18.01 – 32.97). Women not in union are more likely to own a mobile phone than women in union in 19 countries, with odds ratios ranging from 0.56 (95% CI: 0.48 – 0.64) in Nepal to 1.78 (95% CI: 1.4 – 2.26) in the Philippines.

Figures 1 and 2 present the exponentiated coefficients of mobile phone ownership and access from Poisson regressions on U5M and fertility, respectively. Left panels of both figures show unadjusted estimates, while right panels contain adjusted estimates. Unadjusted coefficients suggest that in most countries, ownership is associated with lower odds of experiencing a birth or the death of a child under five years of age. When considering U5M estimates and mobile phone ownership, coefficients are statistically significant in all countries but nine (left panel of Figure 1). However, in most countries differences by ownership are no longer significant once we adjust for marital status, education, wealth, and place of residence (right panel of Figure 1). Women who own a

For peer review only

mobile phone are less likely to lose a child in only four countries (Cameroon, Gambia, Nepal, and Nigeria) after adjusting for sociodemographic characteristics. Overall, U5M is not statistically associated with owning or having access to a mobile phone, after controlling for basic sociodemographic characteristics. U5MR estimates obtained using post-stratification weights from mobile phone owners are in line with these findings (see right panel of Figure 3). Only in Nigeria is it not possible to reproduce actual U5MR using ex-post weightings. Results for access are presented in Appendix Figure 6.

Unadjusted coefficients from the Poisson regressions on fertility for mobile phone owners are also statistically significant in 30 countries (left panel of Figure 2). However, in contrast to what we observed for U5M, the negative effect of owning a phone persists for fertility in most countries even after controlling for covariates (right panel of Figure 2). Ethiopia is the only survey where this association loses significance after controlling for sociodemographic characteristics. When access is considered, the effect is not significant in five additional countries (Burundi, Cameroon, Mali, Pakistan, and Senegal). In most countries, ownership or access is associated with lower fertility, even after controlling for basic sociodemographic characteristics. The use of post-stratification weights based on the marginal distribution of sociodemographic variables of mobile phone owners shows that it is possible to reproduce the observed TFR in a total of 22 countries (see left panel of Figure 3). However, this also means that post-stratification cannot provide unbiased fertility estimates in one-third of the countries included in our study. When access is considered (see Appendix Figure 6), there are no statistical differences with the TFRs observed in the general population. As mobile phone access is very high, marginal distributions of the sociodemographic variables of women with access are very similar to that of the whole population so post-stratification plays a small role in this case.

For peer review only

In Figure 4, we present the estimates of the association between ownership and variables connected to fertility. Results for access to mobile phones are presented in Appendix Figure 7. We observe statistically significant differences in use of modern contraceptives in 23 surveys. In most of them, owners are more likely to use contraceptives. After controlling for covariates, differences remain in 20 surveys. When access is considered, the number of surveys is reduced to 16. In contrast, mobile phone owners are less likely to be sexually active in 14 surveys, even after controlling for sociodemographic characteristics. However, differences disappear in most surveys when access is considered. Postpartum amenorrhea periods are significantly shorter among owners in 19 surveys, although only in 8 surveys after including controls. In contrast, owners have significantly longer postpartum abstinence periods in 15 surveys, but only in 8 surveys after adjusting for controls.

Analyses of the association between ownership and variables connected to U5M are presented in Figure 5. Results for access are in Appendix Figure 8. Owners are more likely to have had at least four antenatal care visits in all but 3 surveys. Differences are not statistically significant in 6 surveys after controlling for covariates. Likewise, births born to mothers who own a mobile phone are more likely to have received at least two signal functions as part of postnatal check-ups in 28 surveys, although the number of surveys is reduced to 18 after controlling for covariates. In contrast, owners are more likely to exclusively breastfeed for the first 6 months to newborns in 4 surveys, and less likely in 4 other surveys, although these differences disappear in virtually all surveys after controlling for covariates. Children born to owners are more likely to have received basic immunization in only 5 surveys after adjusting for controls. In contrast, children born to owners are more likely to have been attended by health professionals at delivery in 27 surveys. Owners are less likely to have underweight children in more than half of the surveys, even after controlling for

For peer review only

covariates. Differences disappear for most variables when access is considered. Exceptions are antenatal care, the presence of a health professional at delivery, and, to a lesser extent, underweight.

DISCUSSION

We analyzed the social gradient in mobile phone ownership and access in 34 LMIC, and evaluated the implications for fertility and U5M estimates derived from MPS. Consistent with previous research [16–19], we found that well educated, better-off, and urban women are more likely to own and have access to a mobile phone. These differences in the use of mobile phones could bias demographic estimates from MPS.

Fertility and U5M are higher in less advantaged populations [27,28], groups which are underrepresented in MPS. Therefore, MPS are likely to underestimate true levels of fertility and U5M in the wider population. This is an important first conclusion of this study: MPS will underestimate U5M and total fertility. However, the negative association between U5M and mobile phone ownership disappears once we control for a number of sociodemographic characteristics that can be easily collected in MPS. This is a second, and methodologically, important finding of our study as it suggests that any downward bias in U5M estimates from MPS due to selectivity of respondents can be corrected using a post-stratification procedure. More substantively, our results contradict the hypothesis that at the aggregate level the expansion of mobile phones is associated with lower child mortality as this association seems spurious [2].

Surprisingly, the negative association between mobile phone ownership and fertility persists in many of the countries included in this study even after controlling for sociodemographic characteristics. From a methodological standpoint, this suggest that fertility rates could be biased downwards if they are estimated from MPS, even after post-stratification. This third important

For peer review only

finding is consistent with previous research arguing that mobile phone use is associated with higher rates of contraceptive use [2,7,20]. Our results suggest that the use of modern contraceptives is more likely among owners in two out of five surveys analyzed in this study, even after controlling for wealth, education, place of residence, marital status and age. Further, there are some apps that track menstrual periods and send alerts on ovulation days, allowing for better birth control [2,20]. Even less sophisticated models of mobile phones — i.e., not smartphones — include calendars that can be used to schedule reminders for tracking the menstrual cycle or taking contraceptives. In addition, we found that mobile phone owners are less likely to be sexually active in most of the surveys. Higher odds of contraceptive use and lower odds of sexual activity lead to lower fertility rates. It is worth noting that one difference between fertility and mortality is that fertility outcomes can be more easily controlled based on preferences than exposure to illness and injuries. These fertility preferences may be correlated with, but are not fully captured by, the sociodemographic characteristics.

Our results also show that there are no significant differences between owners and non-owners in terms of exclusive breastfeeding. This does not contradict what Lefevre and colleagues [26] found, as they estimated their results using differences in means without controlling for covariates while we have used the association of exclusive breastfeeding conditional on mobile phone ownership and other sociodemographic variables. Our findings can help to explain why the differences in U5MR by ownership disappear when controlling for covariates, since the health benefits from exclusive breastfeeding and basic immunization could be offsetting the negative effects of no or few prenatal and postnatal checkups, or low birth weight.

As for logistical implications for fieldwork, MPS should preferably be conducted at times when household heads are likely to be present so that women in the household who do not own a mobile

For peer review only

phone can be surveyed. But biases related to the selection of phone users may remain even after using this approach. A different way of accounting for selection bias (apart from post-stratification), is to make use of sampling quotas (i.e., pre-stratification). This is usually based on a set of simple screening questions like age, rural/urban residence and educational attainment. However, pre-stratification might be more expensive and it will require greater effort to reach respondents with the desired attributes. Finally, from the perspective of survey designers, conducting complementary face-to-face surveys could be suitable for reaching populations that are typically difficult to cover through MPS. It should be noted that our study evaluates selection bias due to sampling frame limitations (mobile phone access/ownership) and not due to non-response. Non-response is often high in MPS, and considerably higher than in face-to-face surveys. We did not consider either the recall errors which could be more systematic in MPS due to lower engagement of the respondents.

In conclusion, while MPS are a cheaper alternative or complement to classical face-to-face surveys for estimating demographic and health indicators, estimates derived from MPS cannot be taken at face value owing to the unequal ownership and access of mobile phones in many LMIC. In this study, we have shown that under-five mortality estimates can be corrected using a post-stratification on a number of sociodemographic background characteristics to match nationally representative estimates. Adjusting fertility estimates is less straightforward, most likely because fertility decision making is an expression of individual preferences in addition to background characteristics and living conditions.

For peer review only

Contributors

DASP and BM led the conception and design of the study. DASP and BM analyzed the data, produced the estimates and drafted the manuscript. AMO, JB and GR reviewed the estimates and helped with the interpretation of the results. All authors critically reviewed the manuscript and approved the final version of the paper. DASP is the guarantor of the overall content of this manuscript and had final responsibility for the decision to submit for publication.

Competing interests

We declare no competing interests.

Funding

This study was supported by the Bill & Melinda Gates Foundation grant number INV-023211. The funders of the study had no role in the study design, data analysis, data interpretation, or writing of the report.

Data availability statement

All datasets are publicly and freely available on the DHS Program website. https://dhsprogram.com/data/

Patient consent for publication

Not applicable.

Ethics Approval

Not applicable since the study was a secondary analysis of publicly available data.

For peer review only

References

1 Gibson DG, Pereira A, Farrenkopf BA, Labrique AB, Pariyo GW, Hyder AA. Mobile phone surveys for collecting population-level estimates in low-and middle-income countries: A literature review. *Journal of Medical Internet Research* 2017; **19**: e139.

2 Rotondi V, Kashyap R, Pesando LM, Spinelli S, Billari FC. Leveraging mobile phones to attain sustainable development. *Proceedings of the National Academy of Sciences* 2020; **117**: 13413–20.

3 Donner J. The use of mobile phones by microentrepreneurs in Kigali, Rwanda: Changes to social and business networks. *Information Technologies and International Development* 2007; **3**: 3–19.

4 Jagun A, Heeks R, Whalley J. The impact of mobile telephony on developing country micro-enterprise: A Nigerian case study. *Information Technologies and International Development* 2008; **4**: 47–65.

5 Firchow P, Ginty RM. Including hard-to-access populations using mobile phone surveys and participatory indicators. *Sociological Methods & Research* 2020; **49**: 133–60.

6 Kempf AM, Remington PL. New challenges for telephone survey research in the twenty-first century. *Annual Review of Public Health* 2007; **28**: 113–26.

7 Greenleaf AR, Gadiaga A, Guiella G, *et al.* Comparability of modern contraceptive use estimates between a face-to-face survey and a cellphone survey among women in Burkina Faso. *PLOS ONE* 2020; **15**: e0231819.

8 Kuehne A, Lynch E, Marshall E, *et al.* Mortality, morbidity and health-seeking behaviour during the Ebola epidemic 2014–2015 in Monrovia results from a mobile phone survey. *PLoS Neglected Tropical Diseases* 2016; **10**: 1–2.

9 Adjiwanou V, Alam N, Alkema L, *et al.* Measuring excess mortality during the COVID-19 pandemic in low- and lower-middle income countries: The need for mobile phone surveys. 2020. DOI:10.31235/osf.io/4bu3q.

10 Chasukwa M, Choko AT, Muthema F, *et al.* Collecting mortality data via mobile phone surveys: A non-inferiority randomized trial in Malawi. *PLOS Global Public Health* 2022; **2**: e0000852.

11 Dabalen A, Etang A, Hoogeveen J, Mushi E, Schipper Y, Engelhardt J von. Mobile phone panel surveys in developing countries: A practical guide for microdata collection. Washington, DC: World Bank, 2016.

12 Blumenstock JE, Hall MG, Eagle N. Divided we call: Disparities in access and use of mobile phones in Rwanda. *Information Technologies and International Development* 2012; **8**: 1–6.

For peer review only

13 Huyer S, Hafkin N, Ertl H, Dryburgh H. Women in the Information Society. In: The digital divide to digital opportunities: Measuring infostates for development. NRC Press, 2005: 135–96.

14 Murphy LL, Priebe AE. "My co-wife can borrow my mobile phone!": Gendered geographies of cell phone usage and significance for rural Kenyans. *Gender, Technology and Development* 2011; **15**: 1–23.

15 Huyer S, Carr M. Information and Communication Technologies: A priority for women. *Gender, Technology and Development* 2002; **6**: 85–100.

16 L'Engle K, Sefa E, Adimazoya EA, *et al.* Survey research with a random digit dial national mobile phone sample in Ghana: Methods and sample quality. *PLoS ONE* 2018; **13**: e0190902.

17 Aker JC, Mbiti IM. Mobile phones and economic development in Africa. *Journal of Economic Perspectives* 2010; **24**: 207–32.

18 Wesolowski A, Eagle N, Noor AM, Snow RW, Buckee CO. Heterogeneous mobile phone ownership and usage patterns in Kenya. *PLos ONE* 2012; **7**: e35319.

19 Wakadha H, Chandir S, Victor E, *et al.* The feasibility of using mobile-phone based SMS reminders and conditional cash transfers to improve timely immunization in rural Kenya. *Vaccine* 2013; **31**: 987–93.

20 Billari F, Rotondi V, Trinitapoli J. Mobile phones, digital inequality and fertility: Longitudinal evidence from Malawi. *Demographic Research* 2020; **42**: 1057–96.

21 Smith C, Gold J, D Ngo T, Sumpter C, Free C. Mobile phone-based interventions for improving contraception use. London: Cochrane Database of Systematic Reviews, 2015.

22 Coleman J, Bohlin KC, Thorson A, *et al.* Effectiveness of an SMS-based maternal mHealth intervention to improve clinical outcomes of HIV-positive pregnant women. *AIDS Care* 2017; **29**: 890–7.

23 Hall CS, Fottrell E, Wilkinson S, Byass P. Assessing the impact of mHealth interventions in low- and middle-income countries - what has been shown to work? *Global Health Action* 2014; **7**. DOI:10.3402/gha.v7.25606.

24 Lund S, Nielsen BB, Hemed M, *et al.* Mobile phones improve antenatal care attendance in Zanzibar: A cluster randomized controlled trial. *BMC Pregnancy and Childbirth* 2014; **14**: 1–0.

25 Noordam AC, Kuepper BM, Stekelenburg J, Milen A. Improvement of maternal health services through the use of mobile phones. *Tropical Medicine and International Health* 2011; **16**: 622–6.

26 LeFevre AE, Shah N, Harrisson Bashingwa JJ, George AS, Mohan D. Does women's mobile phone ownership matter for health? Evidence from15 countries. *BMJ Global Health* 2020; **5**: e002524.

For peer review only

27 Finlay JE, Mejía-Guevara I, Akachi Y. Inequality in total fertility rates and the proximate determinants of fertility in 21 sub-Saharan African countries. *PLoS ONE* 2018; **13**: e0203344.

28 Rogers RG, Wofford S. Life expectancy in less developed countries: Socioeconomic development or public health? *Journal of Biosocial Science* 2008; **21**: 245–52.

[dataset] 29 ICF. Data from: Demographic and Health Surveys. The DHS Program, 2022. http://www.dhsprogram.com/Data/.

30 R Core Team. R: A language and environment for statistical computing. R version 4.2.1 patched. Vienna, Austria: R Foundation for Statistical Computing, 2022 https://www.R-project.org/.

31 Watson OJ, FitzJohn R, Eaton JW. Rdhs: An R package to interact with the Demographic and Health Surveys (DHS) Program datasets. *Wellcome Open Research* 2019; **4**: 103–25.

32 Wickham H, Averick M, Bryan J, *et al.* Welcome to the Tidyverse. *Journal of Open Source Software* 2019; **4**: 1686–91.

33 Eaton J, Masquelier B. Demogsurv: Demographic analysis of DHS and other household surveys. R package version 0.2.5. 2021 https://github.com/mrc-ide/demogsurv.

34 Lumley T. Analysis of complex survey samples. *Journal of Statistical Software* 2004; **9**: 1–9.

35 Robinson D, Hayes A, Couch S. broom: Convert statistical analysis objects into tidy tibbles. R package version 0.7.12. 2022 https://CRAN.R-project.org/package=broom.

36 Pasek J. Anesrake: ANES Raking Implementation. R package version 0.80. 2018 https://CRAN.R-project.org/package=anesrake.

37 Mills BR. MetBrewer: Color palettes inspired by works at the Metropolitan Museum of Art. R package version 0.2.0. 2022 https://CRAN.R-project.org/package=MetBrewer.

38 Zhu H. kableExtra: Construct complex table with 'kable' and pipe syntax. R package version 1.3.4. 2021 https://CRAN.R-project.org/package=kableExtra.

39 Xie Y. Knitr: A general-purpose package for dynamic report generation in R. R package version 1.37. 2021 https://yihui.org/knitr/.

For peer review only

Figure 1: Unadjusted and adjusted estimates of the effect of owning and having access to a mobile phone on under-five mortality from Poisson regressions.

Figure 2: Unadjusted and adjusted estimates of the effect of owning and having access to a mobile phone on fertility from Poisson regressions.

Figure 3: Under-five mortality rate and total fertility rate estimates testing for post-stratification weights from women who own a mobile phone.

Figure 4: Estimates of the difference in current use of modern contraceptives (MCU), sexual activity (SexAct), duration of postpartum amenorrhea (Amen), and duration of postpartum abstinence (Abst), between owners and non-owners of a mobile phone.

Figure 5: Estimates of the difference in exclusive breastfeeding (BrFeed), births who had at least 4 antenatal care visits (ANC), newborns who received at least 2 signal functions (PNC), children who received basic immunization (Vax), births delivered by skilled professionals (Skill), children who are underweight (UnderW), between owners and non-owners of a mobile phone.

For peer review only

Figure 1: Unadjusted and adjusted estimates of the effect of owning and having access to a mobile phone on under-five mortality from Poisson regressions.

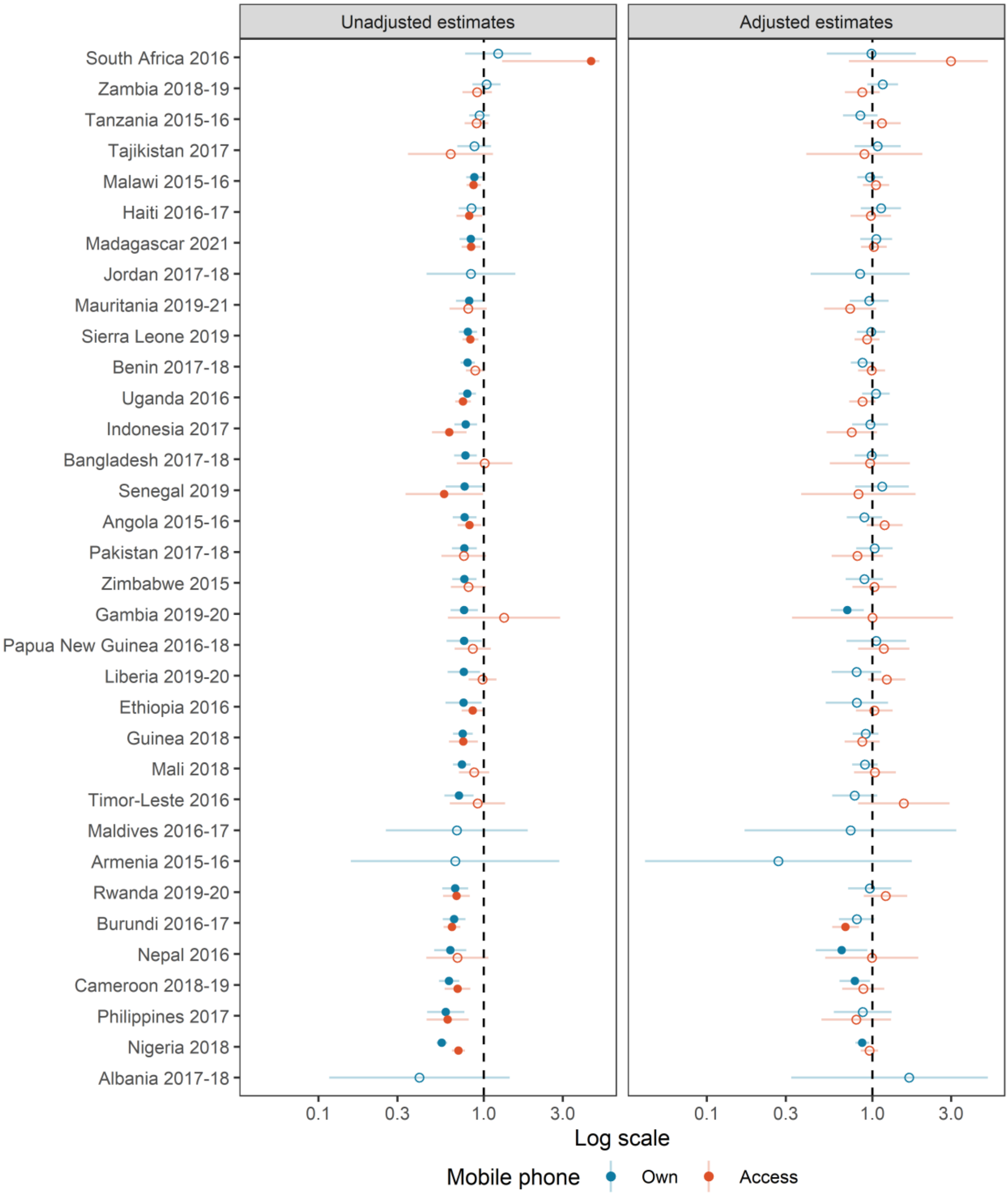

Note: Estimates for access to a mobile phone are not presented for Albania, Armenia, Jordan and Maldives, as access is almost 100%. Fill dots mean that estimates are statistically significant at the 95% confidence level, while empty dots mean that estimates are not statistically significant.

Figure 2: Unadjusted and adjusted estimates of the effect of owning and having access to a mobile phone on fertility from Poisson regressions.

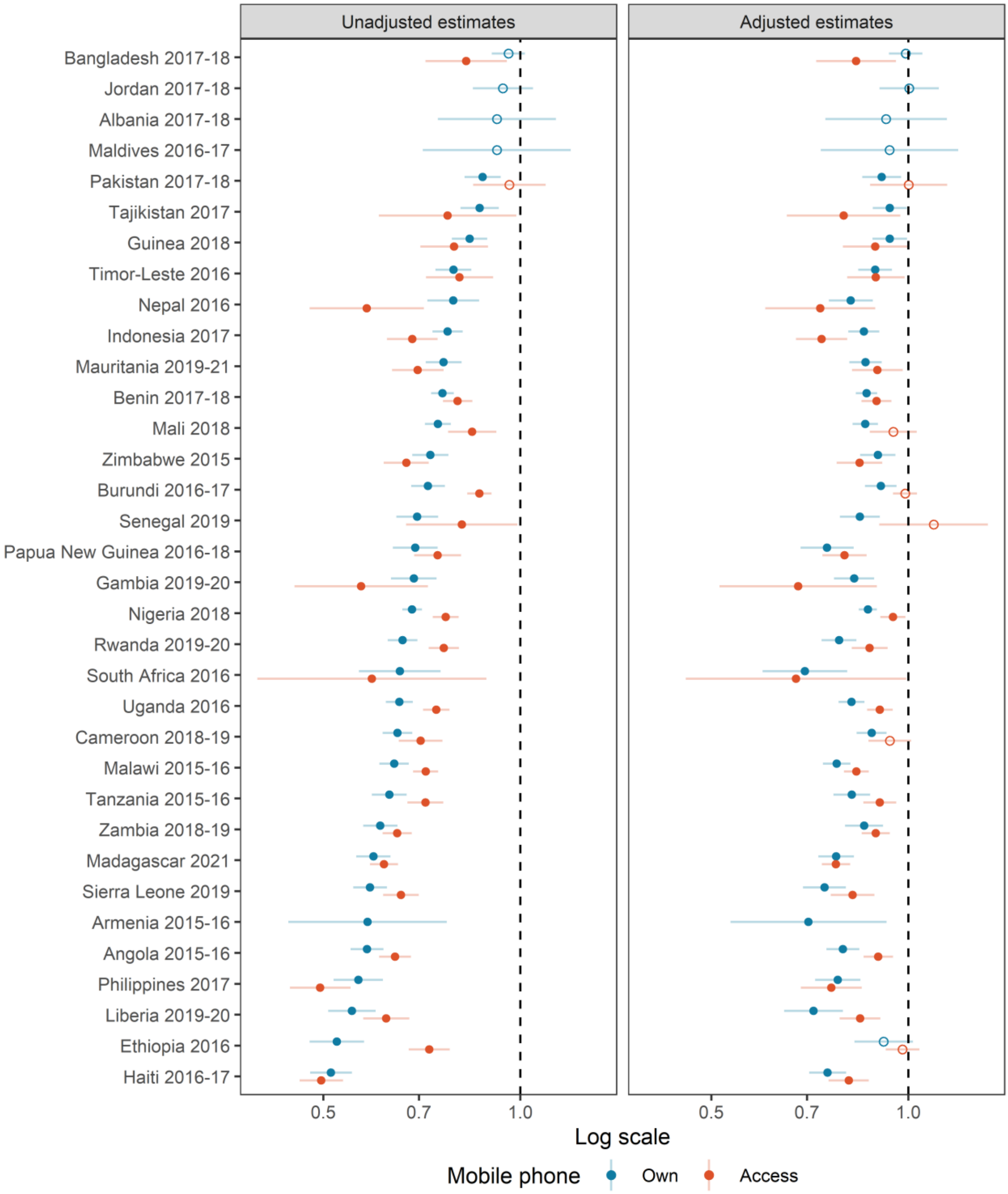

Note: Estimates for access to a mobile phone are not presented for Albania, Armenia, Jordan and Maldives, as access is almost 100%. Fill dots mean that estimates are statistically significant at the 95% confidence level, while empty dots mean that estimates are not statistically significant.

Figure 3: Under-five mortality rate and total fertility rate estimates testing for post-stratification weights from women who own a mobile phone.

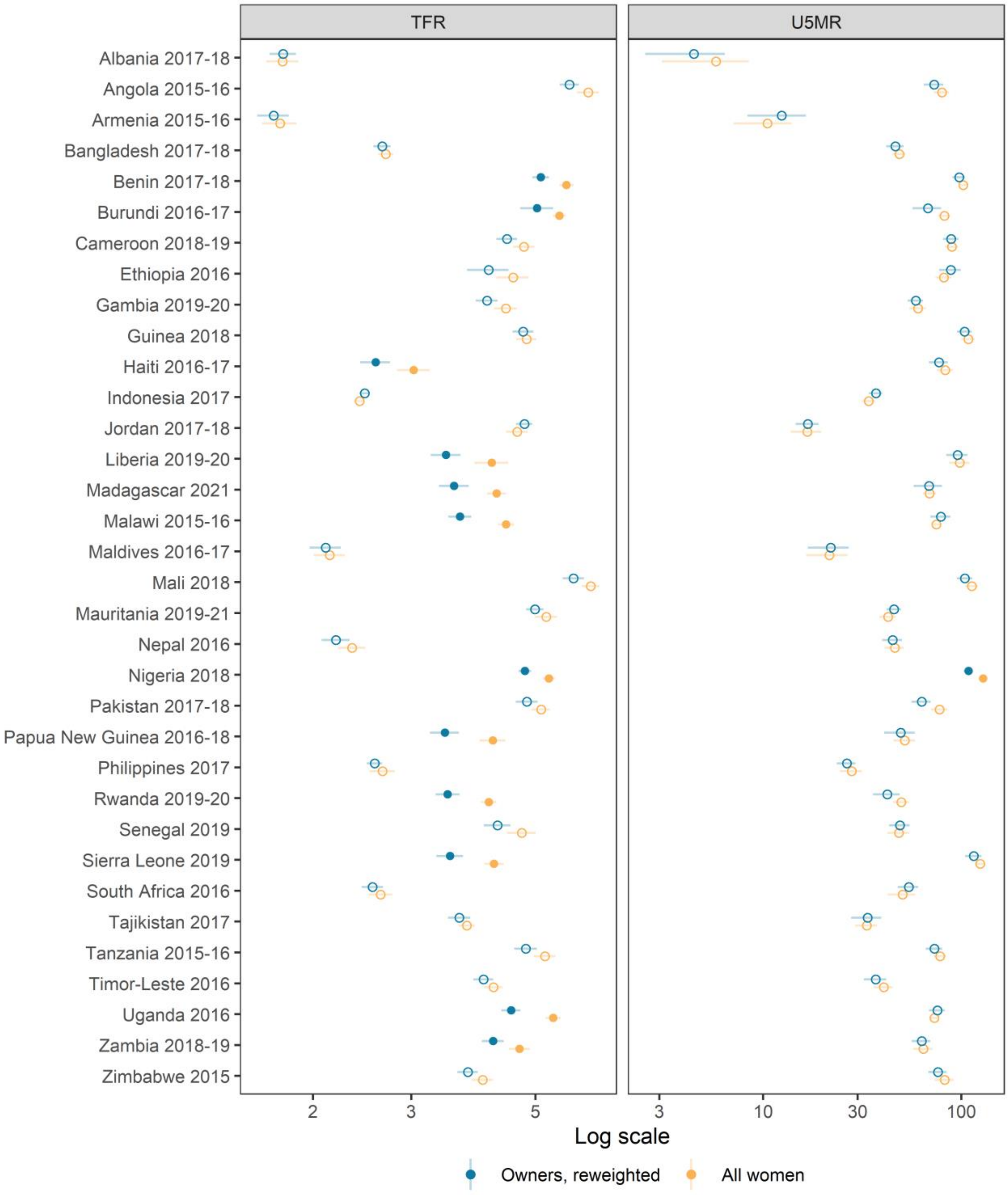

Note: Fill dots mean that the difference between the actual rate and the re-weighted rate is statistically significant at the 95% confidence level, while empty dots mean that the difference is not statistically significant.

Figure 4: Estimates of the difference in current use of modern contraceptives (MCU), sexual activity (SexAct), duration of postpartum amenorrhea (Amen), and duration of postpartum abstinence (Abst), between owners and non-owners of a mobile phone.

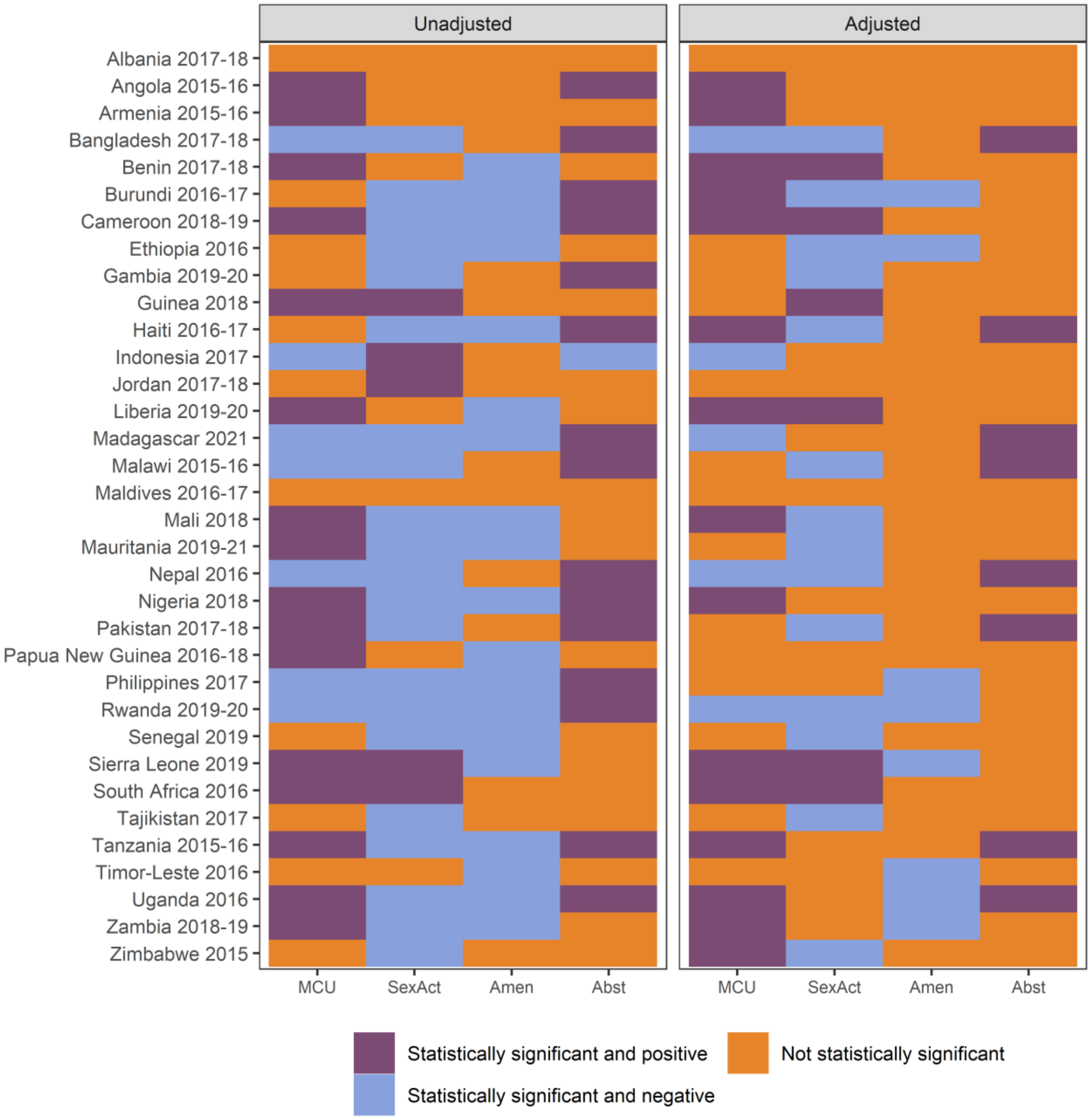

Figure 5: Estimates of the difference in exclusive breastfeeding (BrFeed), births who had at least 4 antenatal care visits (ANC), newborns who received at least 2 signal functions (PNC), children who received basic immunization (Vax), births delivered by skilled professionals (Skill), children who are underweight (UnderW), between owners and non-owners of a mobile phone.

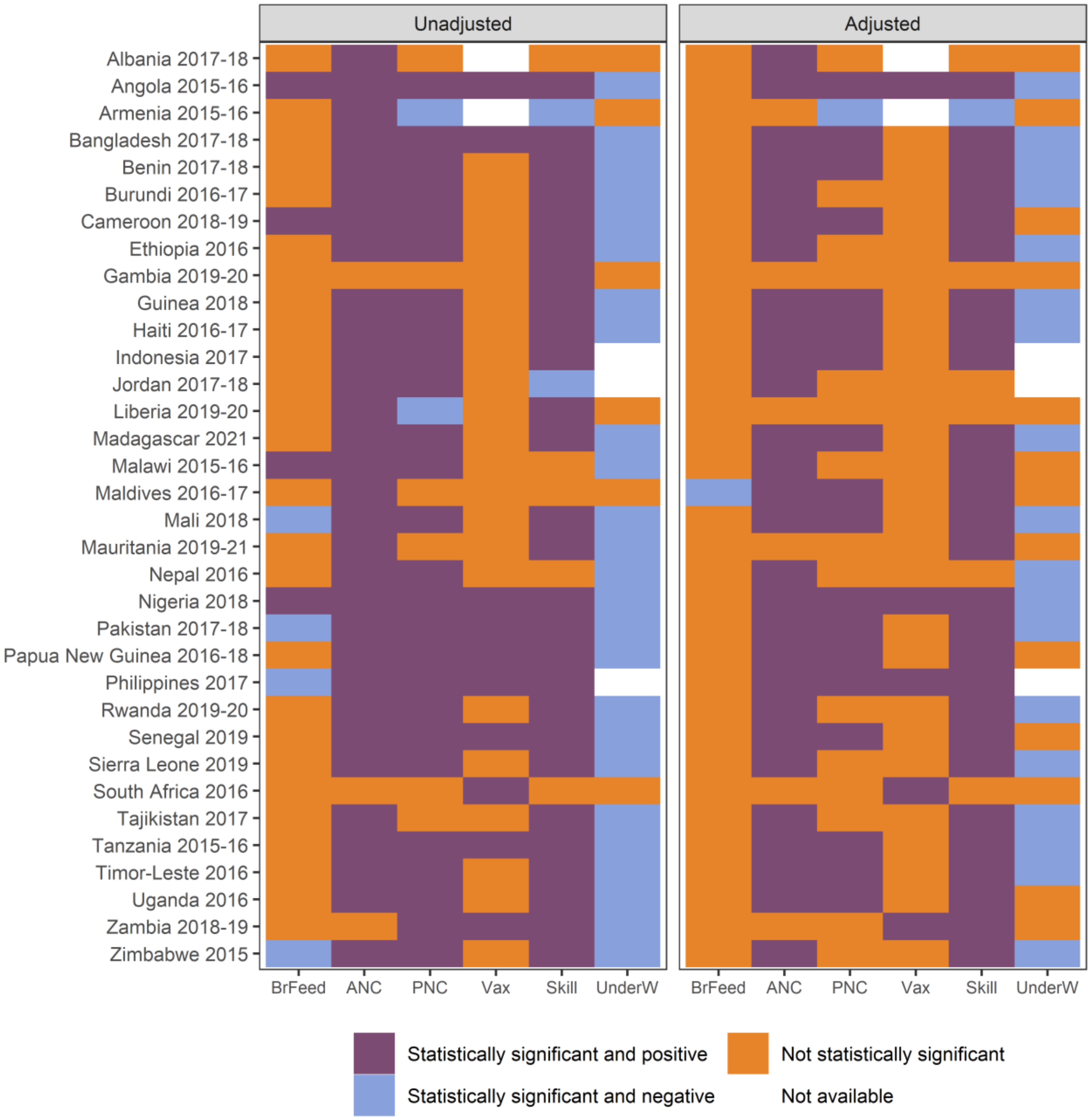

Appendix Table 1: DHS including information on mobile phone ownership and access, and total fertility rate (TFR) for the 3-year period before the survey and under-five mortality rate (U5MR) for the 10-year period before the survey by mobile phone status.

		Mobile phone ownership					Access to a mobile phone				
		Owns a mobile phone			Does not own a mobile phone		Can access a mobile phone			Cannot access a mobile phone	
Survey	Sample size	% of women	TFR	U5MR	TFR	U5MR	% of women	TFR	U5MR	TFR	U5MR
Albania 2017-18	10,970	90.1	1.8	5.1	2.0	12.7	99.3	1.8	5.7	3.0	18.8
Angola 2015-16	14,379	51.2	4.7	67.9	8.0	89.1	75.3	5.5	74.0	8.4	92.3
Armenia 2015-16	6,116	96.7	1.7	10.3	3.0	15.5	99.8	1.7	10.5	*NA*	*NA*
Bangladesh 2017-18	20,127	60.2	2.2	43.9	2.3	56.6	97.2	2.2	48.7	2.7	46.7
Benin 2017-18	15,928	51.1	5.0	91.5	6.7	113.2	90.4	5.5	100.0	7.1	117.0
Burundi 2016-17	17,269	23.6	4.4	58.8	5.9	88.3	55.0	5.3	65.3	5.9	99.0
Cameroon 2018-19	13,901	63.2	4.0	72.1	6.4	115.4	91.5	4.6	85.0	6.0	126.6
Ethiopia 2016	15,683	27.3	2.8	65.8	5.2	84.1	62.1	4.0	74.9	5.5	88.7
Gambia 2019-20	11,865	76.4	4.1	56.0	6.1	75.2	99.6	4.4	60.3	8.1	69.3
Guinea 2018	10,874	68.9	4.6	98.7	5.5	129.6	94.1	4.8	105.9	6.0	141.9
Haiti 2016-17	14,371	57.1	2.3	75.9	4.4	89.8	86.3	2.7	78.1	5.3	98.9
Indonesia 2017	49,569	78.5	2.3	31.9	3.2	40.5	96.3	2.4	33.1	3.7	49.8
Jordan 2017-18	14,689	91.8	2.7	16.5	2.9	19.2	99.2	2.7	16.8	3.0	7.9
Liberia 2019-20	8,065	46.7	2.9	84.0	5.4	107.9	78.6	3.7	96.8	5.9	101.6
Madagascar 2021	18,869	34.6	3.0	60.1	5.0	72.5	61.3	3.2	63.7	5.0	71.7

For peer review only

Malawi 2015-16	24,562	32.8	3.2	67.7	5.1	77.9	62.4	3.8	69.9	5.4	81.4
Maldives 2016-17	7,699	95.5	2.1	21.5	2.4	27.7	100.0	2.1	21.6	*NA*	*NA*
Mali 2018	10,519	58.3	5.5	97.1	7.4	130.1	94.3	6.2	111.8	7.4	126.3
Mauritania 2019-21	15,714	76.7	4.9	40.6	6.8	49.5	96.1	5.1	41.7	7.5	57.7
Nepal 2016	12,862	72.6	2.2	40.3	3.1	62.8	97.3	2.3	45.3	4.0	64.9
Nigeria 2018	41,821	55.3	4.4	93.0	6.5	163.6	92.6	5.2	123.3	6.6	181.5
Pakistan 2017-18	12,363	39.2	3.2	64.4	3.8	85.1	95.4	3.5	77.0	4.1	88.8
Papua New Guinea 2016-18	15,141	34.3	3.2	40.6	4.7	56.2	66.6	3.7	48.5	5.1	56.4
Philippines 2017	25,074	85.8	2.5	24.6	4.3	41.4	95.5	2.5	27.0	5.2	36.8
Rwanda 2019-20	14,634	47.9	3.4	38.6	5.1	59.3	79.1	3.9	44.4	5.1	64.4
Senegal 2019	8,649	69.7	4.2	44.3	6.3	57.1	98.9	4.7	47.5	5.9	98.7
Sierra Leone 2019	15,574	42.6	3.1	110.3	5.2	132.6	81.4	3.9	118.2	5.9	142.0
South Africa 2016	8,514	91.2	2.6	51.2	4.1	43.7	98.8	2.6	51.2	4.1	10.5
Tajikistan 2017	10,718	53.7	3.5	31.5	4.1	35.6	98.1	3.7	32.6	5.1	64.4
Tanzania 2015-16	13,266	52.2	4.1	75.4	6.7	80.8	86.2	4.9	75.6	6.8	88.8
Timor-Leste 2016	12,607	65.6	3.9	35.5	5.0	49.4	94.3	4.2	40.8	5.1	38.1
Uganda 2016	18,506	45.5	4.3	65.2	6.7	79.7	81.8	5.1	68.6	6.8	89.7
Zambia 2018-19	13,683	53.0	3.7	65.4	6.1	63.4	81.2	4.2	62.7	6.6	69.1
Zimbabwe 2015	9,955	69.5	3.7	75.6	5.1	99.3	92.2	3.9	79.8	5.8	102.2

Note: Fertility and mortality rates for women who cannot access a mobile phone cannot be computed for Armenia and Maldives due to the small sample size.

For peer review only

Appendix Figure 1.1: Effect of the 15-19 age group on the odds of owning and having access to a mobile phone from binomial logistic regressions (20-24 age group is the reference). X-axis in log scale.

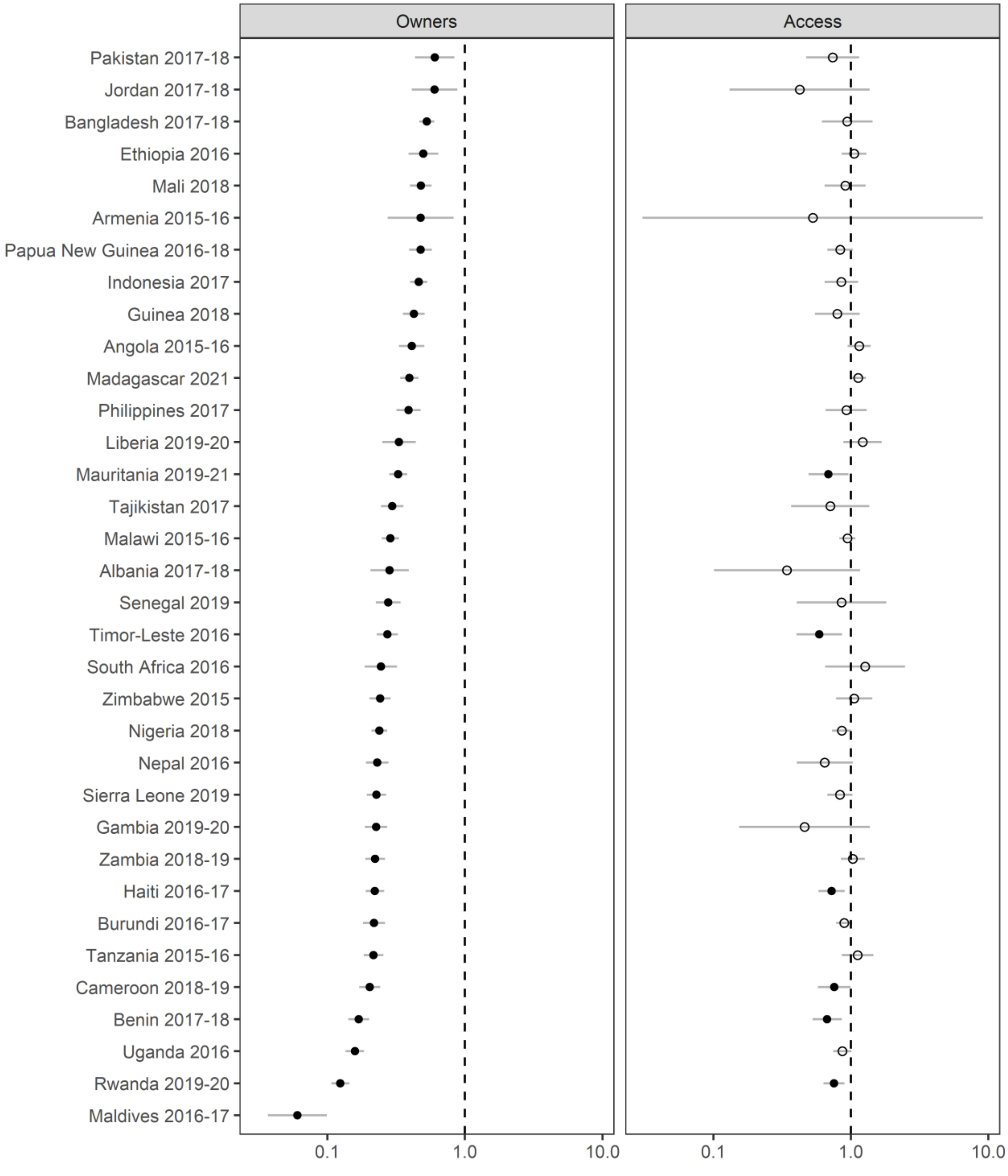

Note: Estimates for access to a mobile phone cannot be computed for Maldives due to small sample size. Fill dots mean that estimates are statistically significant at the 95% confidence level, while empty dots mean that estimates are not statistically significant.

Appendix Figure 1.2: Effect of the 25-29 age group on the odds of owning and having access to a mobile phone from binomial logistic regressions (20-24 age group is the reference). X-axis in log scale.

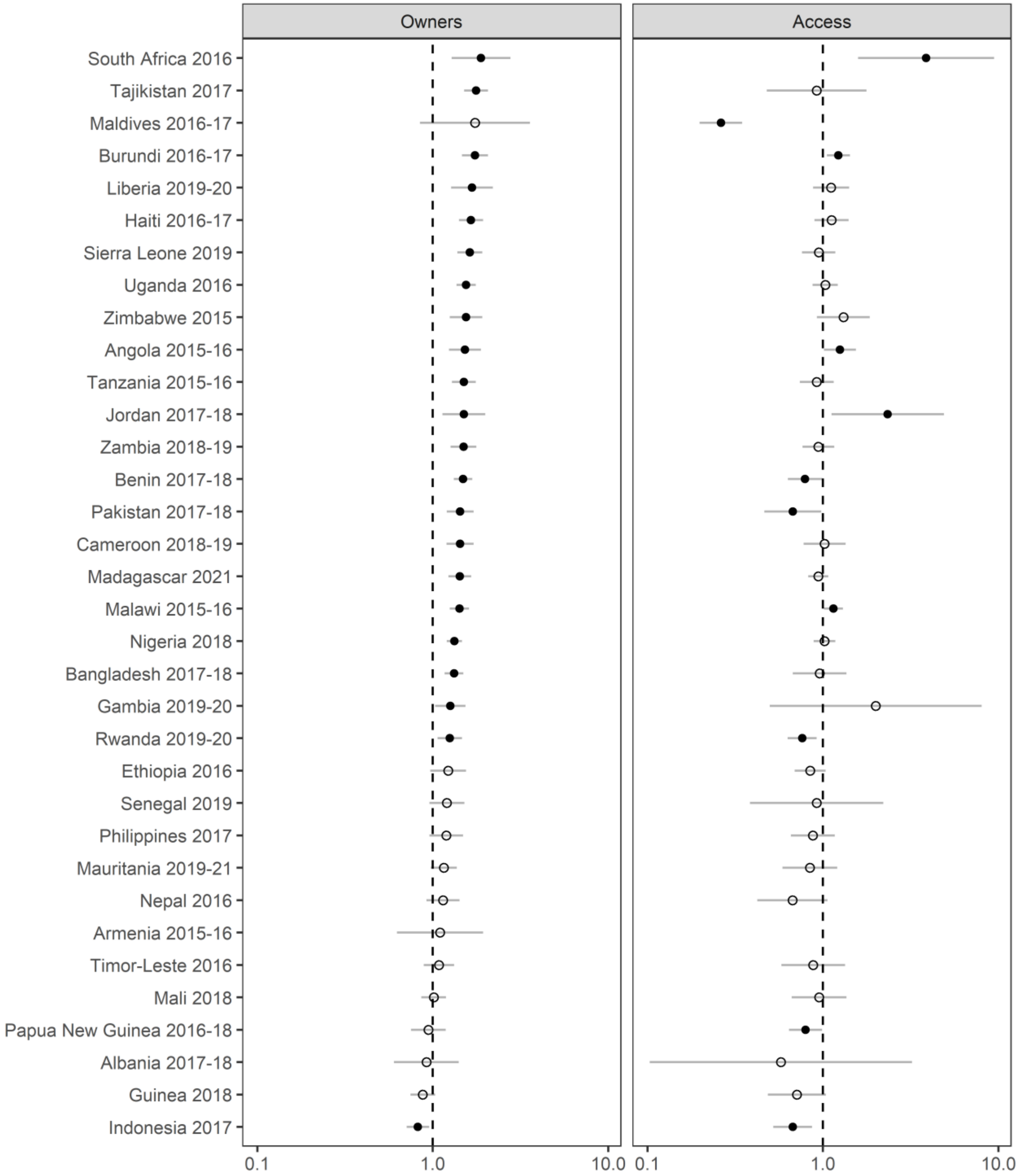

Note: Estimates for access to a mobile phone cannot be computed for Armenia due to small sample size. Fill dots mean that estimates are statistically significant at the 95% confidence level, while empty dots mean that estimates are not statistically significant.

Appendix Figure 1.3: Effect of the 30-34 age group on the odds of owning and having access to a mobile phone from binomial logistic regressions (20-24 age group is the reference). X-axis in log scale.

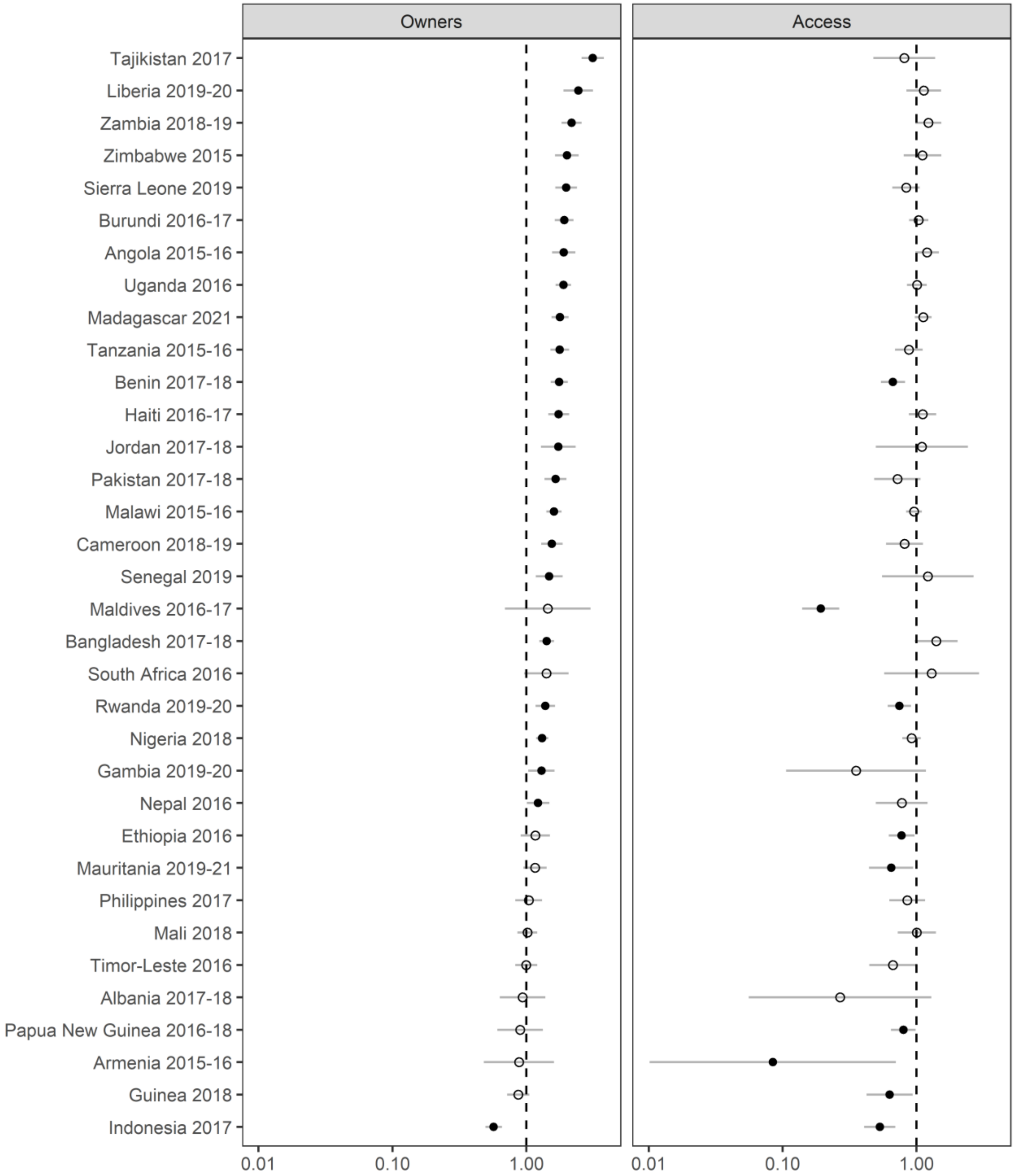

Note: Fill dots mean that estimates are statistically significant at the 95% confidence level, while empty dots mean that estimates are not statistically significant.

Appendix Figure 1.4: Effect of the 35-39 age group on the odds of owning and having access to a mobile phone from binomial logistic regressions (20-24 age group is the reference). X-axis in log scale.

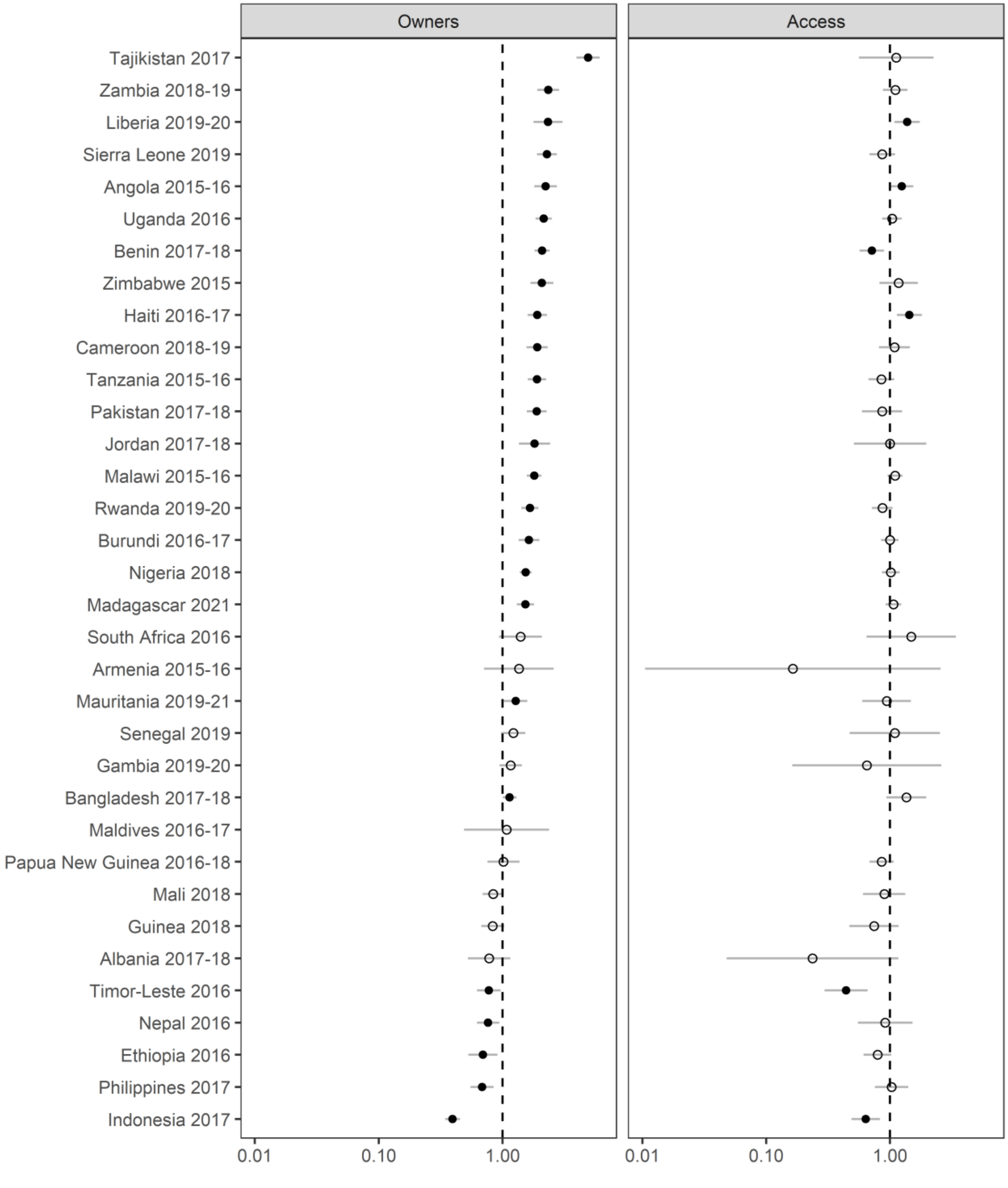

Note: Estimates for access to a mobile phone cannot be computed for Maldives due to small sample size. Fill dots mean that estimates are statistically significant at the 95% confidence level, while empty dots mean that estimates are not statistically significant.

Appendix Figure 1.5: Effect of the 40-44 age group on the odds of owning and having access to a mobile phone from binomial logistic regressions (20-24 age group is the reference). X-axis in log scale.

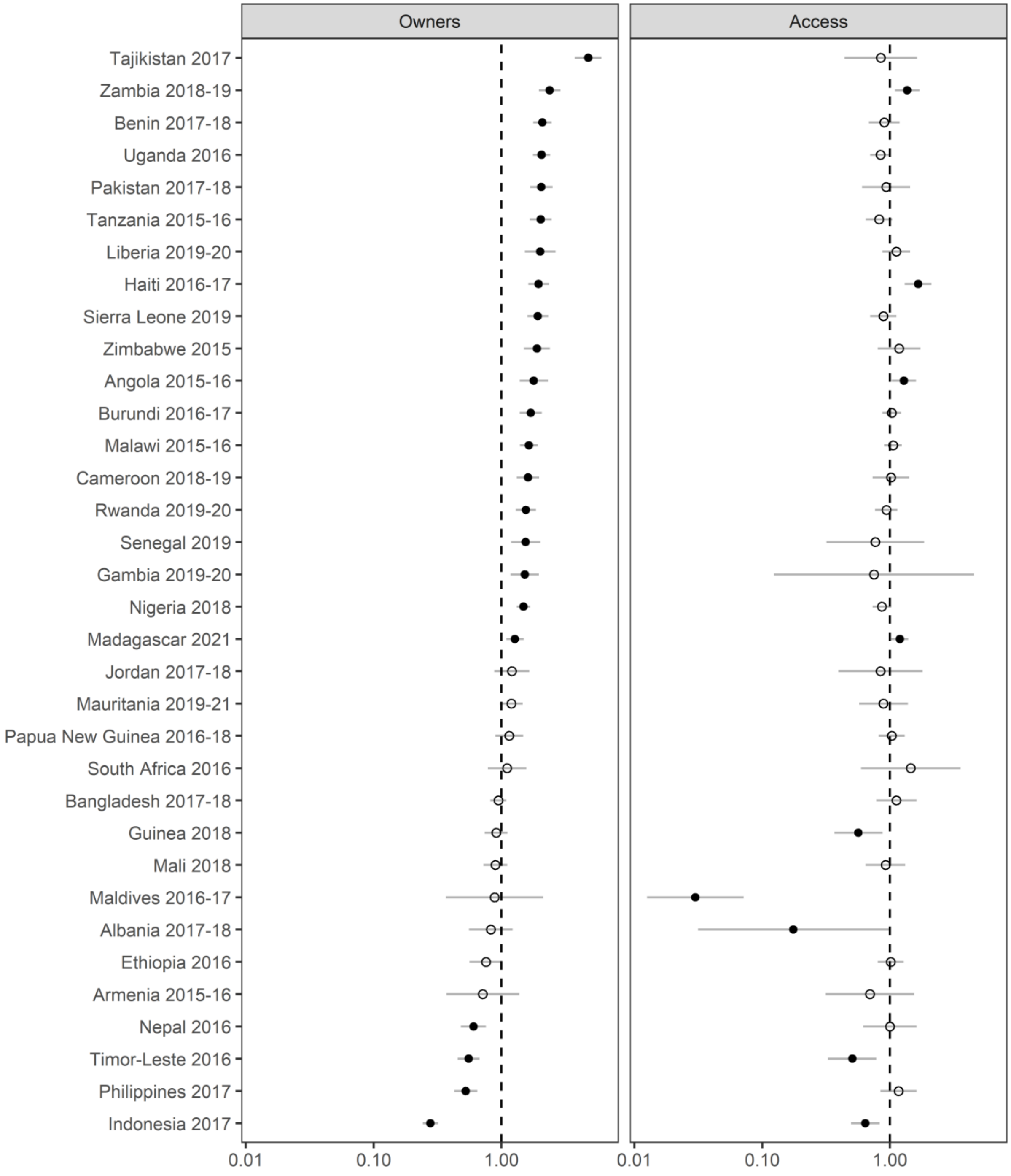

Note: Fill dots mean that estimates are statistically significant at the 95% confidence level, while empty dots mean that estimates are not statistically significant.

Appendix Figure 1.6: Effect of the 45-49 age group on the odds of owning and having access to a mobile phone from binomial logistic regressions (20-24 age group is the reference). X-axis in log scale.

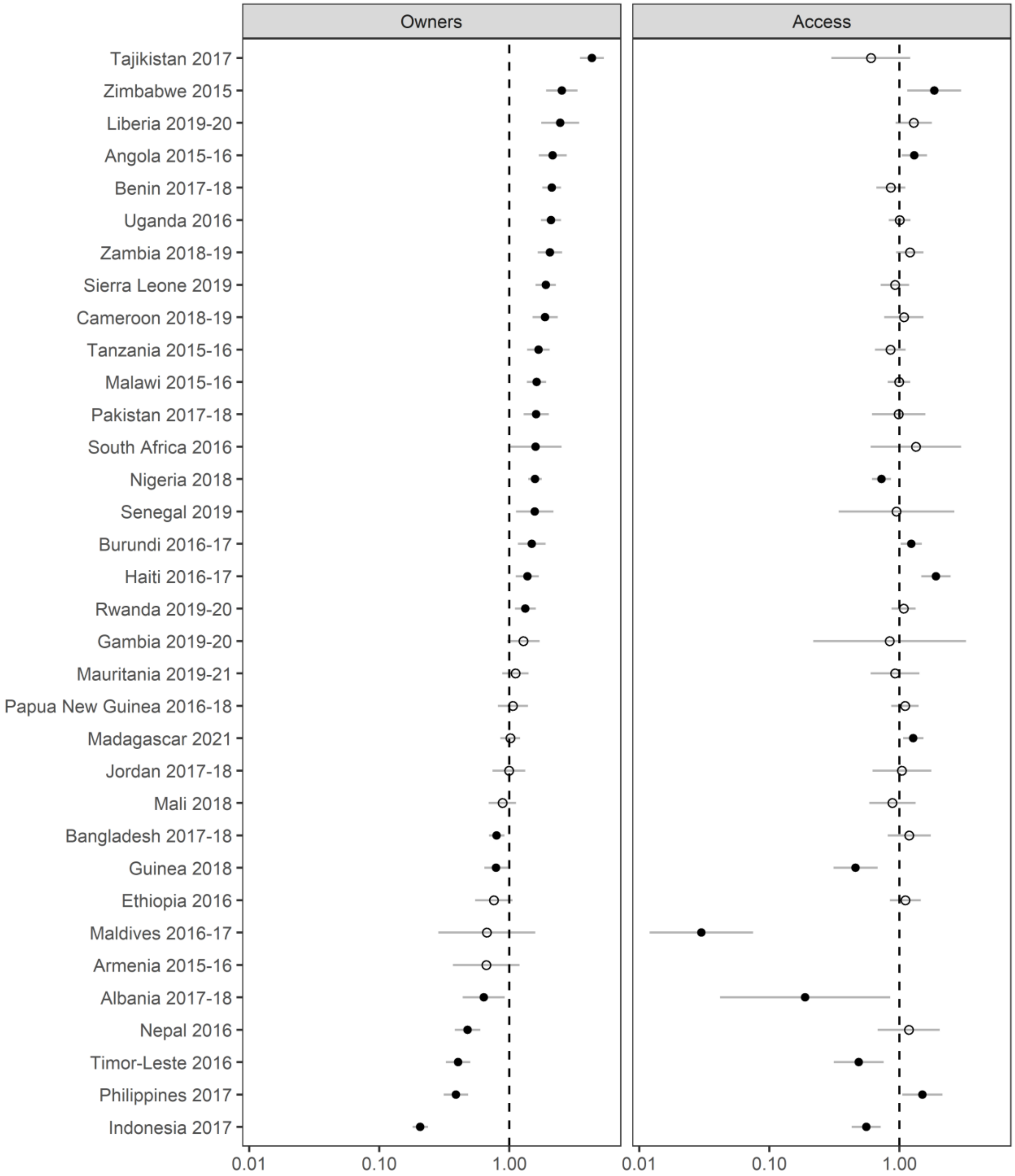

Note: Estimates for access to a mobile phone cannot be computed for Armenia due to small sample size. Fill dots mean that estimates are statistically significant at the 95% confidence level, while empty dots mean that estimates are not statistically significant.

Appendix Figure 2.1: Effect of primary education on the odds of owning and having access to a mobile phone from binomial logistic regressions (no education is the reference). X-axis in log scale.

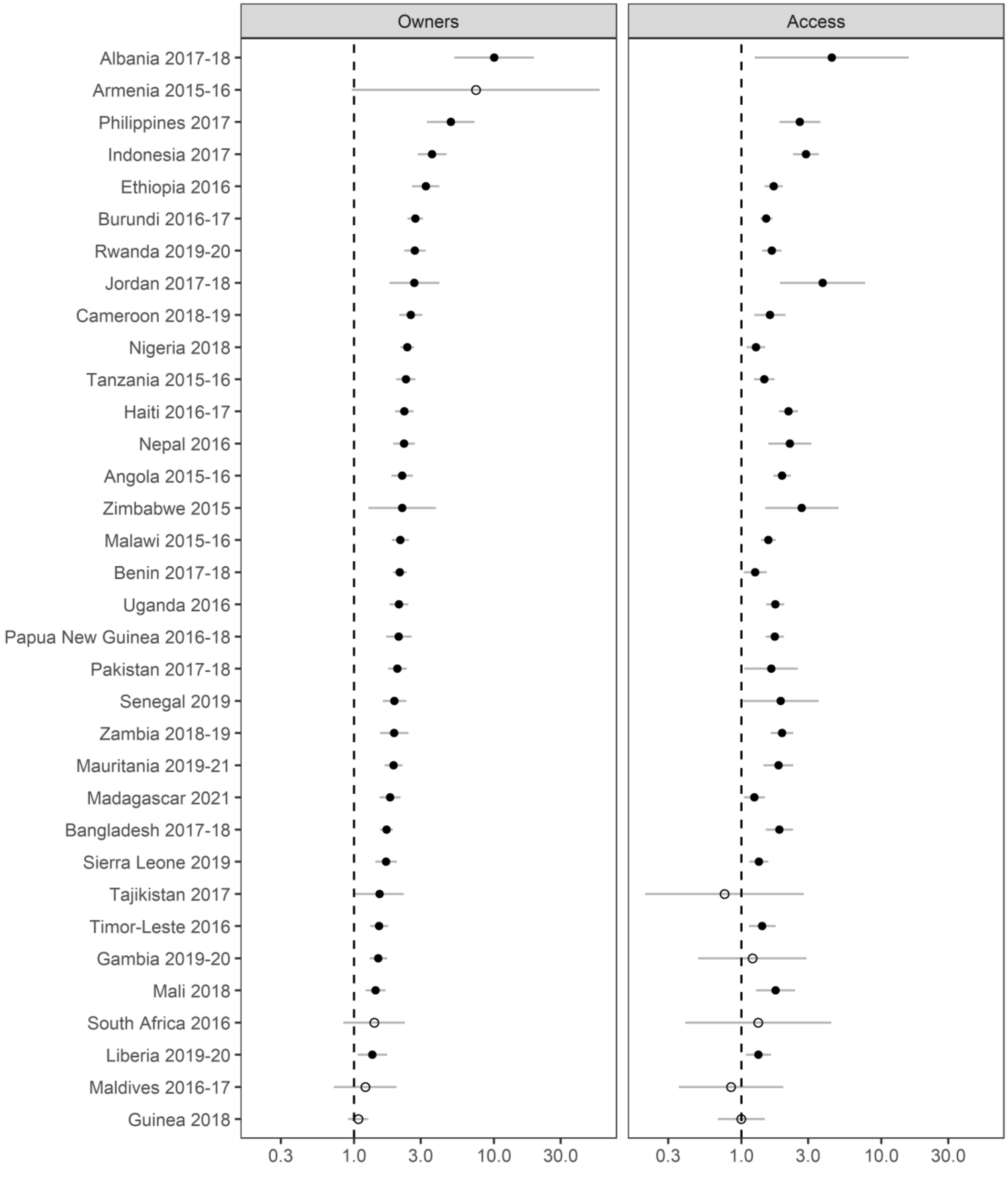

Note: Estimates for access to a mobile phone cannot be computed for Armenia due to small sample size. Fill dots mean that estimates are statistically significant at the 95% confidence level, while empty dots mean that estimates are not statistically significant.

Appendix Figure 2.2: Effect of secondary and higher education on the of odds owning and having access to a mobile phone from binomial logistic regressions (no education is the reference). X-axis in log scale.

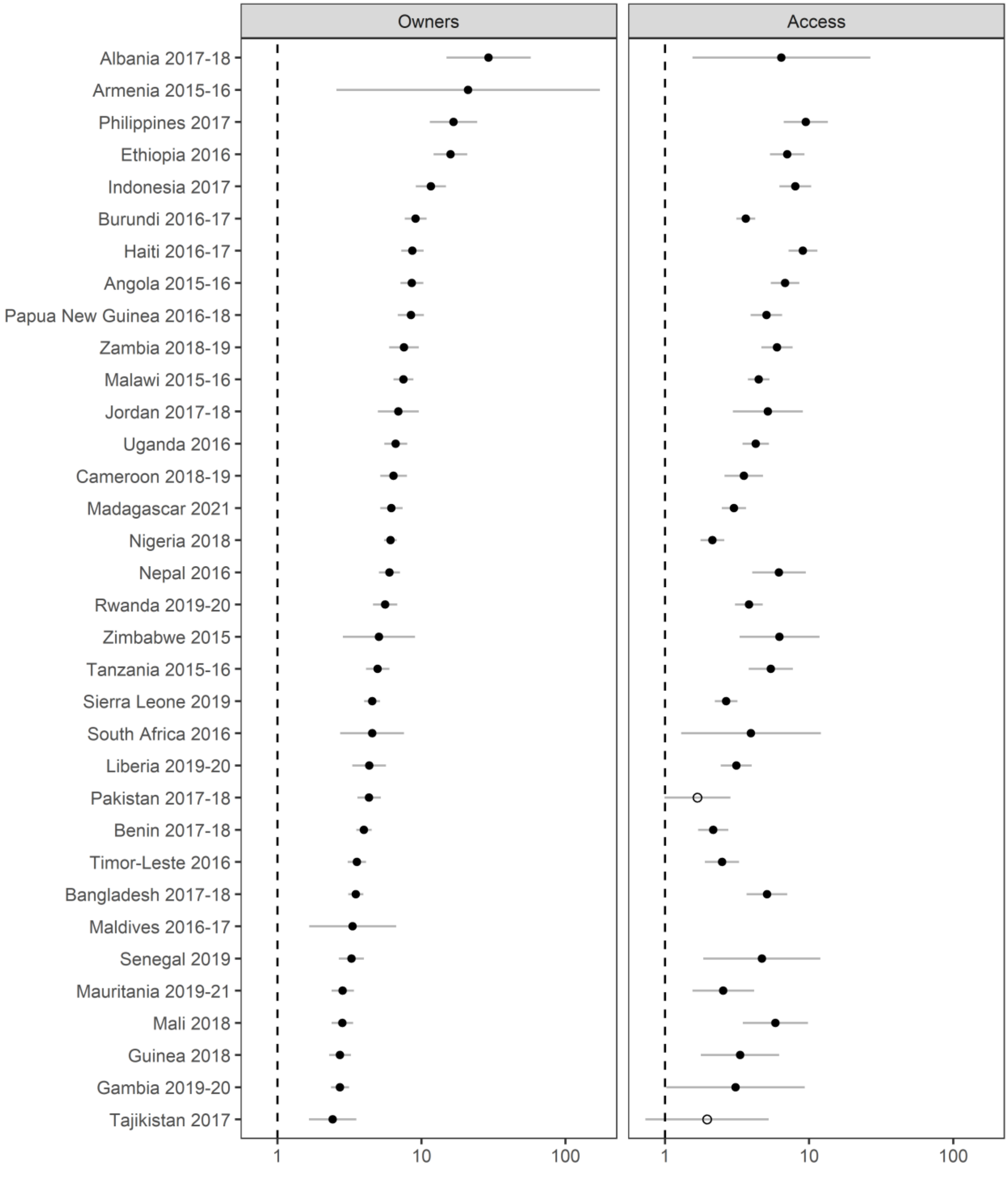

Note: Estimates for access to a mobile phone cannot be computed for Armenia and Maldives due to small sample size. Fill dots mean that estimates are statistically significant at the 95% confidence level, while empty dots mean that estimates are not statistically significant.

Appendix Figure 3: Effect of living in urban areas on the odds of owning and having access to a mobile phone from binomial logistic regressions (rural area is the reference). X-axis in log scale.

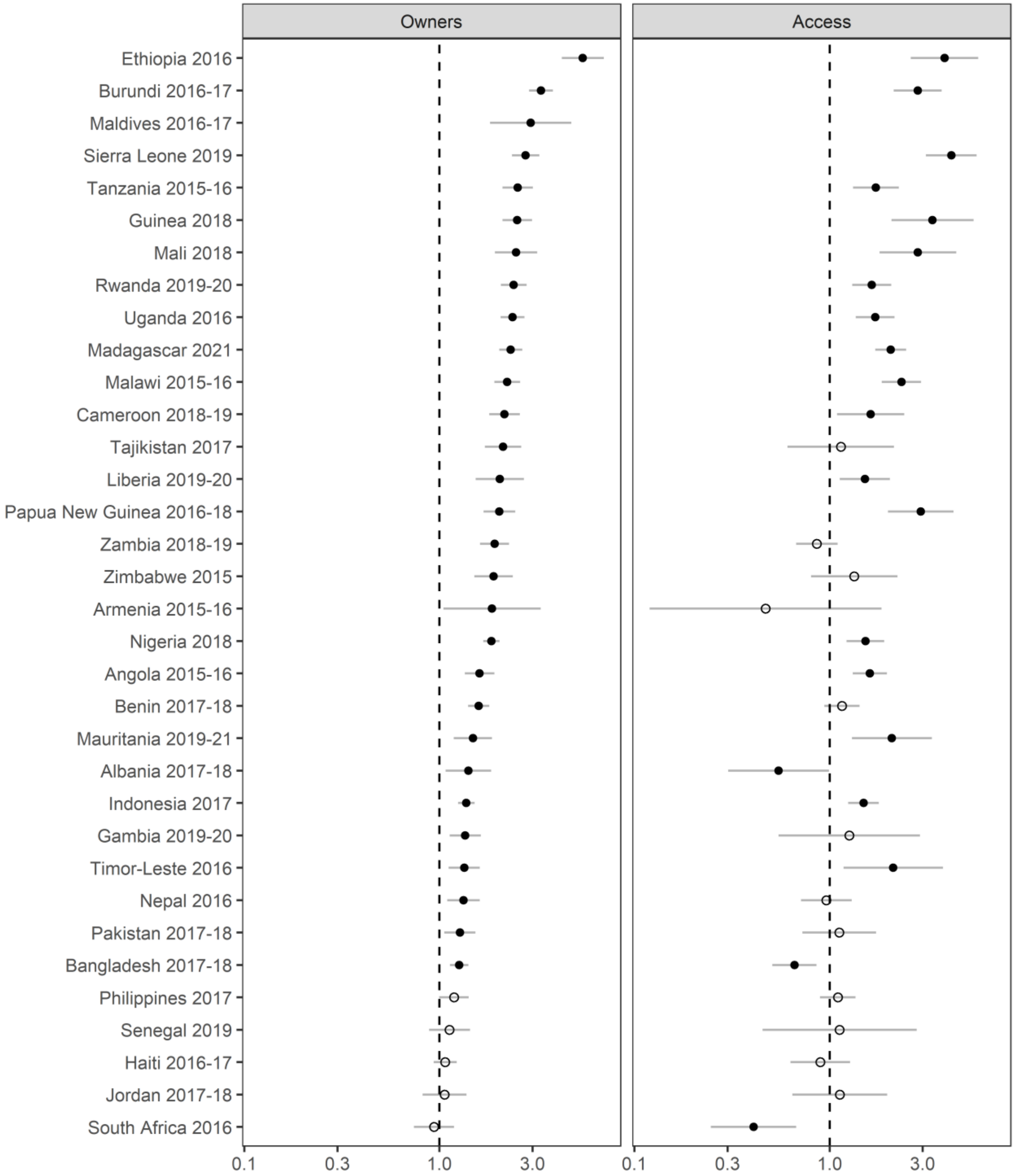

Note: Estimates for access to a mobile phone cannot be computed for Maldives due to small sample size. Fill dots mean that estimates are statistically significant at the 95% confidence level, while empty dots mean that estimates are not statistically significant.

Appendix Figure 4: Effect of being non-poor on the odds of owning and having access to a mobile phone from binomial logistic regressions (poor is the reference). X-axis in log scale.

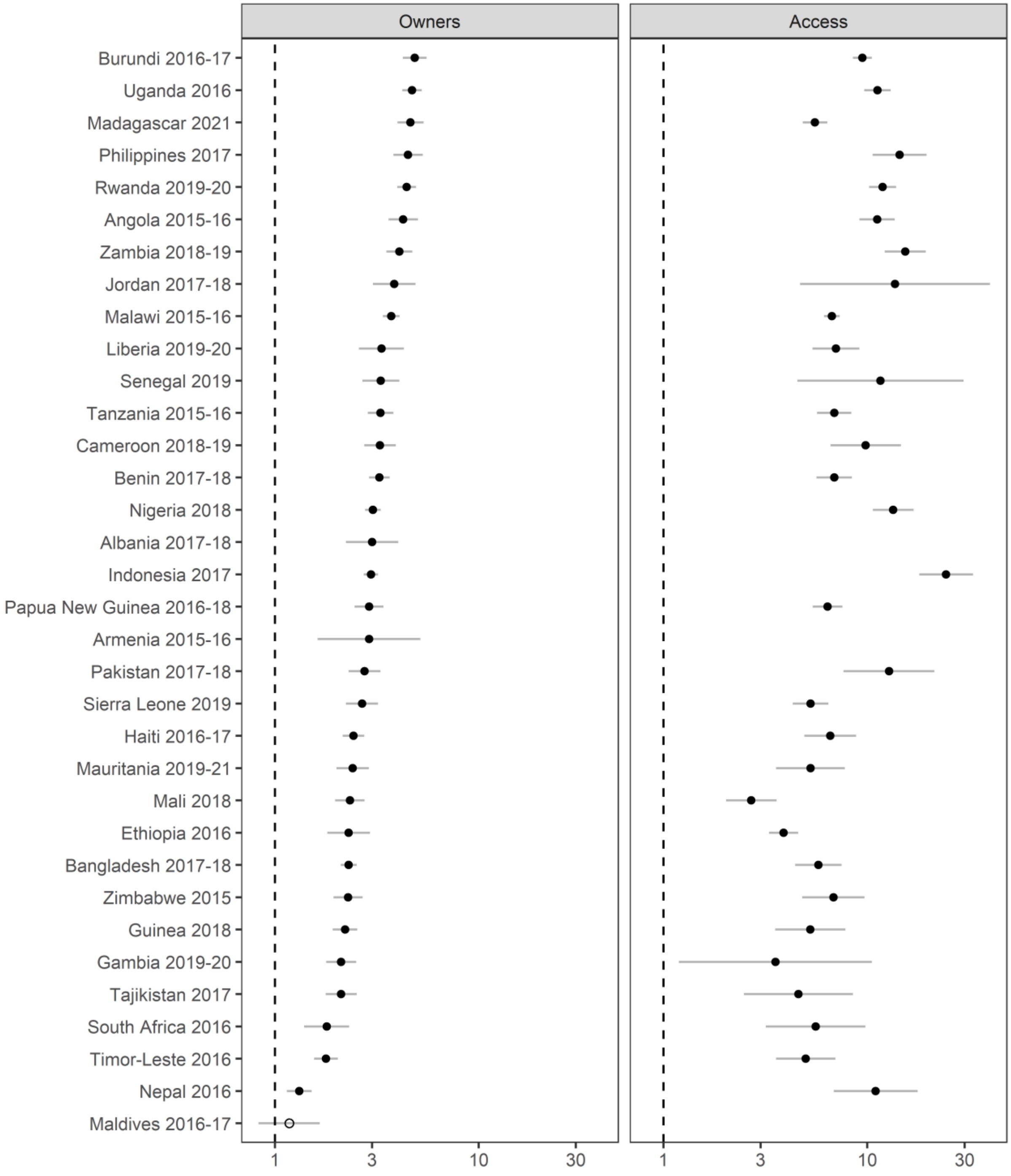

Note: Estimates for access to a mobile phone cannot be computed for Albania, Armenia, and Maldives due to small sample size. Fill dots mean that estimates are statistically significant at the 95% confidence level, while empty dots mean that estimates are not statistically significant.

Appendix Figure 5: Effect of not being in union on the odds of owning and having access to a mobile phone from binomial logistic regressions (married is the reference). X-axis in log scale.

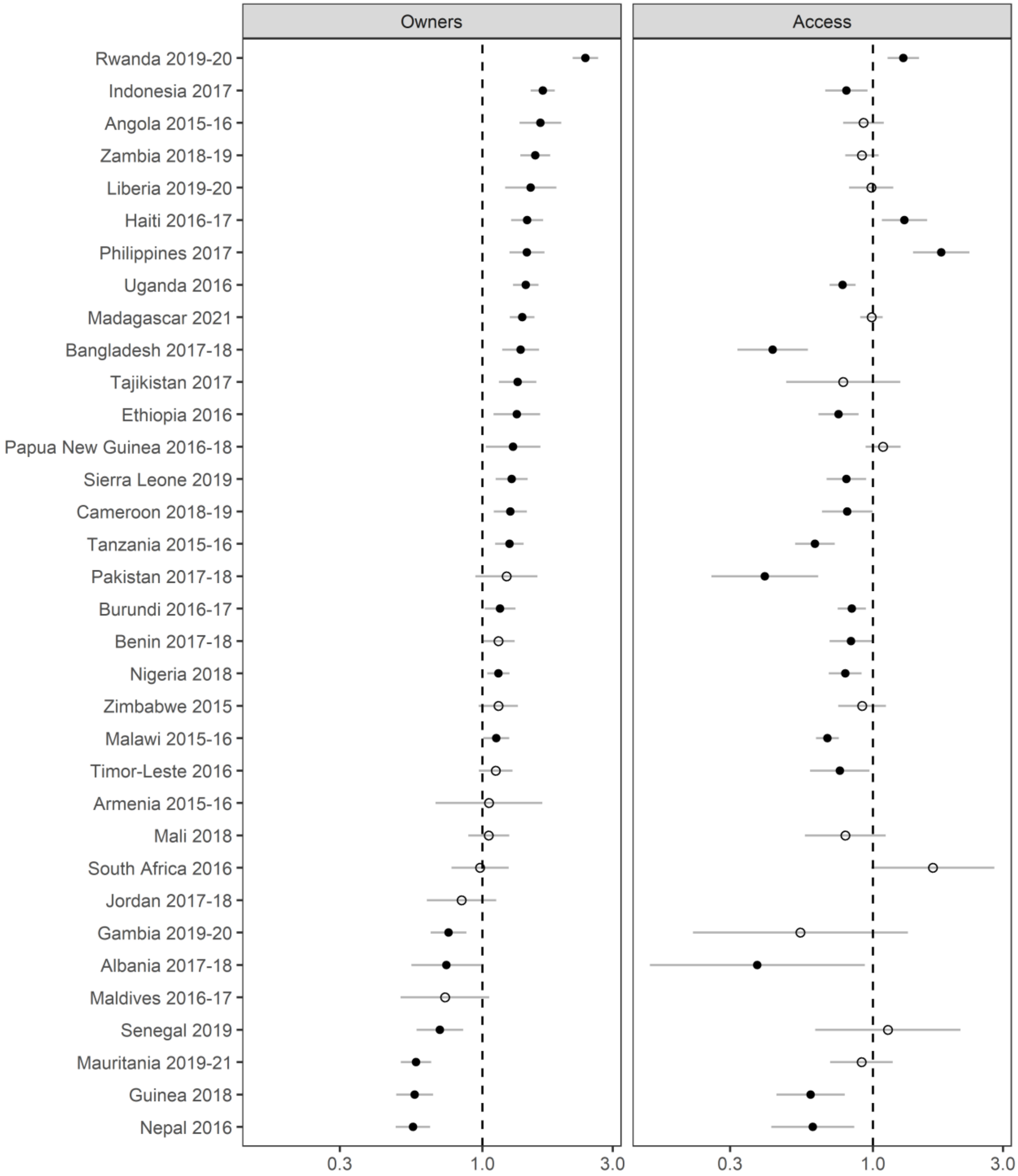

Note: Estimates for access to a mobile phone cannot be computed for Armenia, Jordan, and Maldives due to small sample size. Fill dots mean that estimates are statistically significant at the 95% confidence level, while empty dots mean that estimates are not statistically significant.

Appendix Figure 6: Under-five mortality rate and total fertility rate estimates testing for post-stratification weights from women who have access to a mobile phone.

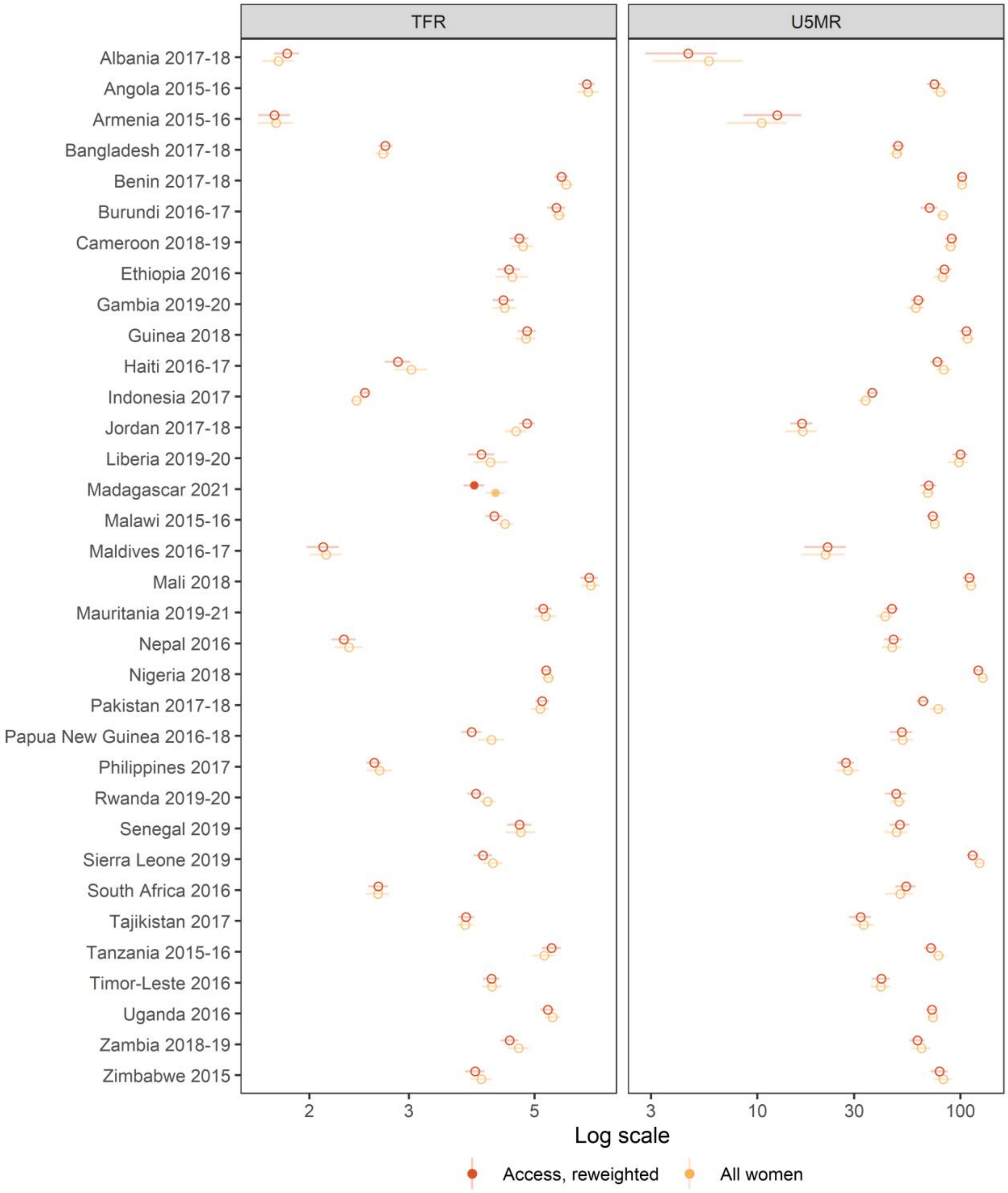

Note: Fill dots mean that the difference between the actual rate and the re-weighted rate is statistically significant at the 95% confidence level, while empty dots mean that the difference is not statistically significant.

Appendix Figure 7: Estimates of the difference in current use of modern contraceptives (MCU), sexual activity (SexAct), duration of postpartum amenorrhea (Amen), and duration of postpartum abstinence (Abst), between having and not having access to a mobile phone.

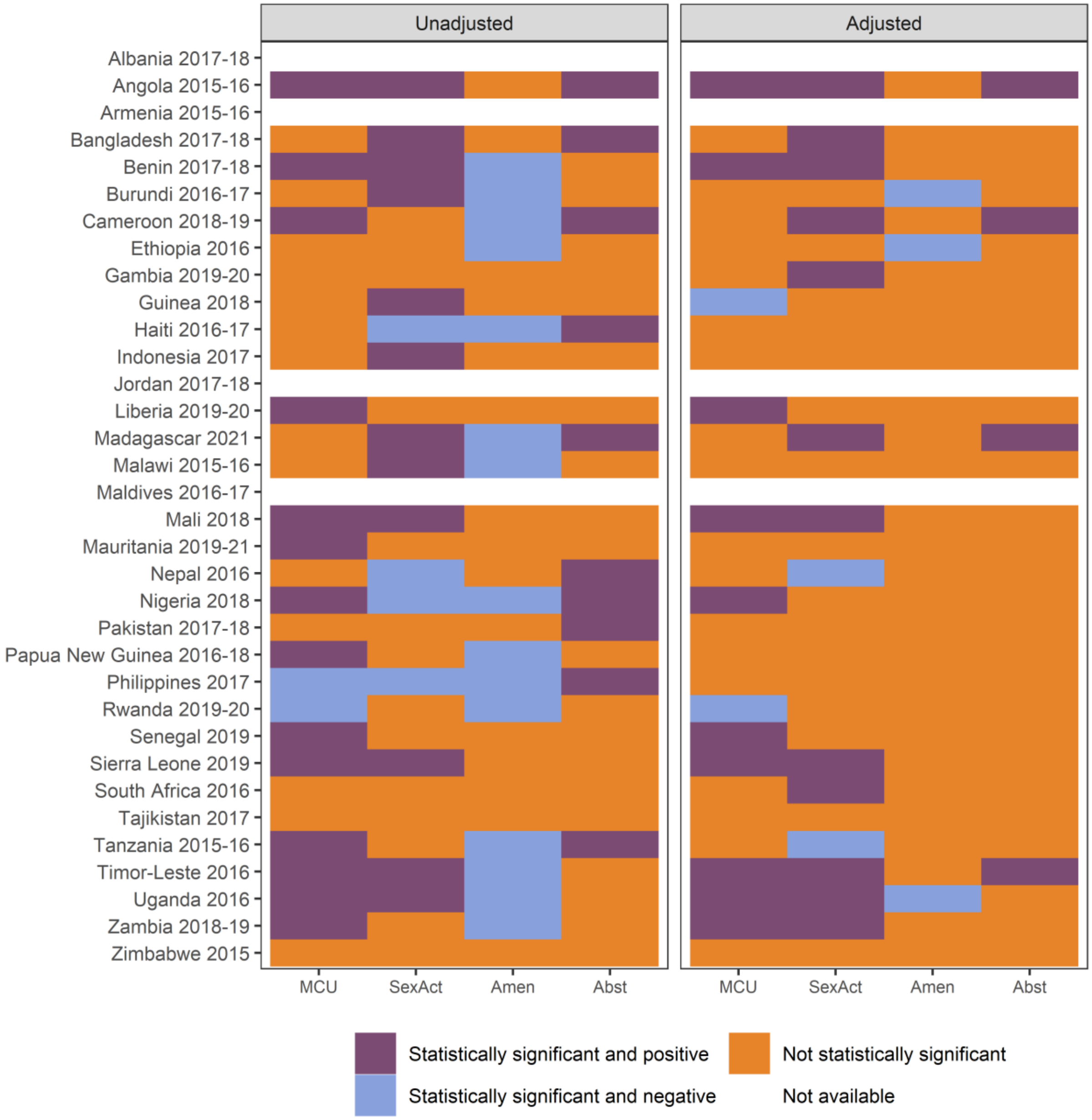

Appendix Figure 8: Estimates of the difference in exclusive breastfeeding (BrFeed), births who had at least 4 antenatal care visits (ANC), newborns who received at least 2 signal functions (PNC), children who received basic immunization (Vax), births delivered by skilled professionals (Skill), children who are underweight (UnderW), between having and not having access to a mobile phone.

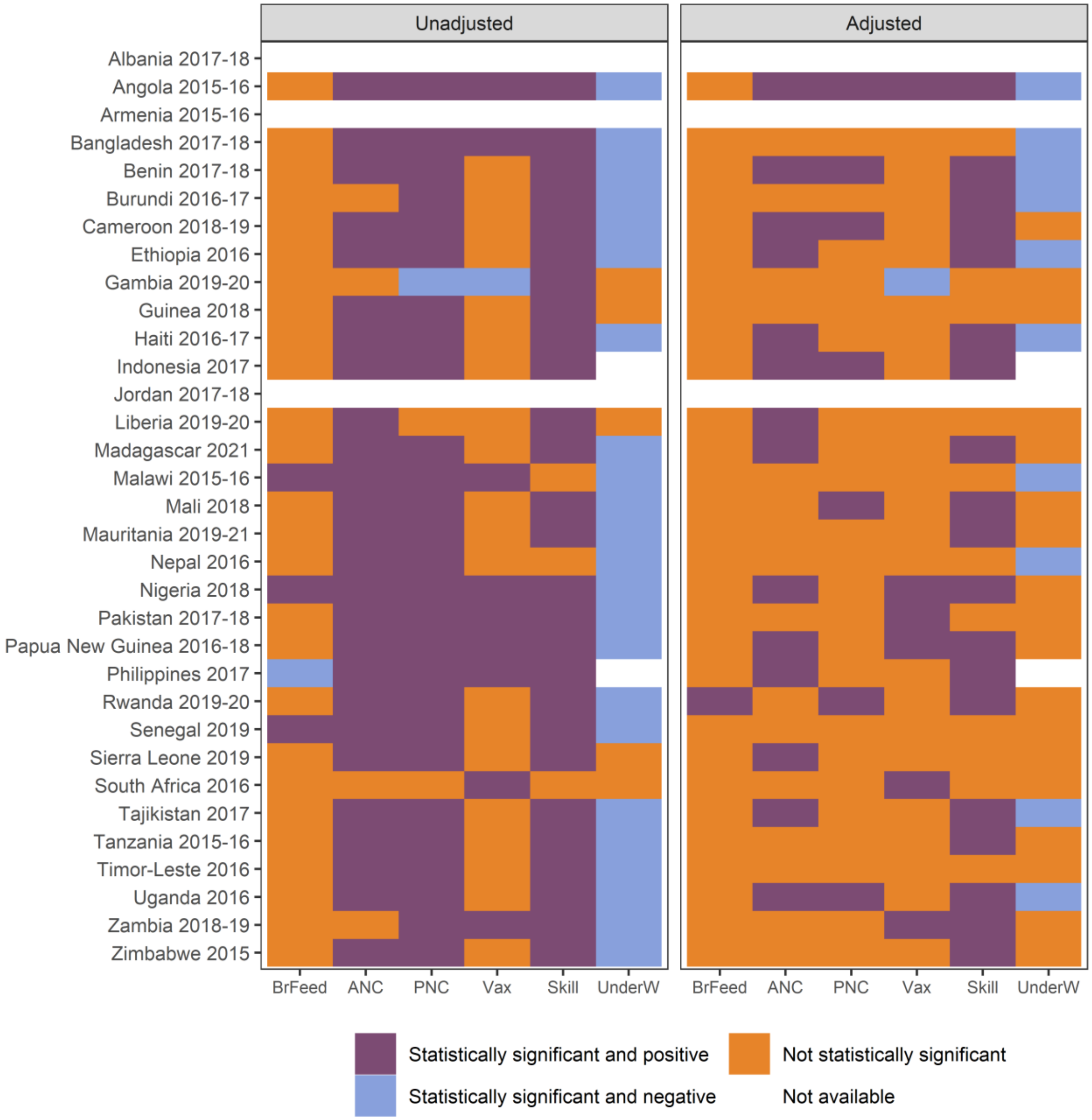

STROBE Statement—Checklist of items that should be included in reports of ***cross-sectional studies***

	Item No	Recommendation	Page No
**Title and abstract**	1	(*a*) Indicate the study's design with a commonly used term in the title or the abstract	2
		(*b*) Provide in the abstract an informative and balanced summary of what was done and what was found	2
**Introduction**			
Background/rationale	2	Explain the scientific background and rationale for the investigation being reported	4-6
Objectives	3	State specific objectives, including any prespecified hypotheses	6
**Methods**			
Study design	4	Present key elements of study design early in the paper	6-8
Setting	5	Describe the setting, locations, and relevant dates, including periods of recruitment, exposure, follow-up, and data collection	7
Participants	6	(*a*) Give the eligibility criteria, and the sources and methods of selection of participants	NA
Variables	7	Clearly define all outcomes, exposures, predictors, potential confounders, and effect modifiers. Give diagnostic criteria, if applicable	7-9
Data sources/ measurement	8*	For each variable of interest, give sources of data and details of methods of assessment (measurement). Describe comparability of assessment methods if there is more than one group	6-7
Bias	9	Describe any efforts to address potential sources of bias	7-9
Study size	10	Explain how the study size was arrived at	6-7
Quantitative variables	11	Explain how quantitative variables were handled in the analyses. If applicable, describe which groupings were chosen and why	7-9
Statistical methods	12	(*a*) Describe all statistical methods, including those used to control for confounding	7-9
		(*b*) Describe any methods used to examine subgroups and interactions	8-9
		(*c*) Explain how missing data were addressed	7
		(*d*) If applicable, describe analytical methods taking account of sampling strategy	NA
		(*e*) Describe any sensitivity analyses	NA
**Results**			
Participants	13*	(a) Report numbers of individuals at each stage of study—eg numbers potentially eligible, examined for eligibility, confirmed eligible, included in the study, completing follow-up, and analysed	NA
		(b) Give reasons for non-participation at each stage	NA
		(c) Consider use of a flow diagram	NA
Descriptive data	14*	(a) Give characteristics of study participants (eg demographic, clinical, social) and information on exposures and potential confounders	21-22
		(b) Indicate number of participants with missing data for each variable of interest	7
Outcome data	15*	Report numbers of outcome events or summary measures	9-10
Main results	16	(*a*) Give unadjusted estimates and, if applicable, confounder-adjusted estimates and their precision (eg, 95% confidence interval). Make clear which confounders were adjusted for and why they were included	10-11

For peer review only - http://bmjopen.bmj.com/site/about/guidelines.xhtml

		(*b*) Report category boundaries when continuous variables were categorized	NA
		(*c*) If relevant, consider translating estimates of relative risk into absolute risk for a meaningful time period	NA
Other analyses	17	Report other analyses done—eg analyses of subgroups and interactions, and sensitivity analyses	11-13
**Discussion**			
Key results	18	Summarise key results with reference to study objectives	13-16
Limitations	19	Discuss limitations of the study, taking into account sources of potential bias or imprecision. Discuss both direction and magnitude of any potential bias	15-16
Interpretation	20	Give a cautious overall interpretation of results considering objectives, limitations, multiplicity of analyses, results from similar studies, and other relevant evidence	16
Generalisability	21	Discuss the generalisability (external validity) of the study results	16
**Other information**			
Funding	22	Give the source of funding and the role of the funders for the present study and, if applicable, for the original study on which the present article is based	16

*Give information separately for exposed and unexposed groups.

Note: An Explanation and Elaboration article discusses each checklist item and gives methodological background and published examples of transparent reporting. The STROBE checklist is best used in conjunction with this article (freely available on the Web sites of PLoS Medicine at http://www.plosmedicine.org/, Annals of Internal Medicine at http://www.annals.org/, and Epidemiology at http://www.epidem.com/). Information on the STROBE Initiative is available at www.strobe-statement.org.

For peer review only
